# Immune cell infiltration into brain tumor microenvironment is mediated by Rab27-regulated vascular wall integrity

**DOI:** 10.1126/sciadv.adr6940

**Published:** 2025-05-23

**Authors:** Lata Adnani, Brian Meehan, Minjun Kim, Dongsic Choi, Christopher E. Rudd, Yasser Riazalhosseini, Janusz Rak

**Affiliations:** ^1^ Research Institute of the McGill University Health Centre, Montreal, QC H4A 3J1, Canada.; ^2^ Victor Phillip Dahdaleh Institute of Genomic Medicine at McGill University. McGill University Department of Human Genetics, Montreal, QC, Canada.; ^3^ Department of Biochemistry, College of Medicine, Soonchunhyang University, Cheonan, Chungcheongnam 31151, Republic of Korea.; ^4^ Division of Immunology-Oncology Research Center, Maisonneuve-Rosemont Hospital, Montreal, QC H1T 2M4, Canada.; ^5^ Département de Medicine, Université de Montréal, Montreal, QC H3C 3J7, Canada.; ^6^ Centre for Translational Research in Cancer, McGill University, Montreal, QC, Canada.; ^7^ Department of Pediatrics, McGill University, Montreal, QC H4A 3J1, Canada.

## Abstract

Aggressive brain tumors often exhibit immunologically ‘cold’ microenvironment, where the vascular barrier impedes effective immunotherapy in poorly understood ways. Tumor vasculature also plays a pivotal role in immunoregulation and antitumor immunity. Here, we show that small GTPase Rab27 controls the vascular morphogenesis and permeability for blood content and immune effectors. Thus, in Rab27a/b double knock out (Rab27-dKO) mice, the brain vasculature is abnormally scarce, while the blood vessels become dysmorphic and hyperpermeable in the context of brain tumors, including syngeneic glioblastoma. These defects are reflected in rearrangements of endothelial cell subpopulations with underlying diminution of venous endothelial subtype along with changes in gene and protein expression. Notably, Rab27-dKO brain endothelial cells exhibit deficient tight junctions, whereby they enable large-scale extravasation of cytotoxic T cells into the tumor mass. We show that Rab27-regulated vascular T cell infiltration can be exploited to enhance adoptive T cell therapy in syngeneic brain tumors.

## Teaser

The vascular barrier in brain cancer can be disrupted by targeting Rab27 to allow immune cell passage and immunotherapy.

## INTRODUCTION

High grade brain tumors, both primary and metastatic, pose formidable therapeutic challenges (*1*). In this regard, the uniqueness of the brain microenvironment and its responses to the neoplastic process have been implicated in many aspects of disease intractability and extensively studied using bulk RNA sequencing (*2*, *3*), single-cell RNA sequencing (scRNAseq) (*4*–*6*), spatial transcriptomics (*7*–*9*), mass spectrometry (*10*–*12*), immunostaining (*2*, *3*), CyToF (*10*, *11*), and other techniques. Paradigmatic in this regard is the cellular landscape of primary astrocytic brain tumors, such as glioblastoma (GBM) (*7*), the cellular milieu of which comprises a multiplicity of noncancer cells including vascular endothelial cells (ECs), pericytes, astrocytes, oligodendrocytes, microglia, and macrophages (*13*–*16*), with notable limitation in the presence of cytotoxic immune effectors, such as natural killer (NK) and CD8α^+^ T cells (*17*, *18*). In addition, immune responses in such brain tumors are often blunted by the inability of T cells to recognize tumor cells, deficient interactions of T cells with antigen presenting cells, the suppressive effects of myeloid cells populating tumor microenvironment, and other factors, as recently reviewed (*19*). The immunologically “cold” GBM microenvironment (*17*, *20*–*23*) is also regarded as one of the main reasons why attempts at systemic immunotherapy of GBM, including immune checkpoint inhibitors, have yet to produce major gains in patient survival (*24*).

Several studies have explored adoptive immune cell therapies based on systemic delivery of either unmodified or engineered cytotoxic cells, such as CAR-T or CAR-NK cells, to overcome the paucity of endogenous immune effectors in brain tumors. The limited therapeutic successes of these therapies, thus far, could be attributed, in part, to poor penetration of immune effectors into the brain tumor microenvironment (*25*). Obstacles that may prevent the entry of circulating immune cells into the brain parenchyma may include factors such as EC anergy (*26*), blood-tumor barrier, and blood-brain barrier (BBB) (*1*, *27*). BBB is made up of brain vascular ECs, acting in concert with pericytes and astrocytic foot processes, collectively shielding the delicate brain tissue from systemic and external insults (*28*). A major structural determinant of the BBB integrity is formation of interendothelial tight junction (TJ) complexes. TJs are composed of a complex microanatomical and signaling structures including integral membrane proteins (occludins and claudins) and their intracellular interactors, of which zonula occludens (ZO) proteins represent one of the main subsets (*28*, *29*). TJs are linked to the cytoskeleton and control vascular wall stability while also playing a major role in para-endothelial molecular and cellular transport (*26*, *29*).

The upstream molecular regulators of BBB include paracrine growth factor pathways, such as those activated by vascular endothelial growth factor (VEGF) (*27*), or WNT (*30*), but far less is understood about the regulatory roles of intracellular signal processing machineries, such as the endosome, known to influence TJ proteins (*31*, *32*). Of interest, endosomal Rab27a guanosine triphosphatase has been recently demonstrated to control the stability of VEGF receptors thereby affecting angiogenic responses of ECs (*33*). Rab27 interacts with multiple cellular proteins (*34*), controls endothelial secretory pathway (*35*), and is involved in the biogenesis of exosomes, a subset of extracellular vesicles (EVs) derived from the late endosome (*36*) and implicated in vascular responses (*37*, *38*).

Rab27 proteins include two different isoforms, Rab27a and Rab27b (*36*), which are thought to have partially nonredundant, secretory, hemostatic, and immuno-regulatory functions. For example, Griscelli syndrome is linked to the loss-of-function mutation of *RAB27A* gene, and the affected individuals exhibit propensity for infections, neurological symptoms, and abnormal pigmentation (*39*). Similarly, the abnormality of pigmentation and deficient immune responses are observed in mice with spontaneous loss of function of Rab27a (*Rab27a^ashen^*) mutation, while mice with disrupted *Rab27b* gene exhibit mainly a hemorrhagic state due to defective degranulation of platelets, which is also observed in double-deficient, *Rab27a^ash/ash^;Rab27b−/−* (Rab27-dKO) animals (*40*). Notably, *Rab27b* was shown to be up-regulated in leukocytes following loss of *Rab27a*, suggesting a degree of interrelatedness between these two protein isoforms (*41*). Thus, Rab27 proteins appear to occupy a crucial regulatory node at the crossroads of vascular and immune regulation, and we reasoned that they could be informative as to the paradoxically hypervascular and hypoimmune state often associated with aggressive brain tumors (*1*).

Here, we show that Rab27 plays a pivotal role in vascular wall regulation and immune cell ingress, surveillance, and adoptive immunotherapy in the context of aggressive brain tumors in mice. Thus, Rab27a/b-deficient mice exhibit a hitherto unrecognized vascular phenotype marked by scarce capillary networks in the intact adult brain and dysmorphic and hyperpermeable vasculature associated with aggressive tumor growth. Unlike their normal counterparts, blood vessels in tumor bearing Rab27-deficient mice exhibit altered ECs landscapes with distinct gene and protein expression profiles and poorly developed TJs. These Rab27-dependent vascular features are accompanied by a pronounced extravasation of immune cells, resulting in formation of immunologically “hot” brain tumor microenvironment. Using a pharmacological TJ inhibitor, which mimics Rab27 deficiency, we show a higher T cells infiltration into the otherwise “cold” brain tumor microenvironment. We document that Rab27-dependent vascular conversion renders syngeneic brain tumors susceptible to adoptive T cell therapy (*42*, *43*). Together, these observations reveal the previously unknown function of Rab27 in regulating vascular wall integrity, and they point to the possibility of targeting Rab27-related pathways to augment the responses to immunotherapy in intractable brain tumors, including GBM.

## RESULTS

### Rab27 regulates vascular morphogenesis in the brain

**Fig. 1.**
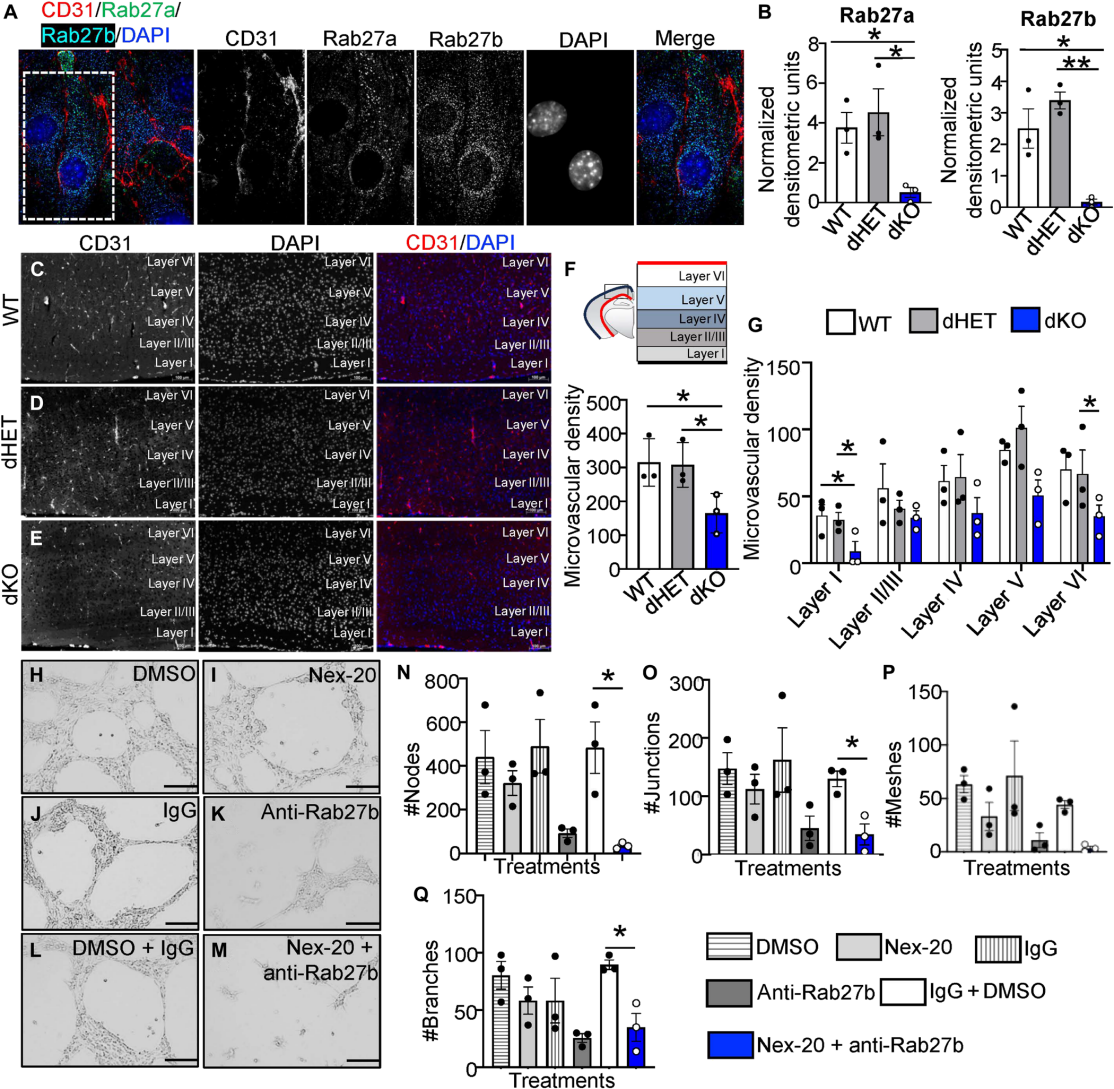
Rab27 controls vascular morphogenesis in the brain. (**A**) Immunostaining of primary BECs isolated from Rab27-WT mice for CD31 (red), Rab27a (green), and Rab27b (cyan), with 4′,6-diamidino-2-phenylindole (DAPI) (blue) counterstain, imaged at 63× using super-resolution microscopy (SIM). (**B**) Western blot normalized quantifications to study levels of Rab27a and Rab27b in WT (white), dHET (gray), and dKO (blue) primary BEC isolates. (**C** to **E**) Immunostaining of the vasculature using CD31 (red) and DAPI (blue) counterstain on brain tissues derived from Rab27-WT (C), Rab27-dHET (D), and Rab27-dKO (E) mice. (**F**) Cartoon to show murine neocortex (top) and quantification of total microvascular density in the neocortex (bottom) of WT (white), dHET (gray), and dKO (blue) mice. (**G**) Layer-related microvascular density counts in the neocortex of WT (white), dHET (gray), and dKO (blue) mice. (**H** to **M**) Tube formation assay using primary WT-BECs treated with DMSO (H), 1 μM Nex-20 (I), 2.7 μg of IgG (J), 2.7 μg of anti-Rab27b antibody (K), DMSO + IgG (L), and 1 μM Nex-20 + 2.7 μg of anti-Rab27b antibody (M). (**N** to **Q**) Quantifications of the number of nodes (N), junctions (O), meshes (P), and branches (Q), formed by primary WT-BECs after 15 hours in culture. WT, wild type (C57bl/6); dHET, Rab27a/b double heterozygote; dKO, Rab27a/b double knockout; *P < 0.05 and **P < 0.01. Scale bars, 100 µm.

Rab27 proteins lie at the crossroads of multiple regulatory and cell communication pathways, but their role in the vasculature remains poorly understood. To glean new insights as to the possible role of Rab27 proteins in regulating blood vessels in the brain, we first confirmed the expression of these proteins in primary brain ECs (BECs) isolated from Rab27a/b wild type (WT), double heterozygous (dHET), and double-deficient knockout (dKO) mice (Fig. 1, A and B). Next, we explored whether Rab27 deficiency leads to any vascular consequences in vivo. To do so, we first analyzed vascular patterns in the brains of WT, dHET, and dKO adult mice following the staining of ECs with anti-CD31 antibody (Fig. 1, C to E). Strikingly, Rab27 deficiency (dKO) resulted in markedly lower microvascular density counts in the adult neocortex across all layers, relative to both WT and dHET controls (Fig. 1, F and G). This is interesting, as the brain microvasculature arises during development, mainly as a function of angiogenesis, a process that could, we reasoned, be controlled by Rab27 proteins affecting ECs either directly (*27*) or indirectly (*40*).

To address the first possibility, we established angiogenesis-mimicking tube formation assay in vitro using purified ECs and agents that perturb Rab27 function (*44*). We used a known inhibitor for Rab27a [Nexinhib-20 (Nex-20) (*45*)] and anti-Rab27b antibody to inhibit Rab27b activity (fig. S1, A to C). We confirmed the ability of mouse BECs to uptake anti-Rab27b antibody by macropinocytosis by inhibiting this process pharmacologically (using EIPA) before exposing BECs to the fluorescently labeled antibody (fig. S2). BECs were then plated in Matrigel in the presence of Nex-20, anti-Rab27b antibody, or both versus the respective controls [dimethyl sulfoxide (DMSO) and immunoglobulin G (IgG)], and the assembly of vascular-like networks was observed and quantified (Fig. 1, H to Q). Both Rab27a and Rab27b inhibitors disrupted endothelial network formation including diminution of nodes, junctions, meshes, and branching connections (Fig. 1, N to Q). Although the effect of anti-Rab27b antibody was relatively robust, the Nex-20 + anti-Rab27b antibody combination exerted an even more pronounced impact on endothelial network formation (Fig. 1, L to Q). Therefore, we reasoned that both Rab27a and Rab27b likely contribute to endothelial responses, and double targeting was adopted in further studies.

**Fig. 2.**
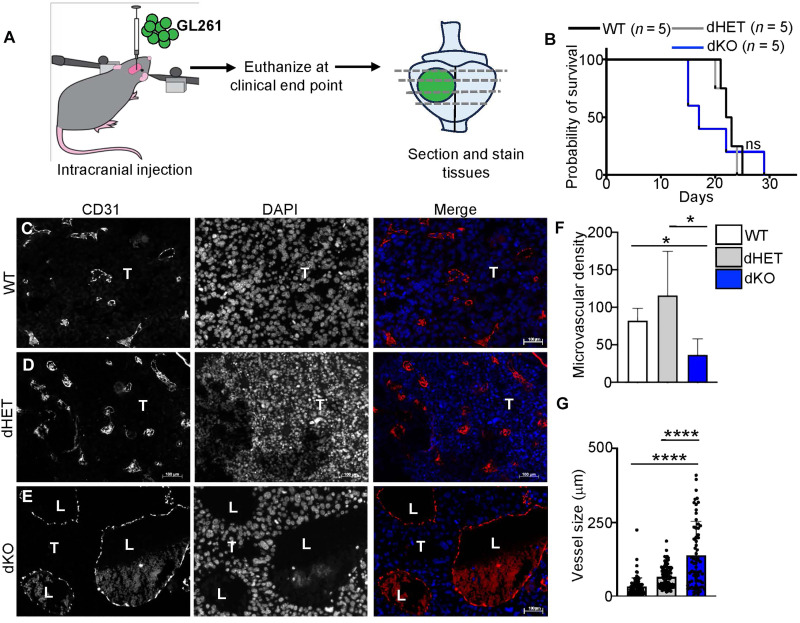
Rab27 deficiency leads to dysmorphic vasculature in murine GBM. (**A**) Schematic of experimental design. (**B**) Kaplan-Meyer survival curve of WT (black), dHET (gray), and dKO (blue) mice injected intracranially with GL261 murine glioma cells and monitored for survival. *n* = 5 in each group. (**C** to **E**) Immunostaining of brain glioma tissues for CD31 in WT (C), dHET (D), and dKO (E). (**F**) Quantification of microvascular density for brain tumor tissues of WT (white), dHET (gray), and dKO (blue) GL261 tumor models. (**G**) Quantification of tumor blood vessel size/diameter in WT (white), dHET (gray), or dKO (blue) mice bearing GL261 intracranial lesions. WT, wild type (C57bl/6); dHET, Rab27a/b double heterozygote; dKO, Rab27a/b double knockout; T, tumor; L, vascular lumen; not significant (ns); *P < 0.05 and ****P < 0.0001.

The reduced vascular density in Rab27-deficient mice (dKO) does not necessarily predict vascular responses in the context of brain tumors where highly pro-angiogenic microenvironment may overwhelm the intrinsic control mechanisms (*27*). To assess the consequences of Rab27a/b deletion in vivo, we engrafted GL261 syngeneic (GBM-like) glioma cells intracranially into the right striatum of either WT, dHET, or dKO mice (Fig. 2A) and monitored tumor progression and vascularity (Fig. 2, B to G, and fig. S3). Although the status of Rab27 did not influence the survival of mice bearing aggressive GL261 lesions (Fig. 2B), it markedly affected tumor vascular patterns. Tumors in the Rab27-deficient (dKO) mice exhibited strikingly enlarged and dysmorphic vascular channels with poorly defined vascular walls, while the density of capillary microvessels was markedly reduced, as compared to WT and dHET controls (Fig. 2, C to G). We also observed a similar pattern in an unrelated brain tumor model, where the intracranial injection of aggressive murine breast cancer cells (E0771) was used to emulate metastasis to the brain (fig. S4), an observation further pointing to host-specific causes of vascular dysmorphia. Collectively, these results suggest that in the presence of the same cancer cells, the blood vessel forming potential of BECs is affected by the host systemic Rab27 expression.

### Dysmorphic tumor vasculature persists upon systemic transfer of Rab27-postitive bone marrow

While endothelial morphogenesis in the brain was markedly affected, as a function of Rab27 status, this does not exclude a role for Rab27-deficient non-ECs (platelets or myeloid cells) known to regulate tumor angiogenesis (*40*, *46*). To address this question, we first measured the levels of platelets along with levels of hemoglobin, hematocrit, neutrophils, lymphocytes, and eosinophils, including total white blood cell (WBC) and red blood cell (RBC) counts, as well as monocytes in mice proficient or deficient for Rab27 (fig. S5). These assays revealed minimal or no consistent numerical differences.

To account for the known functional impact of Rab27 deficiency on hematopoietic cell compartments (*40*), we also chose to perform bone marrow exchange experiments (chimera) (*47*). We aimed to replace endogenous Rab27-proficient (or -deficient) bone marrow cell populations that give rise to hematopoietic effector cells (platelets and myeloid cells) with their respective Rab27-deficient (or -proficient) counterparts to correspondingly induce (or prevent) brain tumor vascular dysmorphia (fig. S6A). Thus, stable bone marrow chimera and control mice were prepared and validated (fig. S6, B to E), including transplants of green fluorescent protein–positive (GFP^+^) bone marrow from C57bl/6-GFP donors to Rab27-dKO mice (fig. S6, F and G). GL261 tumor cells were inoculated intracranially into the respective chimeric or control hosts, and vascular patterns were examined (figs. S6 and S7). While subtle changes were noted in the brain cellular architecture of bone marrow recipients, we were unable to rescue the vascular dysmorphia observed in the dKO tumor microenvironment upon replacement of the endogenous bone marrow with Rab27-proficient cells (fig. S8). These results further suggest that the aberrant vascular remodeling occurring in brain tumors of Rab27-deficient mice is likely related, at least in part, to the properties of brain-resident ECs rather than the superimposed hematological defects.

### Abnormal transcriptional profiles of ECs harboring Rab27 deficiency

**Fig. 3.**
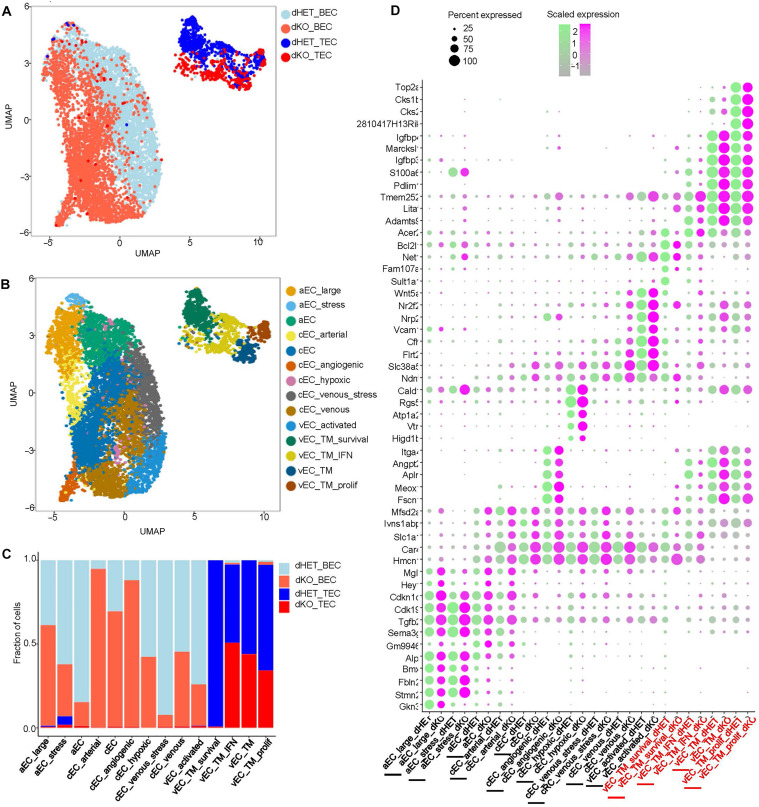
Rab27 deficiency leads to abnormal transcriptional phenotypes of EC subpopulations. (**A**) UMAP plot for scRNAseq for BECs and TECs in mice with either dHET or dKO genotype. (**B**) UMAP plot for the different cell-type clusters previously reported (*49*). (**C**) Bar graph to compare the percentage of genes observed across the different cell-type clusters in BECs and TECs. (**D**) Dot plot to compare the five most differentially regulated genes in the different subsets of ECs. The differentially regulated genes are listed on the *y* axis, and the different EC-type clusters for either dHET (green) or dKO (pink) are on the *x* axis. The red font on labels for the *x* axis indicates the tumor-derived groups of ECs for dHET or dKO, respectively. The size of each dot is based on the percent of gene expression as is shown in the key. BECs, primary BECs; TECs, primary tumor-derived BECs; aEC, arterial ECs; cEC, capillary ECs; cEC_arterial, capillary ECs close to the arterial branches; cEC_venous, capillary ECs close to the veins; vEC, vein ECs; aEC_large, large artery ECs; aEC_stress, arterial ECs with features of stress response; cEC_angiogenic, angiogenic capillary ECs; cEC_venous_stress, capillary ECs with venous-like phenotype and features of stress response; vEC_activated, activated venous ECs; vEC_TM_survival, venous ECs specific for the tumor microenvironment enriched for markers of cell survival; vEC_TM_IFN, venous ECs with features reflective of tumor microenvironment enriched for markers of inflammation; vEC_TM, venous ECs with tumor-associated characteristics; vEC_TM_prolif, venous ECs with features specific for the tumor microenvironment enriched in proliferation markers.

Aberrant vascular morphogenesis in cancer is often a result of perturbations in the endothelial differentiation programs and formation of their distinctive subpopulations (*48*). To assess this aspect of Rab27-dependent vascular phenotype, we first directly studied RNA expression levels of Rab27a and Rab27b in ECs using fluorescent in situ hybridization, which documented a robust and widespread presence of these transcripts in both BECs and bEnd.3 cells (figs. S9 and S10). We then performed scRNAseq of primary ECs isolated either from normal mouse brains (BECs) or from brain tumor (GL261) vasculatures [tumor endothelial cells (TECs)] using anti-CD31–based immunoaffinity purification approach (Fig. 3 and figs. S11 to S16). Overall, dimensionality reduction plots revealed that BECs and TECs formed readily separable clusters (Fig. 3A and figs. S11 and S12), the latter being less diverse than previously reported (*48*) but clearly distinguishable between dHET and dKO samples (Fig. 3A). BEC and TEC populations exhibited previously defined (*49*) signatures of vein, artery, and capillary ECs (Fig. 3B and fig. S13, A to C), as well as functional aspects of EC subsets, such as angiogenic, proliferative, and inflammatory signatures in both dHET and dKO mice (fig. S13, D to F), along with pan BEC markers such as *Cldn5*, *Cdh5*, *Pecam1*, *Tjp1*, *Abcb1a*, *Epas1*, *Flt1*, and *Slc2a1* (fig. S14). None of the canonical pericyte markers—such as *Kcnj8*, *Cspg4*, and *Pdgfrb*—were readily detected in these preparations confirming successful purification (fig. S14). Of interest, venous signature dominated the phenotype of TECs especially in Rab27-proficient mice (Fig. 3C and fig. S15). However, in BECs, loss of Rab27 was associated with increased percentage of capillary ECs including those with angiogenic and arterial-like signatures (*50*, *51*), while capillary ECs with vein-like signatures and activated venous-type ECs were underrepresented (Fig. 3, C and D, and fig. S16). In brain tumor-associated TECs of dKO mice, there was a notable paucity of certain canonical endothelial signatures including arterial, capillary, and angiogenic cells, as well as cells with features of tumor venous endothelium expressing genes linked to cell survival; the latter category was pronounced in Rab27-proficient TECs. Likewise, although some TECs in dKO mice expressed venous characteristics including those linked with proliferation and inflammatory responses, these categories of ECs were more prominent in the Rab27-proficient TECs (vEC_TM_IFN; Fig. 3, C and D, and fig. S16). Together, these observations suggest that in Rab27-deficient mice, BECs exhibit features suggestive of delayed completion of angiogenic vascularization. In response to the neoplastic growth process the Rab27-deficient brain vasculature not only formed dysmorphic networks but also exhibited further alterations in molecular traits and in EC landscapes.

### Defect of endothelial TJs accompanies Rab27 deficiency

**Fig. 4.**
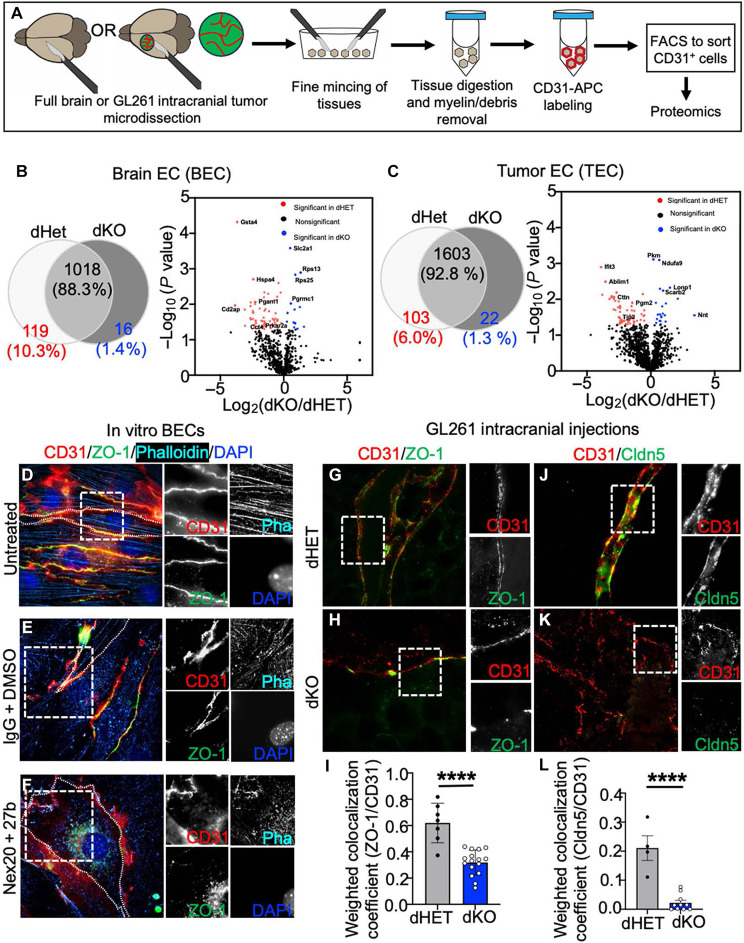
In Rab27-deficient mice, tumor blood vessels lose junctional structures. (**A**) Schematic of BEC isolation by FACS using anti-CD31^+^ antibody in preparation for mass spectrometry. (**B**) Left: Venn diagram to show the number of identified EC proteins in BEC isolates from either dHET or dKO mice. Protein threshold of 99.0%, peptide threshold of 95%, and minimum of 1 peptide in Scaffold program. Label-free quantitation was conducted by normalized total spectra in Scaffold. Right: Volcano plot to show the substantially affected proteins in the proteomes of dHET- or dKO-derived BECs. (**C**) Left: Venn diagram to show the number of identified endothelial proteins in TEC isolates of dHET and dKO mice harboring GL261 brain tumors. Protein threshold of 99.0%, peptide threshold of 95%, and minimum of 1 peptide in Scaffold program. Label-free quantitation is conducted by normalized total spectra in Scaffold. Right: Volcano plot to show the substantially affected proteins in TECs isolated from either dHET or dKO tumor bearing mice. (**D** to **F**) Validation of selected proteins down-regulated in the dKO-BEC proteome relative to dHET-BEC proteome. Immunofluorescence staining for CD31, ZO-1, phalloidin, and DAPI counterstain in untreated Rab27-WT–derived BECs (D), Rab27-WT–derived BECs treated with either IgG + DMSO (E) or with 1 μM Nex20 + (1:100) anti-Rab27b antibody (F). (**G** and **H**) GL261 brain tumor tissue sections stained for CD31 and ZO-1. Tissues were isolated from tumors in dHET (G) or dKO (H) mice. (**I**) Quantification of colocalization between CD31 and ZO-1 in GL261 brain tumors. (**J** and **K**) GL261 brain tumor tissue sections stained for CD31 and Cldn5, following tumor formation in either dHET (J) or dKO (K) mice. (**L**) Quantification of colocalization between CD31 and Cldn5 brain tumor bearing mice. Images were procured using the super-resolution microscope at 63× and processed by structured illumination (SIM) calculation, followed by maximum intensity projection to combine the z stacks; ****P < 0.0001.

To better understand the nature of altered EC traits in Rab27-deficient mice, we performed mass spectrometry on primary BECs (Fig. 4A) isolated from either Rab27-proficient or Rab27-deficient animals (Fig. 4B and fig. S17), and we compared them to the corresponding TECs (from GL261 glioma) (Fig. 4C and fig. S18). While we observed certain proteomic similarities across the BEC and TEC cellular populations (Fig. 4, B and C, and figs. S17 and S18), the absence of Rab27 imposed considerable qualitative and quantitative changes, including proteins linked to leukocyte trans-endothelial migration, TJ, and adherens junction pathways (figs. S17 to S21).

The down-regulation of junctional proteins in ECs upon Rab27 disruption is relatively unstudied, intriguing (*52*), and of interest in the context of our observations, as these structures control the integrity and transmissibility of the vascular wall (*1*). It is known that Rab27 is involved not only in generating actin tracks (*53*) but also in regulating protein transport in the cytoplasm along actin filaments (*54*). Rab27 deficiency is also linked to abnormal F-actin distribution (*55*–*59*). These roles of Rab27 in subcellular organization were reinforced by visualization of protein-protein interaction networks affected by down-regulation of actin (figs. S19A, S20A, and S21, C and D), cytoskeletal molecules (fig. S21, A and B), and TJ regulatory molecules (fig. S21, C and D) in Rab27-deficient ECs in our proteomic datasets.

To better understand the possible link between these subcellular structures and the role of Rab27 in vascular patterning, BECs were stained for ZO-1 (anti-Tjp1), F-actin (phalloidin), and membrane-associated CD31 (anti–platelet endothelial cell adhesion molecule), following pharmacological and genetic perturbations of Rab27 expression and activity (Fig. 4D). In particular, we compared these staining patterns between control BECs and those treated with Nex-20 + anti-Rab27b antibody (Fig. 4, E and F). We also examined BECs isolated from dHET and dKO mice (fig. S22). We observed that the untreated, vehicle-treated, and dHET control BECs exhibited predicted alignment of ZO-1 staining with membrane-associated CD31 fluorescence and a well-defined network of F-actin filaments. In contrast, ZO-1 expression was markedly reduced and dissociated from CD31 in BECs treated with Nex-20 + anti-Rab27b antibody and in dKO BECs, in which the ZO-1 signal accumulated in the perinuclear space instead of plasma membrane (Fig. 4F and fig. S22). This is of interest as ZO-1 relocation to the nuclear and cytoplasmic regions of the cell was described in settings, where TJs were aberrant (*60*–*62*). Notably, the F-actin expression was also reduced in ECs upon Rab27 targeting, and the actin filaments appeared disorganized (Fig. 4F and fig. S22) in accordance with previous reports (*34*).

To extend these in vitro findings, we stained brain tumor sections for ZO-1 and Cldn5, another tight junctional protein. As in cultured ECs, tumor sections from dKO mice exhibited visibly misaligned expression of ZO-1 with the membrane staining for CD31, which was at variance with intact alignment of these signals in dHET controls (Fig. 4, G and H, and fig. S23). These results were statistically significant, as evident from weighted colocalization analysis (Fig. 4I). Notably, as in the case of ZO-1, the expression of Cldn5 was also markedly reduced in TECs of Rab27-deficient mice (Fig. 4, J and K, and fig. S23).

### Rab27 deficiency compromises brain endothelial barrier function leading to immune cell extravasation into the tumor microenvironment

**Fig. 5.**
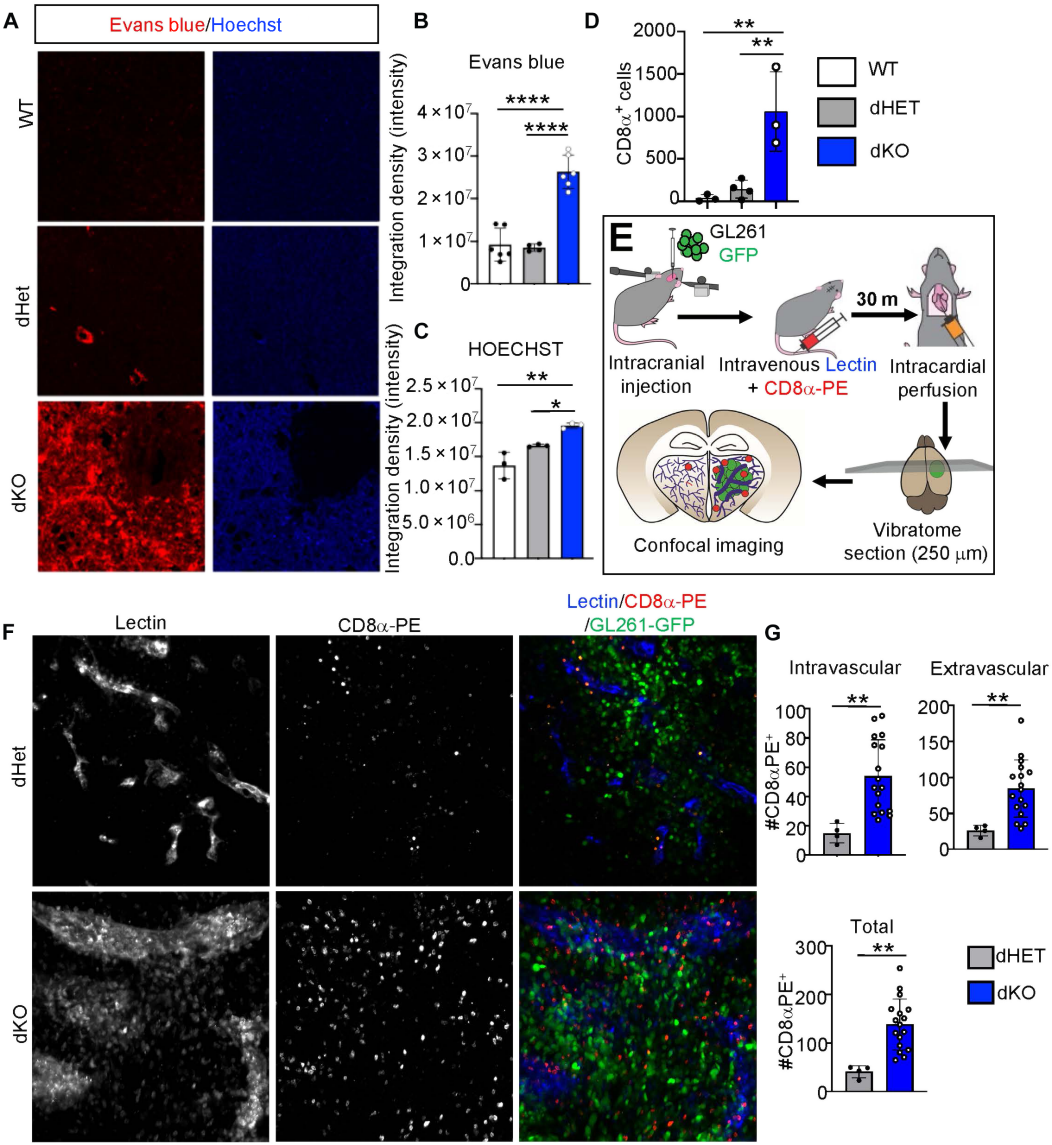
Disruption of Rab27 compromises vascular barrier function in brain tumor microenvironment. (**A**) Entry into brain parenchymal of Evans bue/Hoechst dye cocktail as a function of differential vascular permeability in WT (top), dHET (middle), and dKO (bottom) mice harboring GL261 intracranial tumors. (**B**) Quantification of the intensity of Evans blue tissue containment as measured using ImageJ package. (**C**) Quantification of the intensity of Hoechst, as measured by ImageJ. (**D**) Quantification of CD8α^+^ cell infiltration into brain tumors in WT, dHET, or dKO mice. (**E**) Schematic of the experimental design for testing vascular permeability and T cell extravasation into GL261 intracranial tumors. (**F**) Visualization of blood vessels (lectin, blue), T cells (anti-CD8α–PE, red), and cancer cells (GL261-GFP, green) in tumors of dHET (top) and dKO (bottom) mice using 250-μm-thick vibratome sections. (**G**) Quantification of CD8α^+^ cells located intravascularly (top-left) and in the extravascular tumor parenchyma (top-right) and the total number of CD8α^+^ cells in both compartments (bottom) counted manually; dHET mice (gray); dKO mice (blue). BECs, primary BECs; dHET, Rab27a/b double heterozygote; dKO, Rab27 double knockout. Images were generated using confocal microscopy at 63× and processed through maximum intensity projection to combine the z stacks; *P < 0.05, **P < 0.01, and ****P < 0.0001.

We reasoned that the aforementioned defects in the formation of endothelial TJs and the associated vascular dysmorphia in brain tumor blood vessels of Rab27-deficient mice may be indicative of compromised vascular barrier function and permeability. To test this hypothesis, we used a cocktail of Hoechst and Evans blue dyes, which were injected intravenously at end point and served as tracers of fluid extravasation from the vascular lumen into tumor parenchyma. We observed that the brain tumor vasculature of dKO mice was markedly more permeable to circulating dyes relative to Rab27-proficient (WT and dHET) controls (Fig. 5, A to C). This was a tumor-dependent feature, as in tumor-free mice, Rab27 deficiency resulted in somewhat less (not more) pronounced dye extravasation (fig. S24, A to D). This could be attributed, at least in part, to the low microvascular density observed in the tumor-free dKO mice (Fig. 1, C to F) in parallel with reduction in the TJ proteins in normal BECs (fig. S17). While the mechanisms enabling formation of sparce but functional vascular networks in this setting remain unclear, Rab27-deficient blood vessels retain the ability to respond to angiogenic cues, albeit with considerable delay. This can be inferred from our observation that after 7 days in culture, the magnitude of endothelial outgrowth from explanted aortic rings of Rab27 dKO mice is markedly lower (but not absent) relative to that of their control counterparts and becomes comparable to that of 7-day controls only on day 14 (fig. S24, E to H).

Increased vascular permeability, alteration in the structure of TJs, and down-regulation of leukocyte transendothelial migration pathways in the dKO TEC proteome (fig. S18A) prompted us to investigate whether in Rab27-deficient mice tumor blood vessels are also more prone to extravasation of immune cells into the normally cold tumor microenvironment. Strikingly, staining for a pan-leukocytic marker, CD45^+^, as well as markers of NK cells (NCR1^+^) and cytotoxic effector T cells (CD8α^+^) revealed a marked increase in the infiltration of these cells into microenvironments of GL261 (figs. S25, A to H and S29, A to C, and Fig. 5D) and E0771 (fig. S26) brain tumors in dKO mice, relative to WT or dHET controls. As expected, there was no difference in the myeloid/phagocyte population (*63*) in brain tumors of dKO mice relative to controls (fig. S25, I to L). The differences in lymphoid infiltration between Rab27-proficient and Rab27-deficient mice were especially pronounced at the tumor periphery but remained robust within the tumor core (fig. S27).

In this context, T cells are of particular interest as possible prime effectors of anticancer immunity. To exclude the possibility that the increased influx of CD8α^+^ T cells into the tumor microenvironment of dKO mice was due to increased CD8α^+^ T cell numbers in the circulation, we conducted fluorescence-activated cell sorting (FACS) analysis on their content in peripheral blood. Gating for CD3^+^, CD4^+^, and CD8α^+^ T cells revealed no global differences based on the Rab27 status (fig. S28).

We next investigated whether the elevated CD8α^+^ T cell infiltration into the tumor mass in dKO mice was directly linked to the reduced vascular brain barrier function. To accomplish this, WT, dHET, and dKO mice were intracranially inoculated with GL261 cells, and upon tumor formation, the mice were injected intravenously with a cocktail of fluorescent *Lycopersicon* lectin (far red) and anti-CD8α antibody [phycoerythrin (PE)] to simultaneously highlight blood vessels and T cells, respectively (Fig. 5E). The analysis of perfusion-fixed brain tissues demonstrated the expected containment of the lectin fluorescence at the vascular wall in tumors of Rab27-proficient mice, while both the lectin and CD8α-PE staining were found to efficiently extravasate deep into the tumor microenvironment of Rab27-deficient animals (Fig. 5F and fig. S29D). In this case, CD8α-PE^+^ T cells could be readily observed, both within enlarged tumor blood vessels and extravascularly in brain tumors of dKO mice (Fig. 5G and fig. S29E), whereas they were scarcely detectable in either compartment of the control counterparts. Collectively, these observations suggest that the tumor vascular wall in the dKO mice was permeable to both fluorescent dyes (plasma) and blood cells, allowing the massive entry of CD8α^+^ T cells into the brain tumor milieu.

### Targeting claudin5 enables immune cell infiltration into brain tumor microenvironment

**Fig. 6.**
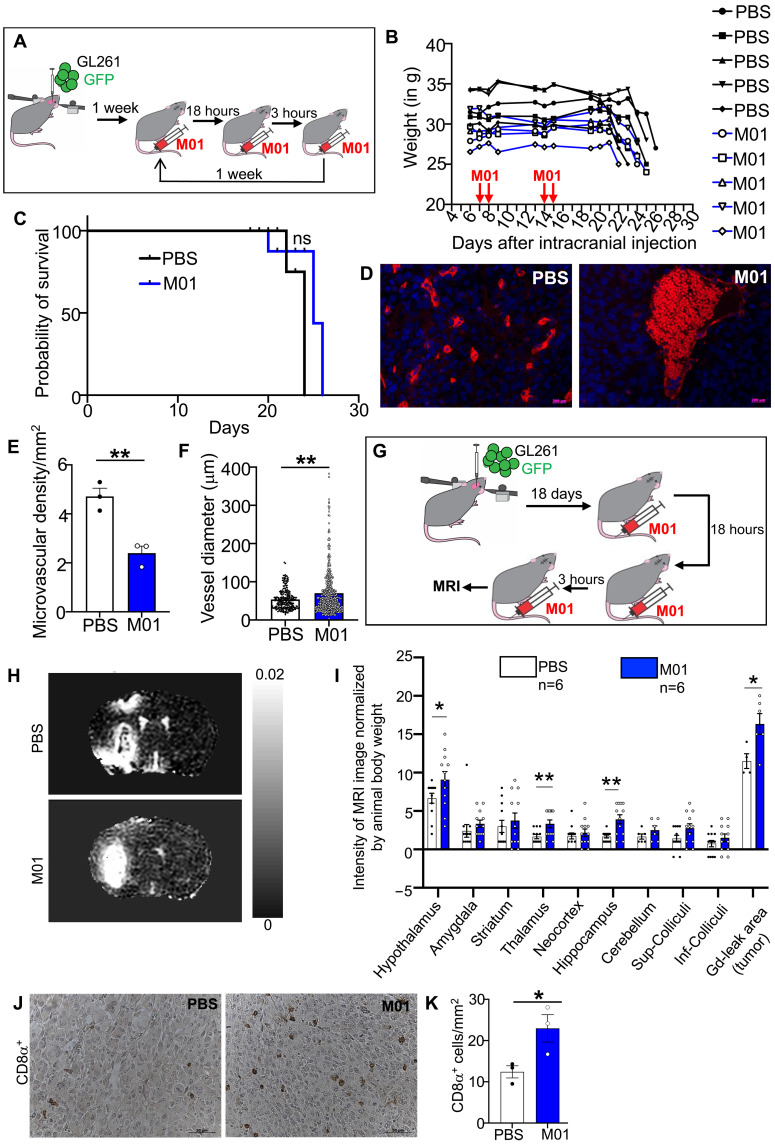
Impact of targeting TJ machinery (Cldn5) in ECs resembles the consequences of Rab27 deficiency. (**A**) Schematic representation of in vivo experiment to study effects of Cldn5 inhibitor, M01 on the brain tumor vasculature. (**B**) M01 safety profile; weights of C57bl/6 mice during the course of M01 administrations indicate no systemic toxicity in the animals exposed to the adopted treatment regimen. (**C**) Kaplan-Meier curves documenting the absence of notable prosurvival effects of M01 alone (versus PBS) in GL261 brain tumor bearing mice. (**D**) Immunostaining for CD31 in sections of GL261 brain tumors following treatment with M01 or PBS demonstrates the emergence of larger vessel sizes in the M01 group. (**E** and **F**) Quantification of microvascular density (E) and vessel diameter (F) in GL261 brain tumor–bearing mice treated with PBS (white) or M01 (blue). (**G**) Schematic representation of M01 administration scheme before subjecting the mice to magnetic resonance imaging (MRI) for vascular permeability assessment. (**H**) Representative MRI scans of the brains of mice treated with PBS (top) and M01 (bottom) with GADOVIST as a contrast agent. (**I**) Quantification of the different regions of the brains after M01 administration shows leaky vasculature in the M01 group relative to controls. (**J**) Immunohistochemistry for CD8α in brain tumor tissues of animals treated with PBS or M01. (**K**) Quantification of CD8α numbers per tumor area in animals treated with PBS or M01. *P < 0.05 and **P < 0.01.

Because several TJ pathway proteins (Cldn5, ZO-1, and ZO-2) were down-regulated in Rab27-deficient TECs in concert with increased permeability and leukocyte infiltration in brain tumors of dKO mice, we reasoned that these proteins could act as effectors of Rab 27. To ascertain whether this is the case, we pharmacologically targeted Cldn5, the central and EC-specific TJ protein, to compare to the consequences of Rab27 deficiency. To this end, we used Cldn5 inhibitor (M01), which has been shown to open BBB in a tumor setting (*64*, *65*). BECs were isolated and treated with M01 or its control derivatives, M01A and M01B, resulting in down-regulation of Cldn5 (fig. S30). Next, we injected nontoxic doses of M01 into mice bearing GL261 brain tumors and examined the impact on tumor progression and vascularity (Fig. 6, A and B). While we observed no survival benefit following M01 treatment, as might be expected for these rapidly growing tumors (Fig. 6C), the vascular morphology markedly changed, as illustrated by lower microvascular density and emergence of relatively larger blood vessels relative to controls (Fig. 6, D to F). To test whether blocking Cldn5 would open BBB to plasma, we administered M01 in GL261 tumor-bearing Rab27-proficient mice followed by contrast enhanced magnetic resonance imaging (MRI; Fig. 6G). Cldn5 inhibition lead to an increase in vascular permeability to gadolinium across the brain (Fig. 6, H and I), with a noticeable increase in CD8α^+^ cell influx into the tumor microenvironment (Fig. 6, J and K).

Thus, the inhibition of Cdn5 leads to a similar (albeit potentially less cancer-specific) consequences as the loss of Rab27a/b. This observation enforces the role of these proteins in pathways regulating vascular TJs and endothelial morphogenesis, suggesting that these mechanisms could be targeted for therapeutic purposes.

### Targeting Rab27a/b-dependent vascular barrier pathway enables adoptive immunotherapy in syngeneic brain tumors

The enhanced infiltration of CD8α^+^ T cells into the tumor microenvironment in Rab27a/b-deficient mice does not mitigate tumor progression or increase animal survival relative to tumor-bearing controls (WT and dHET; Fig. 2B). This is expected, as systemic loss of Rab27a/b expression would affect cytotoxic T cells impairing their ability to secrete granzyme A and hexosaminidase and to efficiently kill their targets (*42*, *66*, *67*). However, this systemic immune suppression (not uncommon in patients with cancer) could be overcome by adoptive T cell therapy. To explore this possibility, we used a modified ovalbumin (OVA)/OT-1 model system, whereby GL261 GBM cells were engineered to express OVA, which rendered them susceptible to recognition and killing by OVA antigen-specific CD8α^+^ T cells derived from OT-1 transgenic mice (*68*). Spleens of OT-1 mice contain >90% effector CD8α^+^ T cells, relative to only ~21% in C57bl/6 mice (*69*). This makes OT-1 donor mice an efficient source of anti-OVA effector cells.

**Fig. 7.**
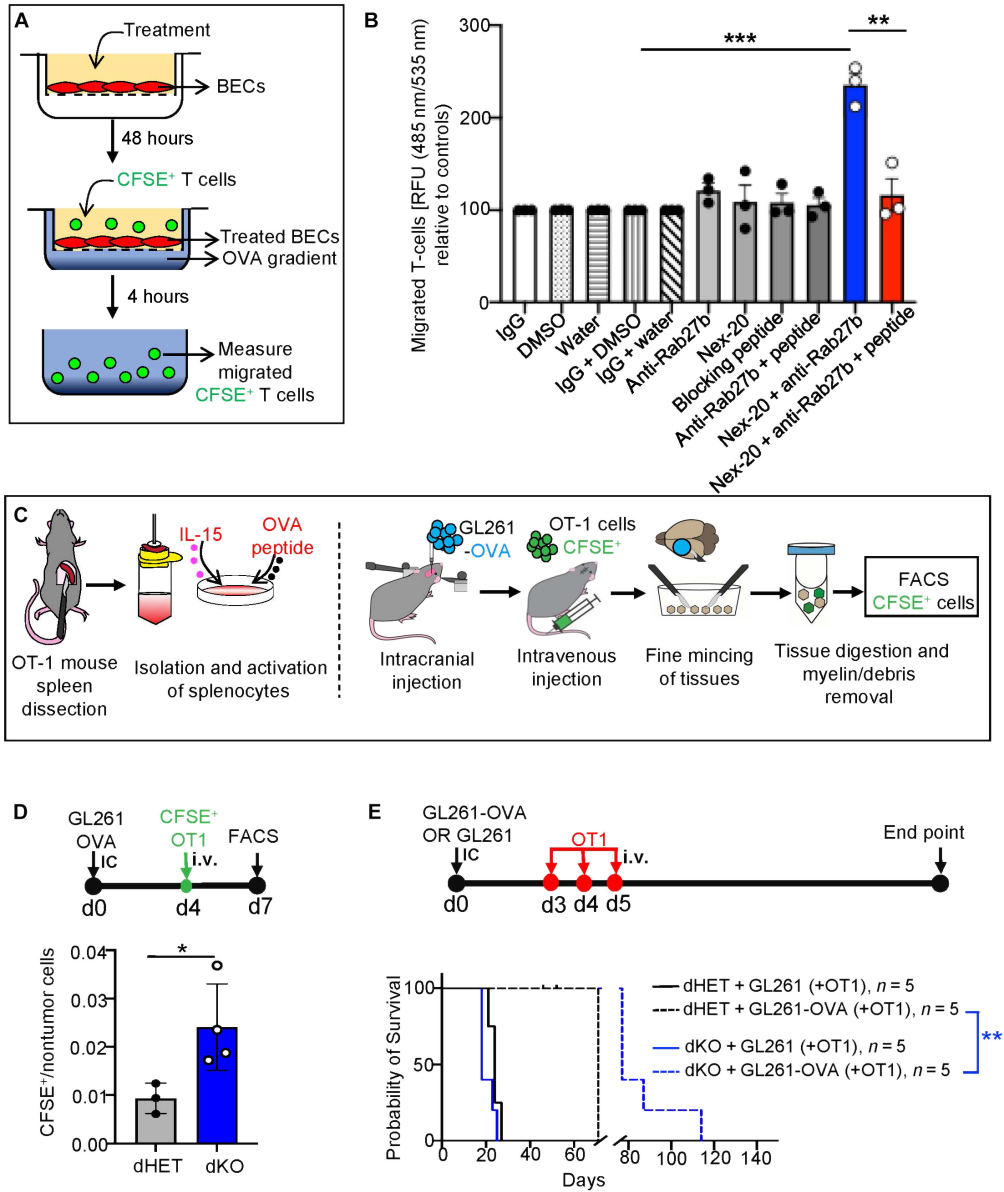
Rab27 inhibition enables adoptive T cell immunotherapy of mouse brain tumors. (**A**) Schematic depicting the experimental design involving CFSE-labeled, OT-1 T cells and their infiltration across the EC barrier in vitro. (**B**) BECs treated with Nex-20 (1 μM), anti-Rab27b antibody (27 μg/ml), and Rab27b blocking peptide (25 μg/ml) mimic the consequences of Rab27 deficiency. (**C**) Schematic of adoptive T cell therapy experiments. (**D**) CFSE-labeled effector OT-1 T cell infiltration into GL261-OVA tumor microenvironment in mice with indicated genotypes; top: experimental design; bottom: the content of CFSE^+^ T cells migrating into brain tumors of dKO and dHET mice as measured by FACS. (**E**) Long-term effects of effector OT-1 T cell systemic adoptive immunotherapy in dHET or dKO mice baring GL261 or GL261-OVA brain tumors. OT-1 T cell therapy had no impact on GL261 tumors but prolonged survival of mice with GL261-OVA tumors in both dHET and dKO mice. The effect of OT1 T cell systemic therapy in dKO mice harboring GL261-OVA significantly increased mouse survival relative to dHET counterparts. BECs, primary BECs; dHET, Rab27a/b double heterozygote; dKO, Rab27 double knockout; RFU, relative fluorescence units. *P < 0.05, **P < 0.01, ***P < 0.001, and ****P < 0.0001.

We first chose to establish whether exogenous T cell passage through endothelial monolayer is directly dependent on endothelial Rab27a/b. To this end, BECs were cultured to confluence in transwell chambers following treatment with either vehicle (DMSO and IgG) or Rab27a/b inhibitors (Nex-20 and anti-Rab27b antibody; Fig. 7A). The cells were then overlayed with carboxyfluorescein diacetate succinimidyl ester (CFSE)–labeled activated OT-1 effector T cells in the top chamber, and the OVA peptide chemoattractant was added to the bottom chamber (Fig. 7B). In keeping with prior observations, we recorded higher numbers of CFSE^+^ T cells migrating through BEC monolayer into the bottom chamber after 4 hours of incubation in the presence of the Nex-20 + anti-Rab27b antibody cocktail relative to control (DMSO + IgG) conditions (Fig. 7B). This effect was rescued (reversed) by using a blocking peptide against anti-Rab27b antibody in conjunction with a combination of this antibody and Nex-20 (Fig. 7B and fig. S31A). Moreover, even a partial genetic silencing of Rab27b (short hairpin RNA) in combination with Nex-20 (fig. S31, B to D), and M01 (fig. S31E) also yielded higher numbers of CFSE^+^ T cells migrating through the endothelial monolayer. These observations suggest that the attenuation of Rab27 activity in BECs is sufficient to compromise their role as a barrier for leukocyte transmigration.

We then surmised that Rab27a/b-dependent changes in the brain tumor vasculature may translate into tumor infiltration with systemically administered anti-OVA/OT-1 effectors to trigger a meaningful antitumor immunity. To test this possibility, dHET and dKO mice were first orthotopically inoculated with GL261 cells expressing OVA antigen (GL261-OVA), followed by intravenous injections of CFSE-labeled, activated OT-1 effector T cells (CFSE-OT-1) (Fig. 7C). Encouragingly, FACS analysis of dissociated mouse brains 3 days post–OT-1 T cell injection revealed appreciable numbers of these CFSE^+^ cells retained in the tissue (Fig. 7D). As expected, we observed a higher number of CFSE-labeled cells in the dKO tumor microenvironment relative to that of dHET controls (Fig. 7D).

The increased infiltration of exogenous OT-1 cells into the brain tumor microenvironment of the Rab27-deficient mice would be expected to exert a prosurvival benefit due to antitumor immune reactivity. To test this prediction, we injected either GL261 (nonantigenic) or GL261-OVA (antigenic) cells intracranially into either dHET or dKO mice, followed by three consecutive intravenous boluses of OT-1 effector cells (Fig. 7, C and E). This treatment induced an appreciable increase in symptom-free survival in Rab27-deficient mice harboring intracranial GL261-OVA tumors relative to their effects against GL261 tumors (Fig. 7E). Moreover, the effect of OT-1 cells was also more pronounced in Rab27a/b-deficient mice with T cell permeable vasculature compared to tumor-bearing dHET littermates (Fig. 7E). These results suggest that vascular dysmorphia and loss of endothelial barrier function due to Rab27a/b deficiency promotes effectiveness of adoptive antitumor immunotherapy.

While encouraging, our results involve syngeneic mouse tumor models that do not fully capture the complexity of the corresponding human tumors. For example, transcriptomes of GL261 and E0771 cells exhibit relatively weak similarity to the corresponding types and subtypes of human cancers, with stronger overlaps with human tumors exhibiting mixed molecular characteristics (figs. S32 and S33). While this is a common challenge of immunotherapy studies, it is noteworthy that there are several emerging links between Rab27a/b and human brain tumors. First, we observed that Rab27a/b is expressed in the human brain vasculature (fig. S34, A to C) and in human brain cancers, including GBM, at mRNA (fig. S34D) and protein levels (figs. S35 to S37). Second, Rab27a/b mRNA expression data extracted from The Cancer Genome Atlas (TCGA) suggest that in GBM, there is a longer progression free survival (fig. S38) in individuals with lower Rab27 levels, albeit without significant impact on the overall survival. Thus, the expression of Rab27 appears to be relevant in settings of human brain tumors, even if further studies are required to understand their roles in specific disease contexts.

## DISCUSSION

Our results suggest a unique and critical role for Rab27a/b proteins in the control of the vascular integrity and immune barrier function in the brain. Vascular barrier exerts a profound and consequential influence over the brain tumor microenvironment, immunoregulation, and therapeutic responses in cancer. The involvement of Rab27a/b proteins in these processes is of interest for at least two major reasons. Biologically, Rab27 proteins lie at the crossroads of multiple regulatory pathways linking intracellular signaling compartments, cytoskeleton, endosome, plasma membrane, and cell-cell communication machinery (*34*). These pathways integrating multiple cellular responses required for vascular morphogenesis and function remain poorly understood, with our study shedding light on some of their unique aspects and implications. Therapeutically, Rab proteins and their interactors could serve as targets of adjunct interventions modifying tumor microenvironment, as exemplified by our results with adoptive T cell transfer.

Rab27-deficient mice are viable but exhibit impaired exocytosis in several cellular compartments such as neutrophils (*70*), mast cells (*43*), and platelets (*40*), albeit with different mechanisms being involved (*40*, *43*, *70*, *71*). Rab27 proteins are ubiquitously expressed in various tissues, such as the spleen, pancreas, lung, hematopoietic lineage, eye, gastrointestinal tract, and, to a lesser extent, the brain, heart, liver, muscle, and testis (*39*, *40*, *72*). They have already emerged in diverse physiological and pathological settings, such as cancer progression and metastasis (*73*, *74*), skin pigmentation (*34*), metabolism (*75*), neurodegenerative diseases (*76*), lung physiology (*77*), secretory apparatus including biogenesis of EVs (*36*), regulation of hemostasis and platelet function (*40*), angiogenesis (*33*), and immunity (*34*). Our study extends this repertoire pointing to the hitherto unappreciated role of Rab27 proteins in blood vessel wall integrity in the brain. The obliteration of Rab27 expression alters both normal and glioma-related brain vasculature and, in the latter case, beyond the notion of disrupted BBB, with a marked increase in immune cell infiltration. Our observations suggest that in Rab27-deficient mice, the down-regulated and altered distribution of endothelial TJ proteins (ZO-1 and Cldn5) and disorganized F-actin coincides with, and likely contributes to, the formation of dysmorphic, hyperpermeable, and dilated tumor-associated vasculature, with altered endothelial landscapes and permissive of paracellular extravasation by anticancer T cells (fig. S39).

The detailed nature of endothelial phenotype(s) associated with reduced barrier function in the absence of Rab27 expression remains to be elucidated in greater detail. It is possible that pathways driven by Rab27, its molecular interactors, and effectors, such as exophilins, munc13-4, SPIRE, and MyRIP may directly affect TJ assembly through mechanisms yet to be fully mapped. Our scRNAseq study points to a diversity in Rab27-dependent molecular shifts among heterogeneous EC subpopulations in the normal brain (BEC) and in those forming the tumor microcirculation (TEC). Notably, among tumor-associated perturbations in EC landscapes, the Rab27 deficiency causes a notable loss of venous-type cells with prosurvival signature, a trait that may affect functional aspects and integrity of the neovasculature (Fig. 3C). It should be noted that scRNAseq of ECs sometimes deviate from established molecular markers of vascular cell populations. This may, at least in part, result from zonation of the vascular system in the brain (*78*) or from the influence of the tumor microenvironment that may trigger phenotypic and molecular anomalies (*38*).

It is noteworthy that the hyperpermeable and dysmorphic vascular phenotype in Rab27-deficient mice is tumor specific. In this regard, our results suggest that angiogenesis, and vascular morphogenesis are delayed, but not abolished in the absence of Rab27, as suggested by results of aortic ring ex vivo assays and by the scarce but functional vasculature of adult mouse brains in vivo. Thus, it could be suggested that persistent endothelial stimulation in cancer or in vitro treatment with growth promoting factors could be essential to expose the defect of Rab27-deficient ECs in assembling functional vascular structures.

While isolated ECs exhibit impaired morphogenesis in the presence of Rab27 inhibitors, it is currently unclear whether their altered assembly in vivo, especially in the context of brain tumors, is driven solely by cell-intrinsic effects. It is conceivable that the vascular dysmorphia observed in brain tumors of Rab27-deficient mice could be, to some degree, a function of perturbations in the secretory pathways and altered reciprocal interactions involving perivascular cellular populations, such as pericytes (*34*), or other cell types in the tumor microenvironment (fig. S37). This aspect remains to be further examined. However, we suggest that the role of Rab27 in the control of immune cell extravasation can largely be attributed to ECs themselves, as indicated by the ability of Rab27 inhibitors to compromise the barrier function of isolated endothelial monolayers in vitro, allowing T cell transmigration.

Our study focuses on biological aspects of the brain vasculature and uses syngeneic tumor models to study the related interactions with the immune cell compartment. This is an important step in considering the related complexities of tumor-vascular-immune interactions in the realm of human disease (*7*, *27*, *79*). It should be kept in mind that in clinical settings, factors related to molecular underpinnings and driver events operative in specific human brain tumors, either primary or metastatic (*7*, *80*), as well as diverse neovascularization mechanisms (*81*), microenvironmental stresses, therapies, and other factors (*27*) may all profoundly affect vascular interactions. While our analysis suggests the expression of Rab27 in human brain tumors and in human cerebral vasculature, the role of endothelial Rab27 in regulating immune cell infiltration in specific human brain cancers merits more contextual follow-up analysis.

Overall, our study suggests that Rab27 proteins control tight junctional apparatus of the brain microvasculature, including claudin 5, and that disruption of this mechanism enables marked influx of immune cells into the tumor microenvironment. These considerations are of interest in the context of therapeutic strategies involving modulation of the tumor vascular compartment to enhance the effects of anticancer immunotherapy (*26*), especially in aggressive primary and metastatic brain tumors. This notion is exemplified by GBM, a lethal brain cancer, which remains poorly responsive to systemic forms of immune intervention in the face of immunologically cold tumor microenvironment, systemic immunosuppression, and paucity of immune effectors able to pass the vascular barrier into the tumor parenchyma (*82*). Rab27 proteins and their related pathways are pharmacologically targetable (*45*) and nonessential for survival in mice (*40*). Our study documents the biological feasibility of targeting Rab27 or claudin 5 to increase T cell influx into the brain, as a proof of concept requiring further validation and assessment of safety. Still, it is tempting to speculate that modulation of the Rab27-dependent vascular wall permeability could serve as an adjunctive intervention strategy to improve the effects of immunotherapies in brain cancer.

## MATERIALS AND METHODS

### Cell culture

Murine glioma cell line GL261 (a gift from X. Breakefield), breast cancer cell line, E0771 [American Type Culture Collection (ATCC)], and bEND.3 cells (ATCC) were cultured using Dulbecco’s modified Eagle’s medium (DMEM) high-glucose medium supplemented with 10% fetal bovine serum (FBS) and 1X penicillin/streptomycin (PenStrep) (Life Technologies). Mouse primary brain microvascular ECs (#C57-6023, Cell Biologics Inc.) were cultured in cell culture dishes (#150350, Thermo Fisher Scientific) coated with gelatin (#6950CE, Cell Biologics Inc.) in complete mouse EC medium (#M1168, Cell Biologics Inc.). The mouse primary brain vascular ECs (BECs) were regularly stained with anti–CD31–allophycocyanin (APC) antibody (#551262, BD Biosciences) and subjected to FACS analysis to ensure purity. BECs were treated with DMSO (#D2650, Sigma-Aldrich) or Nex-20 (SML1919, Sigma-Aldrich) at several concentrations (0.5, 1, 2, 5, 10, and 20 μM) to determine a maximal nontoxic dose. Likewise, BECs were treated with either IgG (I4131, Sigma-Aldrich) or Rab27b antibody (#ABIN6272904, Antibodies online) at several concentrations (1.35, 2.7, 5.4, and 10.08 μg/ml) to determine the maximum dose not toxic to the cells, which was established at 2.7 μg/ml for both Rab27b antibody and IgG. Likewise, Rab27b antibody blocking peptide (#DF12060-BP, Affinity Biosciences) concentrations were tested (2.5, 5, 10, and 20 μg/ml), and 2.5 μg/ml was chosen, as it was the closest to the concentration of Rab27b antibody used. M01 (#502995-31-5, Enamine), M01A (#206-864-7, Enamine), and M01B (#GN161786, Enamine) were tested for toxicity (MTS assays), and maximum nontoxic concentrations of all reagents were used throughout further experiments, which was 10 μM for BECs and 50 μM for bEND.3 cells. OT-1 T cells were obtained from the spleens of OT-1 mice. Briefly, spleens of OT-1 mice were harvested into cold RPMI media (#350-007-CL, Wisent Inc.), followed by passage through a 70-μm cell strainer (#22-363-548, Thermo Fisher Scientific) into 20 ml of ice-cold media using a 1-ml syringe plunger. Next, the cells were centrifuged at 1300 rpm for 5 min, followed by resuspending the pellet in 2.5 ml of ammonium-chloride-potassium (ACK) RBC lysing buffer (#A1049201, Thermo Fisher Scientific) at room temperature (RT) for 4 min. The suspension was diluted using 30 ml of sterile phosphate-buffered saline (PBS) and centrifuged at 1300 rpm for 5 min to collect the lysis-resistant nucleated cells, followed by their washing with 20 ml of PBS. The cells were resuspended, counted, and cultured in RPMI media containing 10% FBS, 1% penicillin/streptomycin, 50 nM β-mercaptoethanol, and 1% Hepes, along with mouse IL-15 (20 ng/ml; #8080, STEMCELL Technologies) and 0.1 nM OVA peptide (#S795, Sigma-Aldrich) to activate the T cells. T cells were then washed and placed in new cell culture dishes using fresh complete RPMI media. This step was repeated for three consecutive days before using the activated T cells for in vitro or in vivo assays, such as adoptive T cell transfer therapy. All cells were cultured in a 37°C incubator with 5% CO_2_.

### Plasmid constructs and expression vectors

GL261 glioma cells were transduced with GFP-Luciferase (GFP-Luc) construct, which was cloned into a pSMAL vector modified from the MA1 lentiviral vector to have a Gateway cassette and SFFV promoter (*83*, *84*) with luciferase gene cloned from pGL4.51(luc2/CMV/Neo) (E1320 Promega) (obtained from K. Eppert, McGill University). GL261 cells were transduced with BFP-OVA lentiviral construct (clone #135074, Addgene). Primary mouse BECs were transduced with SV40-large T antigen–mCherry lentiviral construct to immortalize them (clone #58993, Addgene). GFP^+^, BFP^+^, and mCherry^+^ cells were isolated using the BD FACSAria Fusion cell sorter.

### Lentivirus production

The day before transfection, 4.5 × 10^6^ 293 T cells were seeded onto 100-mm dish (9 ml). On the day of transfection, the plasmid mixture of 6 μg of VSVG (clone #8454 Addgene), 6 μg of Pax2 (clone #12260, Addgene), and 6 μg of the construct of interest, such as BFP-OVA, was added along with X-TremeGene9 DNA transfection reagent (#XTG9-RO, Roche). The 293 cells were cultured in the absence of PenStrep to facilitate efficient transfection for the first 24 hours. The medium was replaced on the next day with DMEM, 10% FBS, and 1X PenStrep, and the cells were cultured for additional 48 hours. The medium was then harvested and centrifuged at 500*g* for 10 min. A total of 1 volume of Lenti-X Concentrator (#631231, Takara) was added to 3 volumes of clarified supernatant, and the resulting solution was mixed gently by inversion followed by incubation at 4°C for 30 min or overnight. The sample was centrifuged at 1500*g* for 45 min at 4°C. The supernatant was disposed of, and the pellet of lentiviral particles was resuspended in 1/10th to 1/100th of original volume using DMEM and stored at −80°C until used.

### Antibody labeling

Rab27b antibody was labeled using the Thermo Fisher Invitrogen Alexa Fluor 488 microscale protein labeling kit #A30006, as per the manufacturer’s protocol. Briefly, sodium bicarbonate was added to 20 μg of Rab27b antibody, followed by incubating the antibody mix with the reactive dye solution for 15 min. The antibody conjugate was purified using the resin filled spin filter and centrifuged at 16,000*g* for 1 min.

### Western blotting and proteomic analysis

Total cell proteins were isolated using radioimmunoprecipitation assay (RIPA) lysis buffer (10 mM tris at pH 8.0, 1 mM EDTA, 1% Triton X-100, 0.1% sodium deoxycholate, 0.1% SDS, and 140 mM NaCl) containing protease inhibitor (Roche, Mississauga, ON, Canada). After incubation on ice for 30 min, the lysates were centrifuged at 15,000*g* for 5 min at 4°C. Protein concentrations were assessed using the Pierce Micro BCA Protein Assay (Thermo Fisher Scientific, Rockford, IL, USA). Using 10% SDS–polyacrylamide gel electrophoresis, lysates were resolved before transfer onto polyvinylidene difluoride membranes (Bio-Rad, Mississauga, ON, Canada), performed at 100 V for 1 hour at 4°C. Membranes were blocked for 1 hour in 5% skim milk and probed with primary antibodies followed by the appropriate horseradish peroxidase (HRP)–conjugated secondary anti-mouse (1:500; #170-6516, Bio-Rad) or anti-rabbit (1:500; #7074S, Cell Signaling Tehcnology) antibodies. Chemiluminescence (GE Healthcare) was visualized using ChemiDoc MP system (Bio-Rad). Primary antibodies used included the following: mouse anti-Rab27a (#66058-1-IG, Proteintech), rabbit anti-Rab27b (#13412-1-AP, Proteintech), and mouse anti–β-actin (1:1000; #A1978, Sigma-Aldrich).

### Immunostaining

All brain tissues were preserved in 4% paraformaldehyde (PFA) after dissections. Tissues were passed through the tissue processor (Leica TP 1050) and embedded in paraffin blocks, from which 5-μm-thick sections were cut using a microtome (American Optical). Tissues were dewaxed and rehydrated in a series of 5-min steps involving xylene and 95 to >50% ethanol. Tissues were further processed by heating at 95°C for 15 min in unmasking solution (#H3300, VectorLabs) for antigen retrieval. The sections were then incubated with blocking solution for 1 hour in RT, followed by incubation with primary antibody overnight at 4°C. The tissues were washed for 5 min three times in PBS and then incubated with the respective fluorescent secondary antibodies for 1 hour at RT. The tissues were washed three times in PBS for 10 min each followed by mounting them with mounting solution VECTASHIELD HardSet (containing 4′,6-diamidino-2-phenylindole) to seal the slides with coverslip before viewing.

For DAB immunohistochemical staining, slides were dewaxed and processed through ethanol series with subsequent washing with PBS (pH 7.4). Endogenous peroxidases were quenched by placing the slides in a mixture of 49% methanol:49% PBS:2% H_2_O_2_. Brain sections were blocked with normal serum compatible with the secondary antibody species and then incubated with the primary antibodies overnight at 4°C in a humidified chamber. The following day, slides were incubated with the appropriate HRP-conjugated secondary antibodies (Vector rabbit ABC kit, Vector Laboratories, Burlingame, CA, USA) for 30 min at RT. Signals were detected using DAB substrate kit (Vector). Tissues were then counterstained with hematoxylin, viewed, and microphotographed under the microscope.

For immunofluorescence of cells in vitro, the indicated cells were cultured on glass-bottom eight-chamber slides (#80807, ibidi) until confluency. Next, the cells were fixed with 4% PFA for 30 min on ice followed by three washes using 1× PBS. The cells were incubated with 0.1% Triton X-100 in PBS for 5 min followed by three PBS washes. Cells were blocked with 1% bovine serum albumin (BSA) in PBS for 30 min, followed by incubation with primary antibody at 4°C overnight. Next day, cells were washed three times using 1× PBS and then incubated with the respective secondary antibody for 1 hour at RT. The cells were then incubated with NucBlue (#R37605, Invitrogen) for 5 min and washed three times using 1× PBS before viewing under confocal microscope (Zeiss LSM780-NLO laser scanning with infrared optical parametric oscillator lasers) or a super resolution microscope (Zeiss LSM880 ElyraPS1 laser scanning confocal and super-resolution microscope (SIM and PALM/dSTORM mode).

Primary antibodies/probes used in these studies include the following: goat anti-mouse CD31(#AF3628, R&D Systems), anti-Rab27a (#66058-1-IG, Proteintech), anti-Rab27b (#13412-1-AP, Proteintech), phalloidin iFluor 647 reagent (#ab176759, Abcam), anti–ZO-1 (#33-9100, Thermo Fisher Scientific), anti–Claudin-5 (#35-2500, Invitrogen), anti-CD8α clone D4W2Z (#98941S, Cell Signaling Technology), anti-CD45 (#AF114, R&D Systems), anti-F4/80 clone D2S9R (#700761, Cell Signaling Technology), and anti-mouse NCR1 (#AF2225, R&D Systems). The respective secondary antibodies include the following: goat anti-rabbit IgG Alexa Fluor 488 (#A-11034, Invitrogen), donkey anti-goat IgG Alexa Fluor Plus 594 (#A32758, Invitrogen), donkey anti-rabbit Alexa Fluor 647 (#A31573, Thermo Fisher Scientific), and goat anti-mouse Alexa Fluor 647 (#A32728, Thermo Fisher Scientific).

### Cell growth/survival assay (MTS)

Cell titer 96 (#43580, Promega) was used to measure in vitro cellular metabolic activity as a surrogate of cell growth/viability in the presence of indicated reagents. The treatments included the following: IgG, anti-Rab27b antibody, DMSO, and Nex-20. Briefly, 7 × 10^3^ BECs per well were seeded in 96-well plates in full growth media with varying concentrations of these reagents for 72 hours. The assay was developed as per manufacturer’s protocol, the absorbance at 490 nm was read after normalization, and the readings were regarded as reflective of the viable cell numbers per well.

### Endothelial tube formation assay

To assess morphogenetic ability of ECs 100 μl of Matrigel (#356231, Corning) was added per well of the 96-well cell culture plates. To ensure there were no air bubbles, the plates were centrifuged at 300*g* for 5 min at 4°C. The coated plates were placed at 37°C for 30 min to allow solidification of Matrigel. Subsequently, 100 μl of complete EC culture media containing 1.5 × 10^4^ primary BECs in suspension was added and cultured in Matrigel-coated wells either alone or in the presence of indicated agents such as nonimmune IgG, DMSO, IgG + DMSO, Nex-20, anti-Rab27b antibody, or Nex-20 + Rab27b antibody. The BECs were cultured for 6 to 18 hours before imaging and quantification of the tube formation responses using the angiogenesis plugin on ImageJ.

### Flow cytometry

Blood samples harvested from mice through inferior vena cava into citrate anticoagulated tubes were treated with ACK lysis buffer to deplete RBCs. The samples were then centrifuged at 1500*g*, and the pellets of nucleated cells were resuspended in 1× PBS following with incubation for 1 hour with fluorescently conjugated antibodies, including the following: PerCP-labeled anti-mouse CD8α (#100731, BioLegend), APC-labeled anti-mouse CD3 (#100236, BioLegend), and PE-labeled anti-mouse CD4 (#116005, BioLegend) versus the respective nonimmune IgG controls (BioLegend). BECs were treated with Rab27b-488 4.5 hours before trypsinization. All the cells were washed followed by flow cytometry (BD LSR Fortessa), and data were analyzed using the FlowJo software 10.7.1.

### Mass spectrometry

Samples submitted to proteomics were obtained from the brains of 3-month-old mice with or without GL261 tumors. The ECs were isolated using the protocol published recently (*85*). Briefly, brains were homogenized using collagenase/dispase solution (#11097113001, Millipore/Roche) for 30 min, followed by elimination of myelin and other debris using a brief centrifugation with 22% Percoll solution (#P1644, Sigma-Aldrich). After washing, the cells were incubated with anti–CD31-APC (#551262, BD Pharmagen) and sorted using the BD FACSAria Fusion cell sorter. The sorted cells were collected directly into RIPA lysis buffer, protein content quantified and subjected to mass spectrometry.

Proteomic analysis was performed as previously described (*81*). Briefly, data were collected using the Thermo Orbitrap Fusion mass spectrometer operating at 120,000 resolution (full width at half maximum in MS1) with HCD sequencing (15,000 resolution). Data were then analyzed using Prism 9. The raw data were converted into *.mgf format (Mascot generic format) for searching using the Mascot 2.6.2 search engine (Matrix Science) against Mouse Uniprot dB from 2023. The database search results were loaded onto Scaffold Q + Scaffold_4.9.0 (Proteome Sciences) for statistical treatment and data visualization. The analysis for biological pathways and Gene Ontology terms associated with differentially regulated proteins was performed using DAVID (https://david.ncifcrf.gov) platform.

### Brain tumor generation and analysis

Intracranial injections, permeability assays, CD8a-PE and *Lycopersicon* lectin injections, perfusion, and vibratome sectioning were conducted according to previously described protocols (*81*, *86*). Briefly, orthotopic inoculations were carried out by injecting 2 μl of PBS containing 2.5 × 10^3^ GL261 cells in suspension using a stereotactic frame (Stoelting). The cells were inoculated into the brains of 3-month-old C57bl/6 mice (Charles River Laboratories) or syngeneic strains deficient for Rab27a/b (dKO, a gift from M. Seabra) or their heterozygous Rab27a/b-proficient (dHET) littermates. At end point, the mice were injected intravenously with a dye cocktail containing Hoechst 33342 (10 mg/kg; #62249, Thermo Fisher Scientific) and Evans blue (200 mg/kg; #E2129, Sigma-Aldrich) to allow fluorescent and colorimetric tracking of vascular permeability, respectively. To visualize dye extravasation, tissues were post-fixed in 4% PFA overnight at 4°C followed by incubation in 30% sucrose at 4°C. The tissues were cryopreserved using O.C.T. (#4583, Tissue-Tek) and sectioned at 10 μm using a cryostat (Leica) before imaging the same day under confocal microscope. Alternatively, to capture simultaneously the trans-endothelial lymphocyte passage and the integrity of intratumoral endothelial lining, 30 min before euthanizing, mice were injected intravenously with anti–CD8α-PE (#100708, BioLegend) to highlight T cells in situ and/or with fluorescent *Lycopersicon* lectin (#DL-1178, Vector Laboratories) to label intraluminal endothelial surfaces of perfused blood vessels. Under terminal anesthesia, the animals were perfused using sterile PBS followed by 4% PFA, and immediately following euthanasia, the brains were collected in ice-cold sterile PBS. Brain tissue sections of 150 to 200 μm in thickness were prepared using vibratome (Leica VT 1200 s). The tissues were placed in a μ-Dish 35 mm, high glass bottom dish (#81158, ibidi) and imaged under high-resolution confocal microscopy (Zeiss LSM780 laser scanning confocal microscope).

### Standards for animal studies

All animal studies were conducted in accordance with the guidelines of the Canadian Council of Animal Care and the Animal Utilization Protocols, approved by the Institutional Animal Care Committee at the Research Institute of the McGill University Health Center (RIMUHC) and McGill University. For experiments involving intracranial injections, the clinical end point was based on the first sign of neurological symptoms, such as dehydration, hunched posture, or circling. No decline in well-being was permitted. All mice were maintained at RIMUHC Animal Care Facility, monitored daily, under 12-hour light/12-hour dark cycle and under constant veterinary care.

### Endothelial transmigration assays

For the analysis of immune cell transmigration across endothelial barrier, 5 × 10^4^ BECs were cultured at confluence on the inserts of transwell chambers (#3421, Costar) for 2 days in the presence of complete EC media. Next day, the BEC monolayers were cultured with media containing either DMSO + nonimmune IgG (control) or Nex-20 + anti-Rab27b antibody. On the second day, the inserts were washed with sterile PBS and replaced with 7.5 × 10^5^ activated OT-1 T cells labeled with CFSE (#C34554, Thermo Fisher Scientific). In preparation for this step, the OT-1 cells were incubated with CFSE for 20 min at 37°C resulting in unform fluorescent labelling. The bottom chamber of the transwell dish was filled with 600 μl of media containing DMEM (Invitrogen), 10% FBS, and OVA peptide (2 ng/ml), and the cocultures were incubated for 4 hours at 37°C incubator with 5% CO_2_. Following the incubation, the inserts were removed, and the relative fluorescence unit of OT-1 cells that has migrated into the bottom chamber through the endothelial monolayer was recorded using plate reader (Infinite M200 Pro, Tecan).

### Adoptive T cell therapy

Short-term adoptive cell transfer was calibrated using the GL261 model. Briefly, 5 × 10^5^ GL261-BFP-OVA cells were inoculated intracranially into 3-month-old mice with defined Rab27 status (dHET or dKO). After 4 days, 5 × 10^5^ CFSE-labeled and preactivated OT-1 T cells were injected intravenously into the tail veins of the mice. After 3 days, brains containing incipient tumors were harvested and subjected to FACS analysis to record the number of extravasated CFSE^+^ (green) OT-1 cells amid BFP^+^ (blue) tumor cells. Long-term experiments were performed by injecting 8 × 10^3^ GL261-BFP-OVA (or GL261) cells intracranially into 3-month-old mice followed by three consecutive intravenous (lateral tail vein) injections of activated OT-1 T cells into tumor-bearing mice with defined Rab27 status. We used deescalating protocol of OT-1 cell delivery, in that the first injection consisted of 2 × 10^6^ of these cells, while the second round included 1 × 10^6^ OT-1 cells and 0.5 × 10^6^ cells were injected as the final course. The mice were monitored and euthanized on the first indication of impending neurological symptoms. For M01 experiments, intracranial tumor bearing mice were administered with a triple dose of 2.8 μmol/kg of the drug, or PBS as control, 7 days after GL261 brain tumor implantation.

### Bone marrow chimera

To address the possible contribution of bone marrow–derived cellular populations to the vascular phenotype of Rab27-proficient and Rab27-deficient mice a reciprocal bone marrow chimera experiments were carried out. Briefly, 3-month-old recipient mice were given full body x-ray irradiation of 9Gy using the X-RAD SmART PXi PRECISION X-RAY irradiator. After 3 hours, the recipient mice were repopulated by intravenous injection of 5 × 10^6^ of freshly isolated bone marrow cells per animal. Bone marrow cells were harvested from femurs of 3- to 4-month-old donor mice with indicated Rab27 status. To do so, femurs were cleaned of the muscle and fibrous tissues using sterile scalpels, and the bones were wiped with Kimwipe to remove any remaining soft tissue. The epiphyses of each femur were cut open using sterile scalpel blades, and the bone marrow was flushed out into sterile media. This was accomplished using a 10-ml syringe with 25-gauge needle, through which 5 ml of RPMI media was aspirated into the syringe. The needle was then inserted into one of the ends of the femur, while the other end of the femur was placed into a culture dish with 7 ml of ice-cold RPMI. The RPMI content of the syringe was used to flush carefully the bone marrow out of the femur and into the culture dish. The flushing was repeated several times to ensure maximum bone marrow cell recovery. The cells collected in RPMI were transferred into a conical tube and centrifuged at 600*g* for 5 min. After discarding the supernatant, the pellet was resuspended in ACK lysis buffer and allowed to incubate for 3 min to eliminate RBCs, after which 10 ml of sterile PBS was added, and the tube was recentrifuged at 600*g* for 5 min. The pelleted cells were resuspended in PBS and counted before injection. After ensuring that stable weights were maintained by the chimeric animals, we collected a few drops of blood from the mice to validate the presence of GFP^+^ donor cells in GFP^−^-recipient mice and vice versa using flow cytometry. Following successful validation of the desired bone marrow chimerism, 2.5 × 10^4^ of GL261-GFP-Luc cells were injected intracranially into the chimeric mice, which were monitored for disease progression, and analyzed for vascular patterns.

### Fluorescent RNA in situ hybridization and qRT-PCR

BECs and bEND.3 cells were seeded at 3 × 10^3^ cells per well in eight-chamber wells (#80826, ibidi) and fixed the next day using reagents provided by the ViewRNA Cell Plus Assay Kit (88-19000-99, Thermo Fisher Scientific). The protocol was followed based on manufacturer’s recommendations. Fluorescent RNA probes specific to Rab27a (VB4-3114104-VC) and Rab27b (VB4-3127686-VC) were obtained from Thermo Fisher Scientific. For quantitative reverse transcription polymerase chain reaction (qRT-PCR), the test was performed on mRNA extracted from BECs and TECs, which were isolated from mice by sorting for CD31^+^cells.The RNA was extracted by adding 800 μl of TRIzol to 30 μl or less of cell volume, followed by incubation at RT for 3 min. Next, DNA was sheered using 26-gauge needle, 5 μg of linear polyacrylamide (ambion) was added, and the tube was vortexed, followed by addition of 160 μl of chloroform. The tube was then centrifuged at 12,000*g* for 5 min at 4°C. Aqueous phase was transferred into a new tube, and 400 μl of ice-cold propanol was added followed by vortexing the mix and incubating the tube at −20°C overnight. Tubes were then centrifuged at 12,000*g* for 15 min at 4°C. The RNA pellet was washed with ice-cold 75% ethanol, and the dry pellet was resuspended in 15 μl of ribonuclease-free water. The sequences of primers were as follows: Rab27a forward primer (5′-CTATGGGTTTCCTGCTTCTG-3′), Rab27a reverse primer (5′-TCTGTAGCTGGCTTATCCA-3′), Rab27b forward primer (5′-GCCCTCCAAGACCATCACTATG-3′), Rab27b reverse primer (5′-GCTCCATCTGCTCCTTGTGTGT-3′), glyceraldehyde-3-phosphate dehydrogenase (GAPDH) forward primer (5′-GTTTCCTCGTCCCGTAGACAAA3-′), and GAPDH reverse primer (5′-CTTCCCATTCTCGGCCTTG3-′). cDNA was prepared using high-capacity cDNA reverse transcription kit (# 4368814, Applied biosystems) and SYBR Green Master Mix (#4367659, Applied Biosystems). qRT-PCR was performed using the Roche LightCycler 96 with the 45-cycle amplification program of 95°C for 30 s, 53°C for 30 s, and 72°C for 60s.

### Aortic ring assay

The aortic ring assay was performed as previously described (*87*). Aortas of 4-week-old WT, dHET, and dKO mice were harvested and cultured in growth factor reduced Cultrex BME (3433-010-R1, R&D Systems) matrix polymerized at 37°C. The aortic rings were imaged after 7 and 14 days. The explants were cultured in DMEM/F12 (11320-033, Gibco), 1× PenStrep (LS15140148, Gibco), 2% FBS (080-150, Wisent Inc.), and ECGS (E2759, Sigma-Aldrich).

### Single-cell RNA sequencing

Samples submitted for scRNAseq were obtained from the brains of 3-month-old mice with/without GL261 tumors. The ECs were isolated using the protocol published recently (*85*). The single-cell suspension was treated with ACK lysis buffer to eliminate RBCs (3 ml for 3 min) at RT after which 15 ml of sterile PBS was added before centrifuging the sample at 12,000 rpm for 5 min. The cell pellet was resuspended in 90 μl of buffer (PBS, 0.5% BSA, and 2 mM EDTA), followed by addition of 10 μl of anti-CD31–coated beads (catalog no. 130-097-418, Miltenyi Biotec), and incubated for 15 min on ice. Subsequently, 2 ml of buffer was added to the sample and centrifuged at 300*g* for 10 min. The cell pellet was resuspended in 500 μl of buffer and subjected to the prewashed LS columns (catalog no. 130-042-401, Miltenyi Biotec) on the MidiMACS separator attached to a multistand (catalog no. 130-042-301, Miltenyi Biotec). Afterward, 3 ml of buffer was used to wash the column three times before collecting the bead-conjugated cells in a separate tube using the column plunger. The cells were counted, and only the viable cells were subjected to single droplets and library preparation. The viability of cells was as follows: 91% dKO_TECs, 88% dKO_BECs, 89.6% dHET_TECs, and 87.2% dHET_BECs.

Single-cell cDNA library of ECs isolated from the mouse brain tissues was prepared using the Chromium Next GEM Single Cell 3’ Kit v3.1 (10X Genomics) following the manufacturer’s protocol. Single-cell RNA library was generated using the GemCode Single-Cell Instrument (10X Genomics, Pleasanton, CA, USA). Library preparation for MGI, MGIEasy Universal library conversion, and quality control was used. The sequencing-ready library was purified with SPRIselect, quality-controlled for sized distribution and yield (LabChip GX Perkin Elmer), and quantified using qPCR (KAPA Biosystems Library Quantification Kit for Illumina platforms P/N KK4824). The libraries were then pooled and sequenced on MGI DNBSEQ G-400 and PE100 and aligned to the mouse reference genome mm10 using the Cell Ranger v7.1.0. We used Cell Ranger v3.0.1 (10x Genomics) to demultiplex the raw sequencing reads to FASTQ files and align the reads to mouse reference mm10 to quantify gene counts (UMIs), getting about 45.8 ± 6.8 million read counts (means + SD) in about 8105 ± 2750 cells for the four different conditions. The gene count data have been processed following the Seurat pipeline, and low-quality cells were filtered out by the following criteria: RNA less than 3000 counts, genes less than 500 or more than 7000, and mitochondrial gene fraction more than 15 or 0%. The filtered count data were log-normalized and scaled for downstream analyses. We identified the top 2100 highly variable genes and used 2098 genes excluding two mitochondrial genes for principal components analysis (PCA) calculation. The top 30 PCs were used to identify shared nearest-neighbor–based clusters with the resolution of 1.5 and data UMAP visualization. We then selected EC clusters based on canonical marker expression and filtered out potential endothelial doublets coexpressing sets of canonical markers of other cell types or unique markers of the non-endothelial clusters existing in the dataset. Endothelial clusters were separately analyzed and characterized based on previous studies on murine ECs (*49*, *51*). The normalized data were visualized using the Seurat and dittoSeq packages.

### Magnetic resonance imaging

All mice were imaged using a Bruker BioSpec 70/30 MRI system equipped with a volumetric neuro coil. Anesthesia was induced with 4 to 5% isoflurane and maintained at 2 to 3% isoflurane throughout the procedure while maintaining the body temperature at 37°C and monitoring respiration. Baseline imaging was performed using a 3D T2-weighted fast low-angle shot (FLASH) MRI sequence, with an acquisition time of 8 min and 24 s. After the initial scan, mice were administered a gadolinium-based contrast agent (Gadovist 1.0, Bayer Pharmaceuticals) via tail vein injection at a dose of 3 mmol/kg. Following contrast injection, a second 3D T2 FLASH MRI scan was immediately performed.

Subsequent image processing was conducted using MATLAB to coregister the pre- and post-contrast images. The pre-contrast and post-contrast images were subtracted to enhance the visualization of gadolinium uptake in the vasculature and tumor regions. A mouse brain atlas was overlaid on the subtracted images for anatomical reference. Gadolinium uptake was normalized to body weight, and the contrast enhancement in various brain regions and tumors was quantified using MATLAB and AMIDE software. Data analysis was performed using GraphPad, with SDs calculated for each measurement.

### Data mining for human and mouse brain tumors

The data acquisition and processing of TCGA patient datasets for GBM and breast cancer datasets were obtained from the cBioPortal database for use as control datasets. Specific studies include GBM: GBM TCGA Pub 2013 and Breast Cancer: BRCA MBC Project 2022. Additional data were retrieved from the following Gene Expression Omnibus (GEO) accession numbers: GL261: GSE178371, E0771: GSE244138, GL261_2: GSE214294, and BRAIN: GSE266210. For data processing, since cBioPortal provides data as gene expression counts, raw FASTQ files from GEO were processed for maximized compatibility. This involved generating counts using the RSEM tool and normalizing the data with the NormalizeExpressionLevels_allsampleref.py script. Mouse gene expression counts were mapped to their human orthologs using a custom Python script that used the “mygene” library for annotation. For analysis and visualization, in heatmap, *z* scores were computed from log-transformed normalized expression data. A custom Python script was used for visualization. For PCA, it was performed using Python. Data were normalized and batch-corrected using the Combat algorithm and “sklearn.” Input for PCA was restricted to 150 genes representing three predefined gene signatures. Gene Set Enrichment Analysis (GSEA) was performed for single-sample GSEA (ssGSEA) using Python and the “gseapy” library. Gene sets of GBM were based on the three classification groups based on the 50 gene signatures for classical, mesenchymal, and proneural tumors.

### Statistical analysis

One-way analysis of variance (ANOVA) using Tukey’s to correct for multiple comparisons was used for comparison of more than two groups of data, while standard unbiased Student’s *t* test analysis was used to compare two groups using GraphPad Prism v10. Experiments were independently repeated ≥3 times (biological repeats), and each experiment had ≥5 technical repeats. The results were considered not significant (ns) when *P* > 0.05 or significant when *P < 0.05, **P < 0.01, ***P < 0.001, and ****P < 0.0001. All graphs were plotted to show the SD of replicates.
