# Supplementary material for: Immune cell infiltration into brain tumor microenvironment is mediated by Rab27-regulated vascular wall integrity
Source: Sci Adv. 2025 May 23;11(21):eadr6940. doi: 10.1126/sciadv.adr6940 (PMC12101492; doi:10.1126/sciadv.adr6940)
Supplement: Supplementary file 1 — Table S1 Figs. S1 to S39 [file sciadv.adr6940_sm.pdf]

Science Advances
AAAS

# Supplementary Materials for

## Immune cell infiltration into brain tumor microenvironment is mediated by Rab27-regulated vascular wall integrity

Lata Adnani *et al.*

Corresponding author: Janusz Rak, janusz.rak@mcgill.ca

*Sci. Adv.* **11**, eadr6940 (2025)
DOI: 10.1126/sciadv.adr6940

**This PDF file includes:**

Table S1
Figs. S1 to S39

| **Acronyms** | **Expansion** |
|---|---|
| BBB | Blood brain barrier |
| BECs | Primary brain endothelial cells isolated from c57bl/6 mice |
| BTB | Blood tumor barrier |
| CAR-NK cells | Chimeric antigen receptor NK-cell therapy |
| CAR-T | Chimeric antigen receptor T-cell therapy |
| CFSE | Carboxyfluorescein succinimidyl ester |
| cyTOF | Cytometry by time of flight |
| dHET | $Rab27a^{+/-}$;$Rab27b^{+/-}$ |
| dKO | $Rab27a^{-/-}$;$Rab27b^{-/-}$ |
| EC | Endothelial cells |
| F-actin | Filamentous actin |
| FACS | Fluorescence-activated cell sorter |
| GL261 | Mouse glioma GL261 |
| GS | Griscelli syndrome |
| GTPase | Guanosine triphosphate |
| ICI | Immune checkpoint inhibitors |
| M01 | 2-[5-(p-Methoxyphenyl)-3-phenyl-4,5-dihydro-1H-pyrazol-1-yl]-2-oxoethyl 1-ethyl-7-methyl-4-oxo-1,8-diaza-3-naphthoate] ; C30H28N4O5 |
| M01A | Nalidixic acid |
| M01B | 3-phenyl-5-methoxyphenyl-1H-pyrazole |
| NCR1 | Natural cytotoxicity triggering receptor 1 |
| Nex-20 | Nexinhib-20 |
| NK-cells | Natural killer cells |
| RBC | Red blood cells |
| scRNA seq | Single cell RNA sequencing |
| T-cells | T-lymphocytes (T, thymus) |
| TECs | Primary brain endothelial cells isolated from tumor bearing c57bl/6 mcie |
| TJs | Tight junctions |
| VEGF | Vascular endothelial growth factor |
| WBC | White blood cells |
| WNT | Wingless-type MMTV integration site family |
| WT | Wild type (C57bl/6); $Rab27a^{+/+}$;$Rab27b^{+/+}$ |
| ZO | Zonula occludens |

**Supplemental Table 1: The list of abbreviations used in the manuscript**

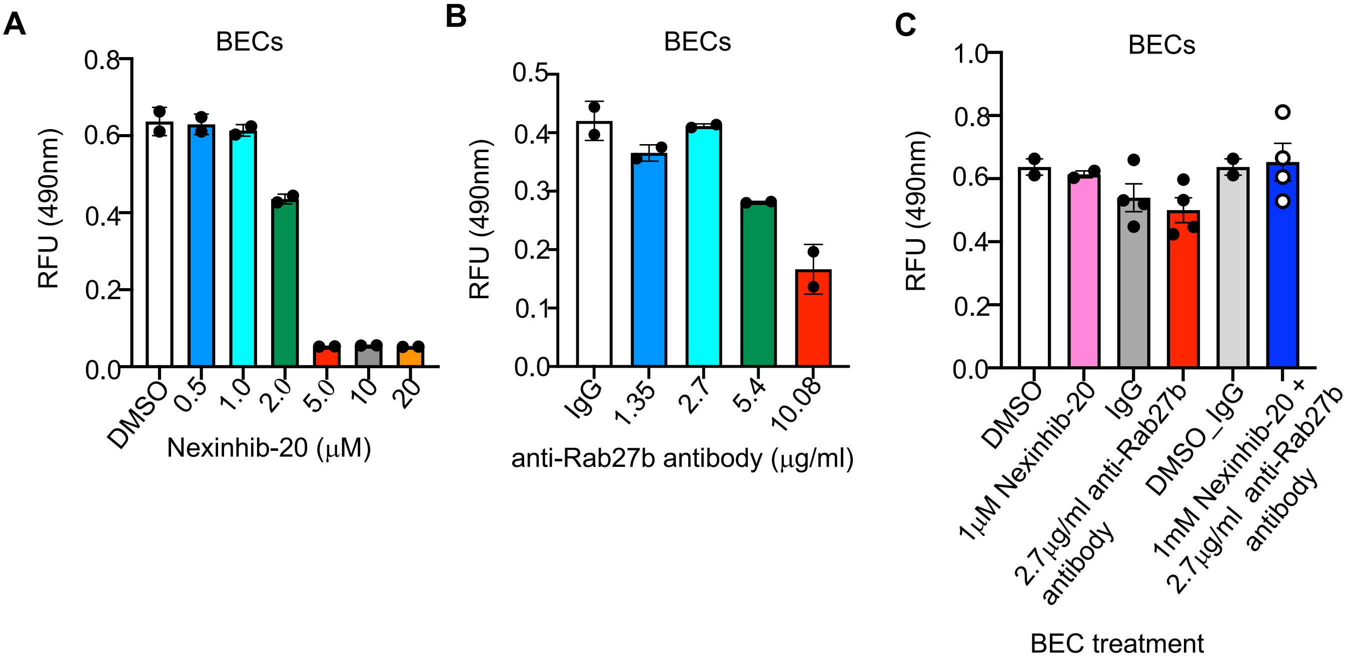


***Fig. S1***

**Supplemental Figure 1.** ***Pharmacological inhibition of Rab27 in brain endothelial cells (BECs).*** **(A)** BECs treated with varying doses of Nexinhib-20 to determine the highest effective devoid of toxicity (MTS assay). **(B)** Dose response of BECs to anti-Rab27b antibody to determine the highest effective concentration devoid of toxicity (MTS assay). **(C)** Responses of BECs to a combination of Nexinhib-20 (1μM) and anti-Rab27b antibody (2.7μg/ml) to validate the absence of toxicity (MTS assay). This combination treatment is used to mimic the impact of genetic Rab27a/b ablation (Rab27 dKO). BECs, are primary brain endothelial cells isolated from brains of C57bl/6 mice; dKO - Rab27a/b double knock-out; RFU - Relative fluorescence units.

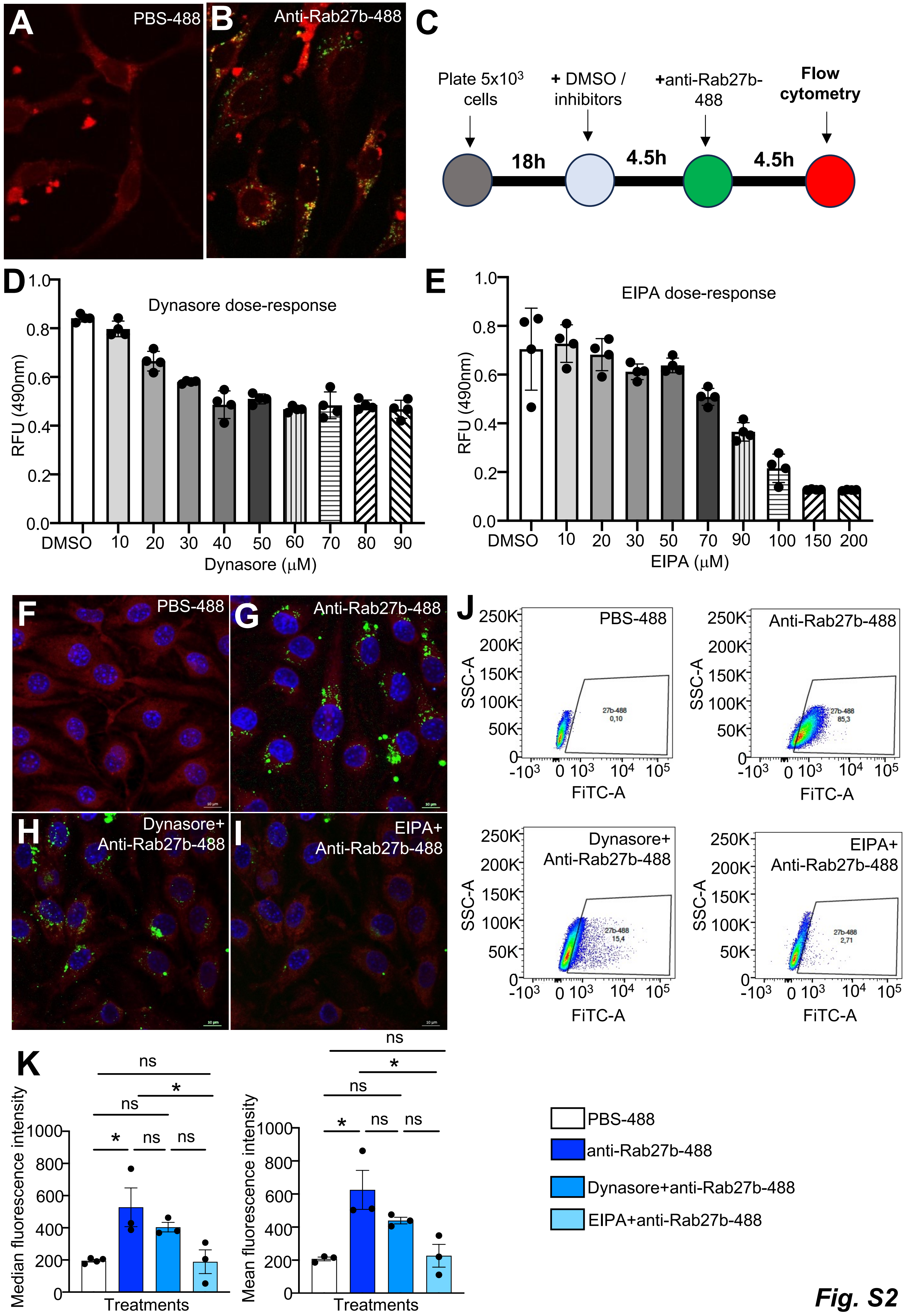

A
PBS-488
B
Anti-Rab27b-488
C
Plate 5x10^3 cells
+ DMSO / inhibitors
+anti-Rab27b-488
Flow cytometry
18h
4.5h
4.5h
D
Dynasore dose-response
RFU (490nm)
Dynasore (μM)
DMSO
E
EIPA dose-response
RFU (490nm)
EIPA (μM)
DMSO
F
PBS-488
G
Anti-Rab27b-488
H
Dynasore+ Anti-Rab27b-488
I
EIPA+ Anti-Rab27b-488
J
PBS-488
27b-488 0,10
Anti-Rab27b-488
27b-488 85,3
Dynasore+ Anti-Rab27b-488
27b-488 15,4
EIPA+ Anti-Rab27b-488
27b-488 2,71
SSC-A
FiTC-A
K
Median fluorescence intensity
Mean fluorescence intensity
Treatments
ns
*
PBS-488
anti-Rab27b-488
Dynasore+anti-Rab27b-488
EIPA+anti-Rab27b-488


*Fig. S2*

**Supplemental Figure 2:** ***Uptake of anti-Rab27b antibody by primary brain endothelial cells.*** **(A,B)** Confocal microscopy performed 6.5 hours after incubating BECs with PBS **(A)** and anti-Rab27b-488 (green**, B)** documents the uptake of the antibody. **(C)** Diagram of experimental design to explore anti-Rab27b-488 uptake after pre-treatment of the cells with either endocytosis or micropinocytosis inhibitors. **(D,E)** MTS assay to establish dose-response curve for endocytosis inhibitor, dynasore **(D)** and macropinocytosis inhibitor, EIPA **(E)**. **(F-I)** Confocal images of BECs treated with indicated agents: PBS **(F)**, anti-Rab27b-488 antibody **(G)**, dynasore pre-treatment prior to addition of anti-Rab27b-488 antibody **(H)**, EIPA pre-treatment prior to addition of anti-Rab27b-488 antibody **(I)**. **(J)** Flow cytometry of BECs treated with PBS, anti-Rab27b-488 antibody, dynasore pre-treatment followed by anti-Rab27b-488 antibody, and EIPA pre-treatment followed by anti-Rab27b-488 antibody. **(K)** Quantification of median (left) and mean (right) fluorescent intensity of BECs exposed to anti-Rab27b-488 antibody under indicated conditions. RFU – Relative fluorescence units. $P < 0.05$ (*); $P < 0.01$ (**); $P < 0.001$ (***) and $P < 0.0001$ (****).

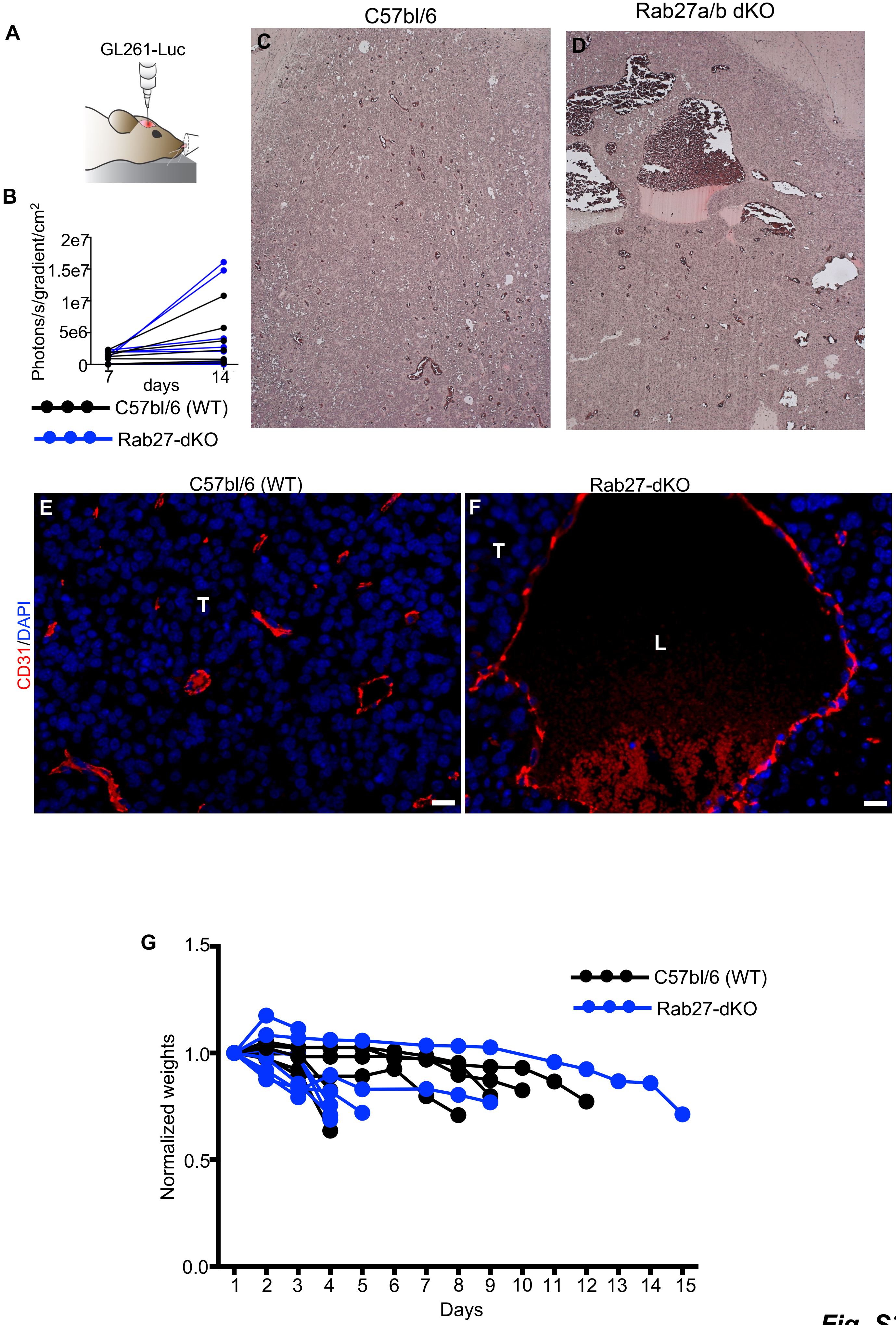
A
GL261-Luc
B
Photons/s/gradient/cm2
2e7
1.5e7
1e7
5e6
0
7
days
14
C57bl/6 (WT)
Rab27-dKO
C57bl/6
C
Rab27a/b dKO
D
C57bl/6 (WT)
E
T
Rab27-dKO
F
T
L
CD31/DAPI
G
1.5
1.0
0.5
0.0
Normalized weights
C57bl/6 (WT)
Rab27-dKO
1
2
3
4
5
6
7
8
9
10
11
12
13
14
15
Days

***Fig. S3***

**Supplemental Figure 3:** ***Aberrant vasculature in mouse glioblastoma implanted into Rab27-deficient animals.*** **(A)** Cartoon of intracranial inoculation of GL261-Luc glioblastoma cells. **(B)** Quantification of time dependent changes in bioluminiscence of WT (black) or dKO (blue) following GL261-Luc inoculation after injection of luciferin substrate. **(C,D)** Hematoxylin/Eosin (H&E) staining of brain tumor tissues from C57bl/6-WT **(C)** or Rab27-dKO **(D)** recipients of GL261-Luc brain tumor cells. **(E,F)** CD31 staining (red) of brain tumor tissues in C57bl/6-WT **(E)** or Rab27-dKO **(F)** mice harbouring GL261-Luc brain tumors (DAPI in blue). **(G)** Weights of WT (black) and dKO (blue) mice post intracranial injections of GL261-Luc cells; WT - wild-type; dKO - Rab27a/b double knock out; L – Lumen; T - Tumor. Not significant (ns); $P < 0.05$ (*); $P < 0.01$ (**); $P < 0.001$ (***) and $P < 0.0001$ (****).

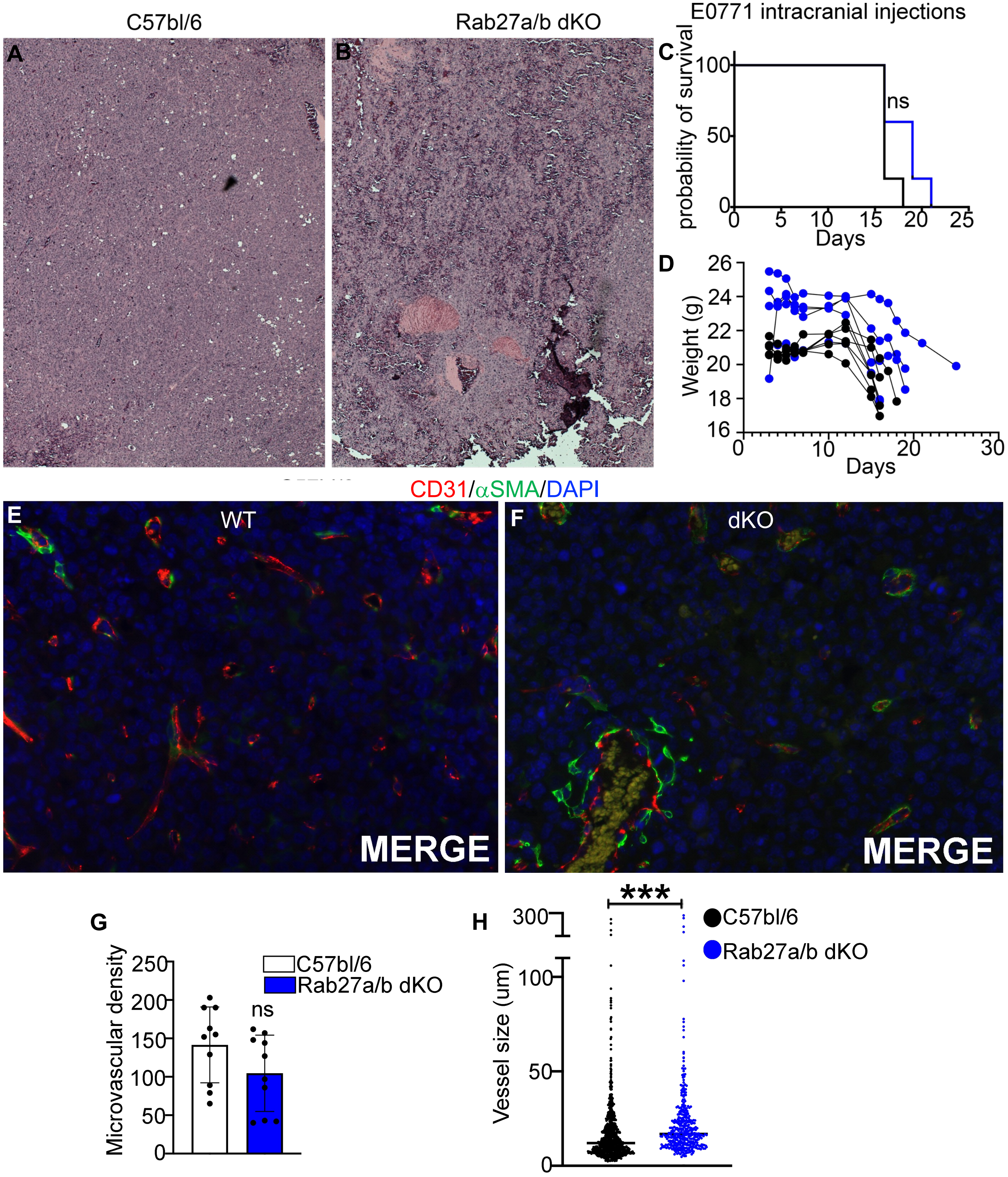
C57bl/6
Rab27a/b dKO
E0771 intracranial injections
A
B
C
probability of survival
ns
Days
D
Weight (g)
Days
CD31/αSMA/DAPI
E
WT
MERGE
F
dKO
MERGE
G
Microvascular density
C57bl/6
Rab27a/b dKO
ns
H
Vessel size (um)
***
C57bl/6
Rab27a/b dKO

*Fig. S4*

**Supplemental Figure 4.** ***Rab27-deficient mice mount aberrant vascular responses in a model of breast cancer metastasis to the brain*. (A,B)** H&E staining of brain tumor tissues in C57bl/6-WT **(A)** or Rab27-dKO **(B)** mice harbouring intracranial transplants of mouse breast cancer cells, E0771. **(C)** Kaplan-Meyer survival plot for mice harbouring intracranial E0771 tumors (WT mice – black line; dKO mice – blue line); **(D)** Time-dependent measurement of mouse weight of WT (black) and dKO (blue) mice post intracranial injections of E0771 cells. **(E-F)** Staining of vascular structures in E0771 brain tumors: CD31 (red), αSMA (green) and DAPI (blue); representative images are shown of tumors in C57bl/6-WT **(E)** and Rab27-dKO **(F)** mice. **(G)** Quantification of microvascular density of brain tumor tissues in WT (white) and dKO (blue) mice harbouring intracranial E0771 lesions. **(H)** Quantification of vessel size for brain tumor tissues of WT (white) and dKO (blue) mice with E0771 tumors. C57bl/6 WT - wild-type; dKO - Rab27 double knock out; each group, n=/>5; Not significant (ns); $P < 0.05$ (*); $P < 0.01$ (**); $P < 0.001$ (***) and $P < 0.0001$ (****).

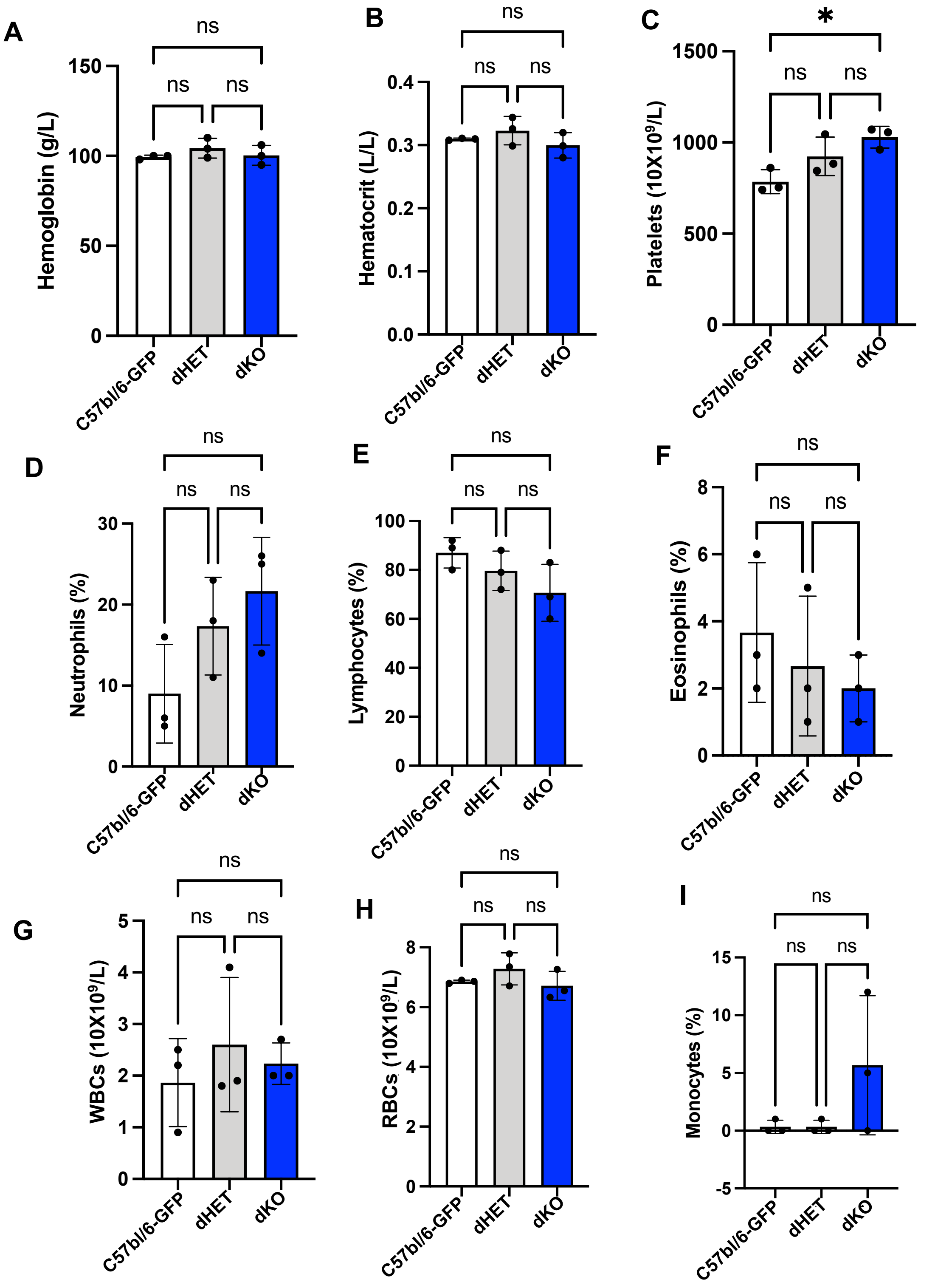


*Fig. S5*

**Supplemental Figure 5.** ***Unchanged blood counts in WT, dHET and dKO mice.*** **(a-g)** Systemic blood draws from WT (white), dHET (grey) and dKO (blue) revealed little to no change in the levels of hemoglobin **(A)**, hematocrit **(B)**, platelets **(C)**, neutrophils **(D)**, lymphocytes **(E)**, eosinophils **(F)**, white blood cells (WBCs) **(G)**, red blood cells (RBCs) **(H)** and monocytes **(I)**. WT - wild type (C57bl/6); dHET - Rab27 double heterozygote; dKO - Rab27 double knock out; Not significant (ns); $P < 0.05$ (*); $P < 0.01$ (**); $P < 0.001$ (***) and $P < 0.0001$ (****).

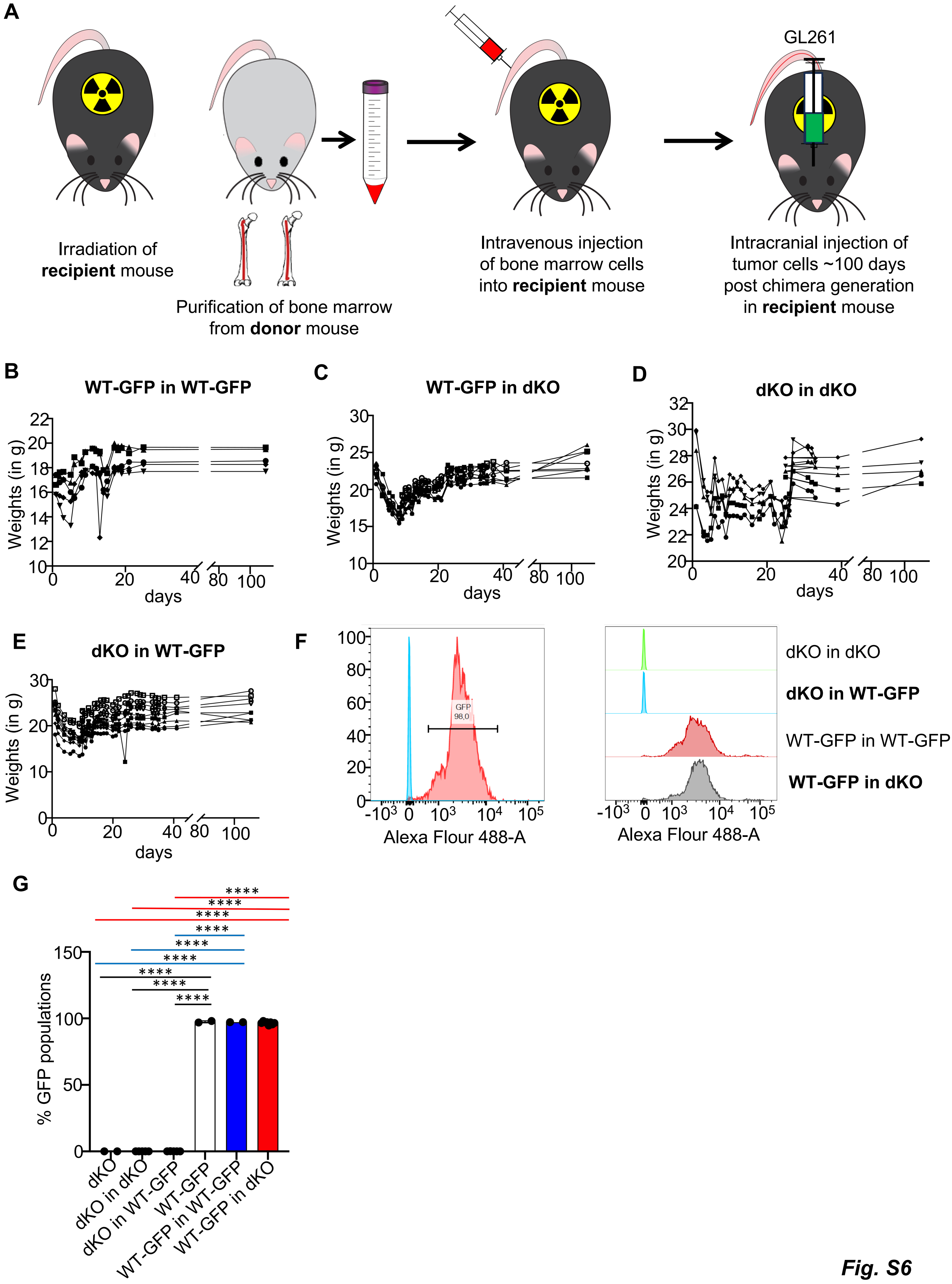
A
GL261
Irradiation of **recipient** mouse
Purification of bone marrow from **donor** mouse
Intravenous injection of bone marrow cells into **recipient** mouse
Intracranial injection of tumor cells ~100 days post chimera generation in **recipient** mouse
B
WT-GFP in WT-GFP
Weights (in g)
days
C
WT-GFP in dKO
D
dKO in dKO
E
dKO in WT-GFP
F
GFP 98,0
Alexa Flour 488-A
dKO in dKO
dKO in WT-GFP
WT-GFP in WT-GFP
WT-GFP in dKO
G
% GFP populations
dKO
dKO in dKO
dKO in WT-GFP
WT-GFP
WT-GFP in WT-GFP
WT-GFP in dKO

*Fig. S6*

**Supplemental Figure 6.** ***Generation and characterization of murine bone-marrow chimeras*****. (A)** Schematic illustrating bone marrow chimeric mouse models for the analysis of intracranial GL261-Luc tumors. **(B-E)** Weight assessment after irradiation of: WT-GFP mice and repopulation with WT-GFP bone marrow **(B)**, dKO mice and repopulation with WT-GFP bone marrow **(C)**, dKO mice and repopulation with dKO bone marrow **(D)**, WT-GFP mice and repopulation with dKO bone marrow **(E)**. **(F)** FACS analysis of peripheral blood to confirm the presence of GFP cells in non-GFP dKO chimeric mice and absence of GFP in the otherwise GFP expressing WT-GFP mice to validate successful generation of chimeric mouse models systemically. **(G)** Quantification of %$GFP^+$ cells in the blood of chimeric mouse models. WT-GFP - wild type (C57bl/6)-GFP; dKO - Rab27 double knock out. For each group, n=5 mice were generated, Not significant (ns); $P < 0.05$ (*); $P < 0.01$ (**); $P < 0.001$ (***) and $P < 0.0001$ (****).

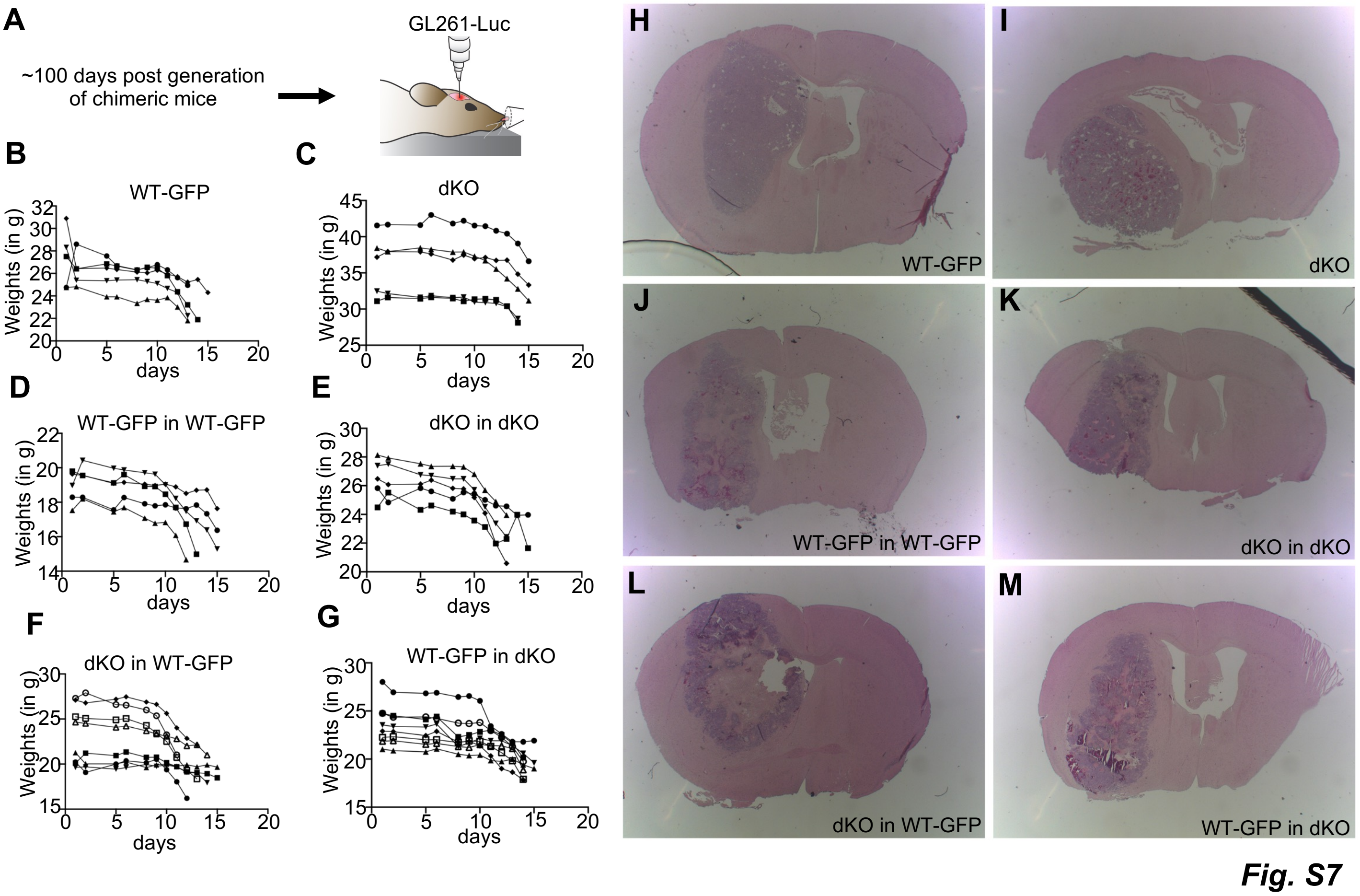


**Supplemental Figure 7.** ***Intracranial glioma progression in bone-marrow chimeras*****.** **(A)** Schematic depicting GL261-Luc brain tumor model in chimeric mice. **(B-G)** Weight assessment of : WT-GFP **(B)**, dKO **(C)**, and mice after irradiation and bone marrow repopulation: (**D**) WT-GFP mice repopulated with WT-GFP bone marrow; (**E**) dKO mice and repopulated with dKO bone marrow; (**F**) WT-GFP mice repopulated with dKO bone marrow; (**G**) dKO mice repopulated with WT-GFP bone marrow. **(H-M)** tumor containing mouse brains stained for H&E to visualize the morphology of tumors: WT-GFP **(H)**, dKO **(I)**, WT-GFP mice repopulated with WT-GFP bone marrow **(J)**, dKO mice repopulated with dKO bone marrow **(K)**, WT-GFP mice repopulated with dKO bone marrow **(L)**, and dKO repopulated with WT-GFP bone marrow **(M)**. WT-GFP - wild type (C57bl/6)-GFP; dKO - Rab27 double knock-out. For each group, n=5 mice were injected intracranially.

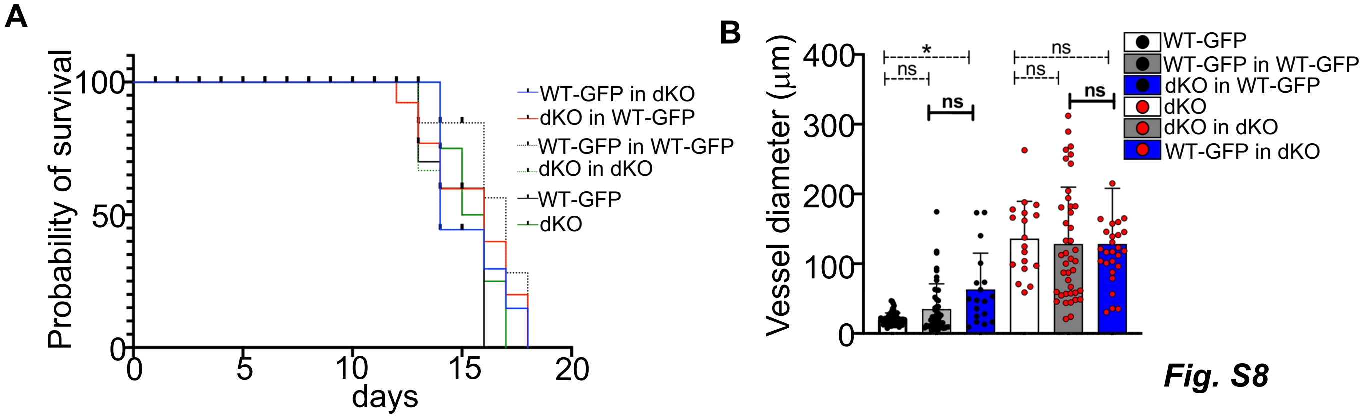


**Supplemental Figure 8.** ***Rab27 deficiency-induced dysmorphic vasculature in glioblastoma or survival of tumor bearing mice are not affected by systemic replacement of wild type bone marrow.*** **(A)** Kaplan-Meyer survival plot of chimeric mice shows no survival benefit upon systemic replacement of bone marrow precursors of myeloid cells and platelets in dKO mice. **(B)** Vessel diameters of chimeric mice at clinical end points after intracranial injections of GL261-Luc glioma cells. WT-GFP - wild type (C57bl/6)-GFP; dKO - Rab27 double knock out. For each group, n=5 mice were quantified; Not significant (ns); $P < 0.05$ (*); $P < 0.01$ (**); $P < 0.001$ (***) and $P < 0.0001$ (****).

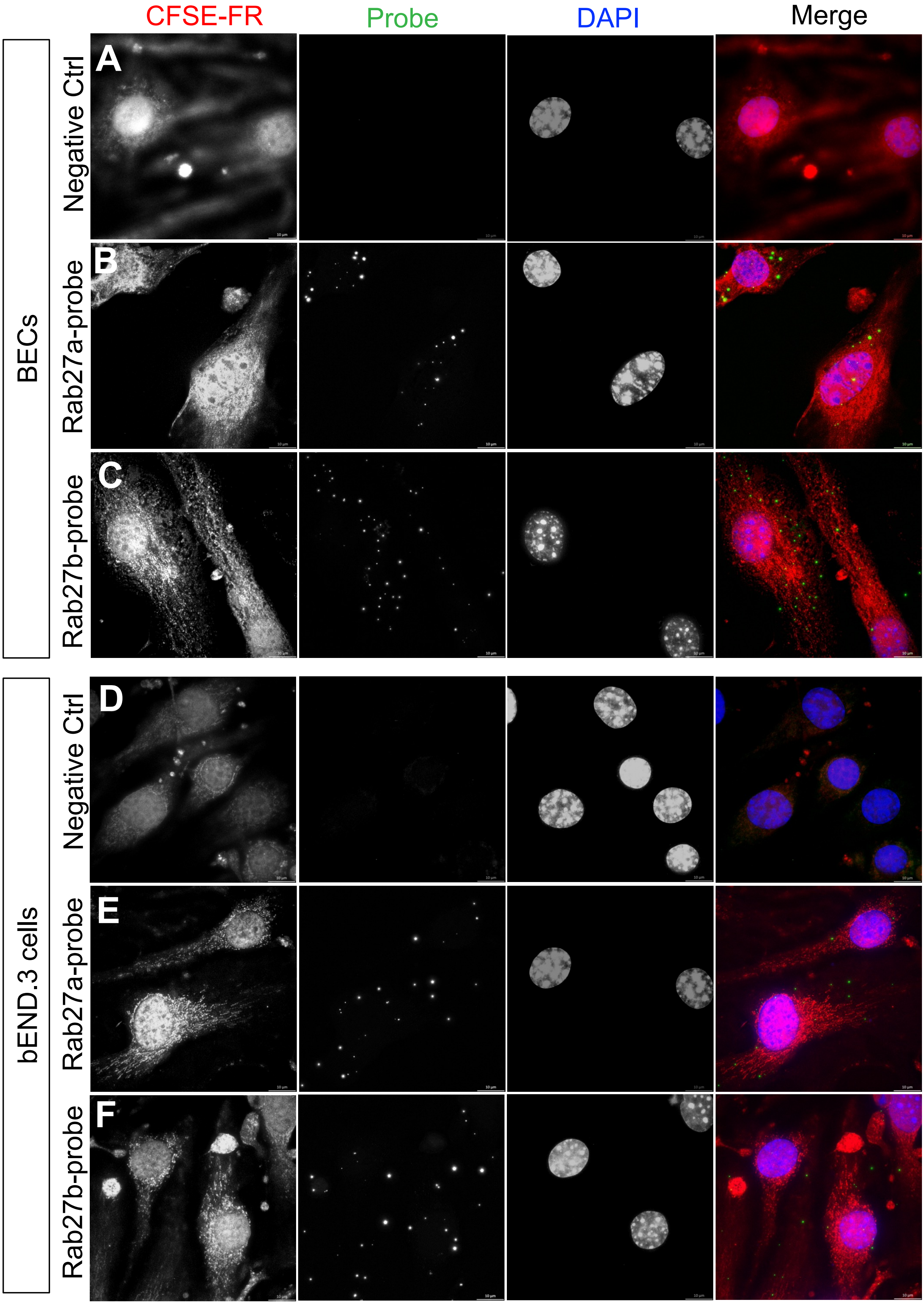
CFSE-FR
Probe
DAPI
Merge
BECs
bEND.3 cells
Negative Ctrl
Rab27a-probe
Rab27b-probe
A
B
C
D
E
F

*Fig. S9*

**Supplemental Figure 9.** ***RNA expression of Rab27a and Rab27b in mouse brain endothelial cells.*** **(A-C)** RNA *In situ* hybridization for BECs: without a probe **(A)**, with Rab27a-probe **(B)**, and with Rab27b-probe **(C)**. **(D-F)** RNA *In situ* hybridization for bEND.3 cells: without a probe **(D)**, with Rab27a-probe **(E)**, and with Rab27b-probe **(F)**.

| | DDCt Rab27a | | DDCt Rab27b |
|---|---|---|---|
| | | | |
| dHET BEC | 0.052313726 | | 0.243163737 |
| | 0.602207814 | | 0.664342907 |
| | 0.262126176 | | 0.137261602 |
| dKO BEC | 0.006038196 | | 0.106949378 |
| | 0.000372192 | | 0.006150844 |
| | 0.000726542 | | 0.283220971 |
| | 0.002619184 | | |
| dHET TEC | 0.232182853 | | 0.032017401 |
| | 0.115289524 | | 0.254369923 |
| | 0.249711355 | | |
| dKO TEC | 0.001309592 | | 0.032464347 |
| | 0.0000562937 | | 0.003150944 |

***Fig. S10***

**Supplemental Figure 10.** ***Quantification of RNA expression of Rab27a and Rab27b in endothelial cells.*** BECs and TECs were isolated using FACS and lysed to perform qPCR to quantify mRNA expression levels of Rab27a and Rab27b. Delta/delta Ct (DDCt) was used to express the amounts of detected mRNA (n = 2-3, as indicated).

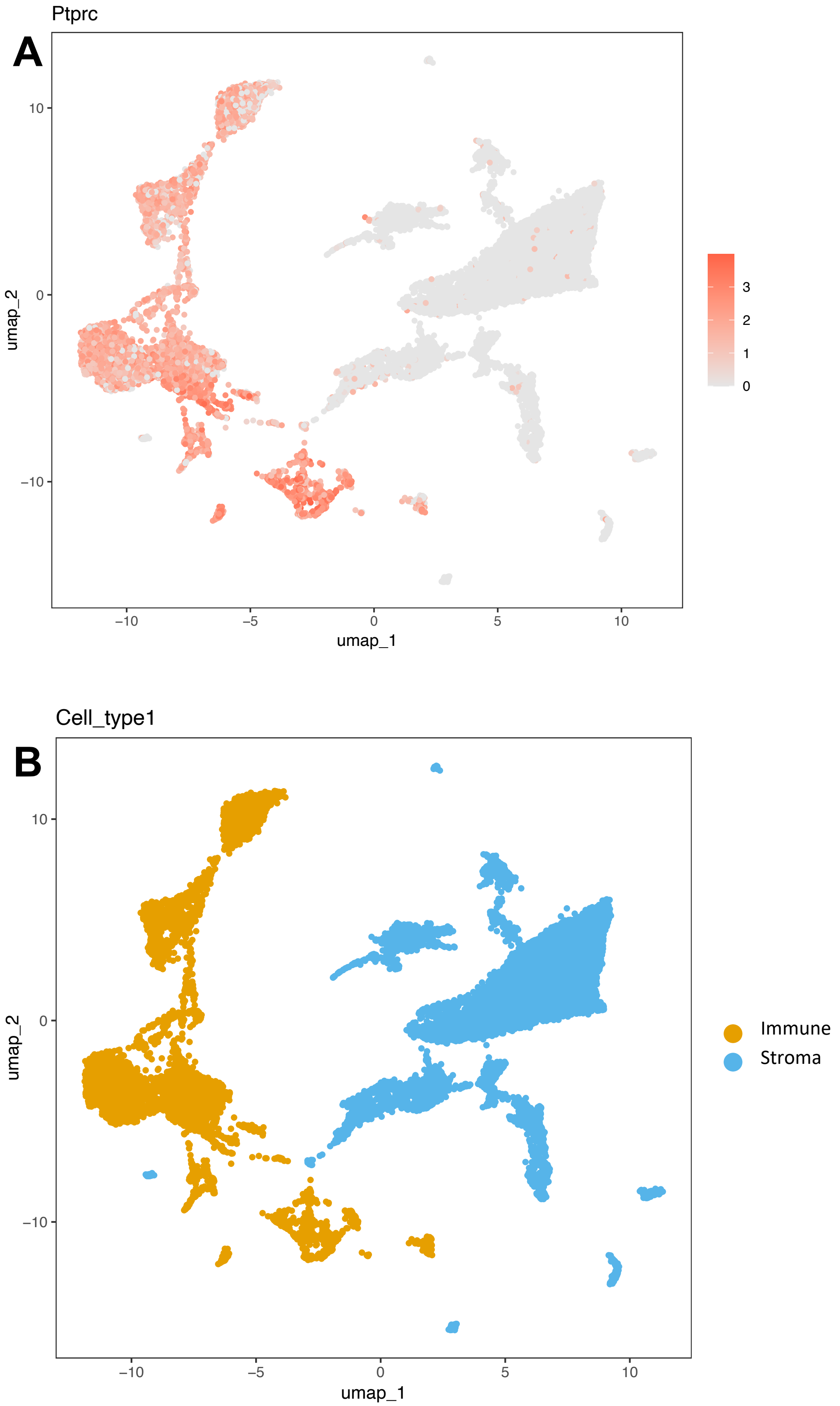


***Fig. S11***

**Supplemental Figure 11.** ***Single cell sequencing of primary brain endothelial cells from Rab27 deficient mice reveals distinct transcriptional signatures.*** **(A)** Dimensionality reduction plot identified clustering of single cells based on the expression of immune cell markers such as Ptprc. **(B)** Dimensionality reduction plot to cluster the distinct cell populations.

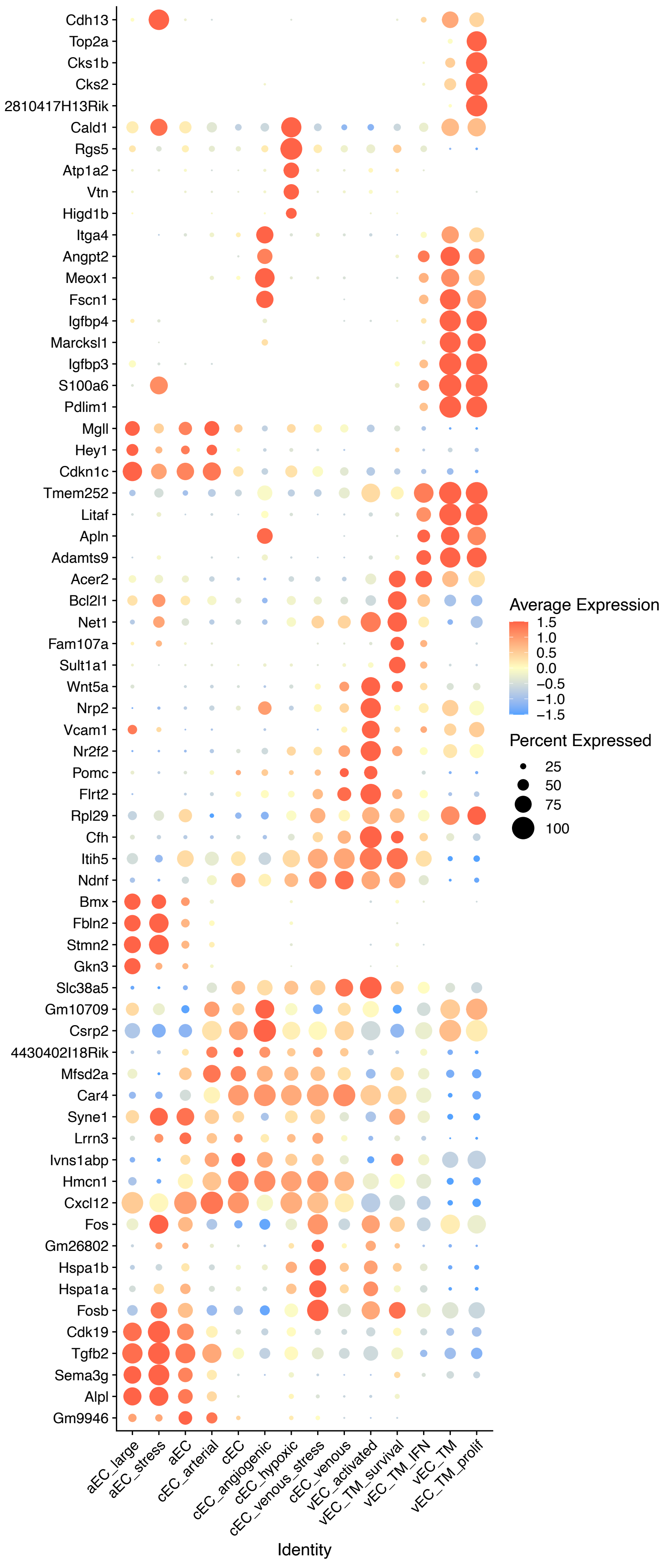
Cdh13
Top2a
Cks1b
Cks2
2810417H13Rik
Cald1
Rgs5
Atp1a2
Vtn
Higd1b
Itga4
Angpt2
Meox1
Fscn1
Igfbp4
Marcksl1
Igfbp3
S100a6
Pdlim1
Mgll
Hey1
Cdkn1c
Tmem252
Litaf
Apln
Adamts9
Acer2
Bcl2l1
Net1
Fam107a
Sult1a1
Wnt5a
Nrp2
Vcam1
Nr2f2
Pomc
Flrt2
Rpl29
Cfh
Itih5
Ndnf
Bmx
Fbln2
Stmn2
Gkn3
Slc38a5
Gm10709
Csrp2
4430402I18Rik
Mfsd2a
Car4
Syne1
Lrrn3
Ivns1abp
Hmcn1
Cxcl12
Fos
Gm26802
Hspa1b
Hspa1a
Fosb
Cdk19
Tgfb2
Sema3g
Alpl
Gm9946
aEC_large
aEC_stress
aEC
cEC_arterial
cEC
cEC_angiogenic
cEC_hypoxic
cEC_venous_stress
cEC_venous
vEC_activated
vEC_TM_survival
vEC_TM_IFN
vEC_TM
vEC_TM_prolif
Identity
Average Expression
1.5
1.0
0.5
0.0
−0.5
−1.0
−1.5
Percent Expressed
25
50
75
100

**Fig. S12**

**Supplemental Figure 12.** ***Transcriptional signatures of primary brain endothelial cells as revealed by single cell RNA sequencing.*** Dot plot to compare the different genes expressed (y-axis) in the different endothelial cell-subtype clusters The size of the dot is based on the percent of the gene expression as is shown in the key. Terms used to define endothelial cell clusters (see text for sources): aEC - arterial endothelial cells; cEC - capillary endothelial cells; cEC_arterial - capillary endothelial cells molecularly resembling cells found in arterial branches; cEC_venous - capillary endothelial cells close to the phenotype of endothelial cells in veins; vEC – venous endothelial cells; aEC_large - large artery endothelial cells; aEC_stress - arterial endothelial cells expresseing the stress-associated phenotype; cEC_angiogenic - angiogenic capillary endothelial cells; cEC_venous_ stress - capillary endothelial cells expressing some features of venous endothelial cells and stress responses; vEC_activated - activated venous endothelial cells; vEC_TM_survival - venous endothelial cells with features specific to the tumor microenvironment and enriched for survival markers; vEC_TM_IFN - venous endothelial cells with a phenotype associated with the tumor microenvironment and enriched in markers of interferon/inflammation pathways; vEC_TM - venous endothelial cells with features associated with the tumor microenvironment; vEC_TM_prolif - venous endothelial cells in the tumor microenvironment enriched in proliferation markers.

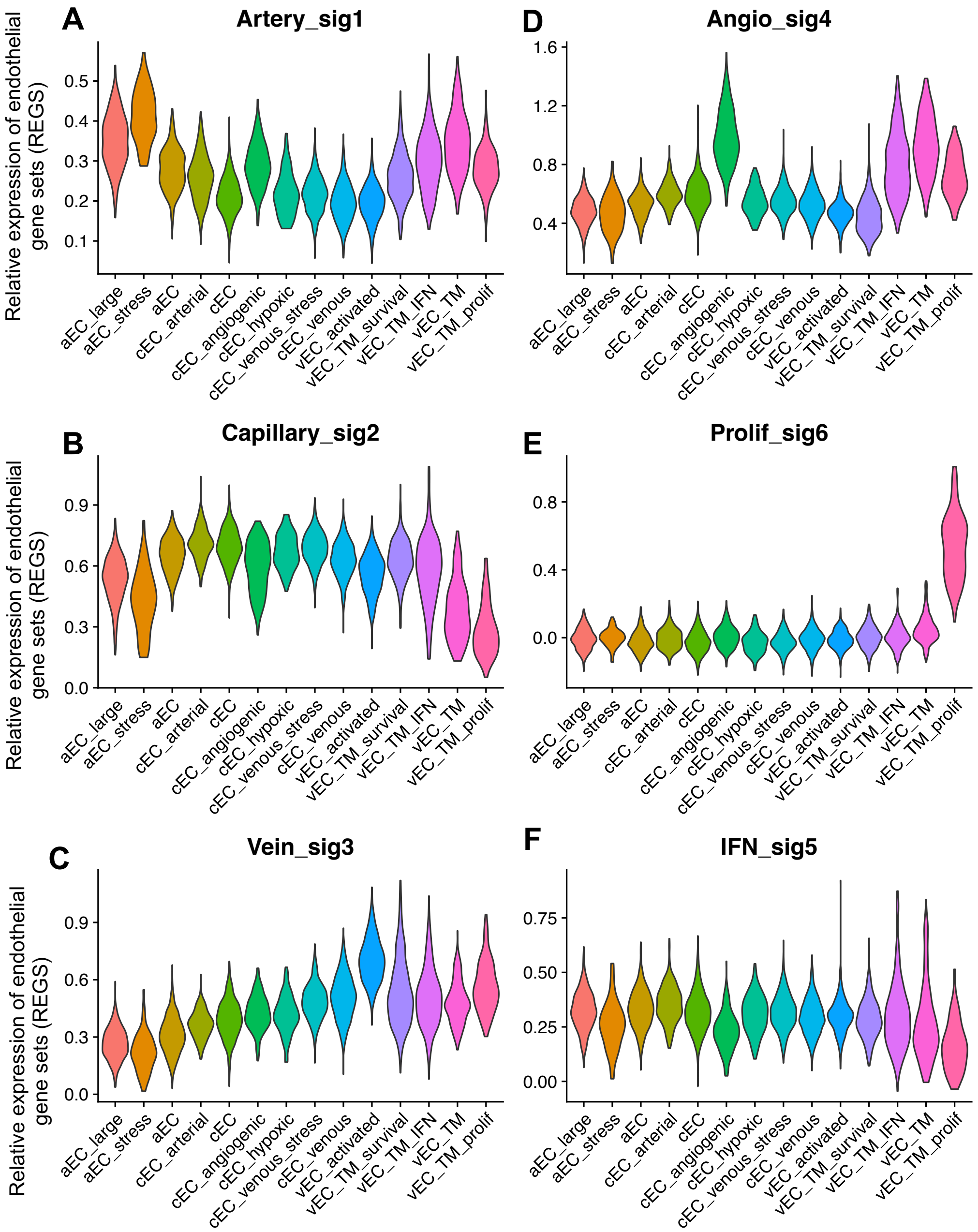


***Fig. S13***

**Supplemental Figure 13.** ***Transcriptional phenotypes of mouse endothelial cell subpopulations.*** **(A-C)** Violin plots across the different endothelial cell type clusters for: artery **(A)**, capillary **(B)** and vein endothelium **(C)**. **(D-F)** Violin plots across the different functional endothelial cell signatures: angiogenic **(D)**, proliferative **(E)** and inflammatory **(F);** terms adopted from *(49)* represent known endothelial gene expression programs**.** Abbreviations: aEC – arterial endothelial cells; cEC - capillary endothelial cells; cEC_arterial - capillary endothelial cells close to arterial branches; cEC_venous - capillary endothelial cells close to the veins; vEC - venous endothelial cells; aEC_large - large artery endothelial cells; aEC_stress - arterial endothelial cells exhibiting stress response; cEC_angiogenic - angiogenic capillary endothelial cells; cEC_venous_ stress - capillary endothelial cells close to venous branches with stress response phenotype; vEC_activated - activated venous endothelial cells; vEC_TM_survival - venous endothelial cells with phenotypes specific for the tumor microenvironment and markers of survival; vEC_TM_IFN - venous endothelial cells in the tumor microenvironment enriched in markers of inflammation; vEC_TM - venous endothelial cells in the tumor microenvironment; vEC_TM_prolif - venous endothelial cells in the tumor microenvironment enriched in proliferation markers. Relative endothelial gene expression scores (REGS) were calculated by AddModuleScore in Seurat package. It is averaged expression of each gene set normalized by randomly selected control gene sets, regardless of the Rab27 status.

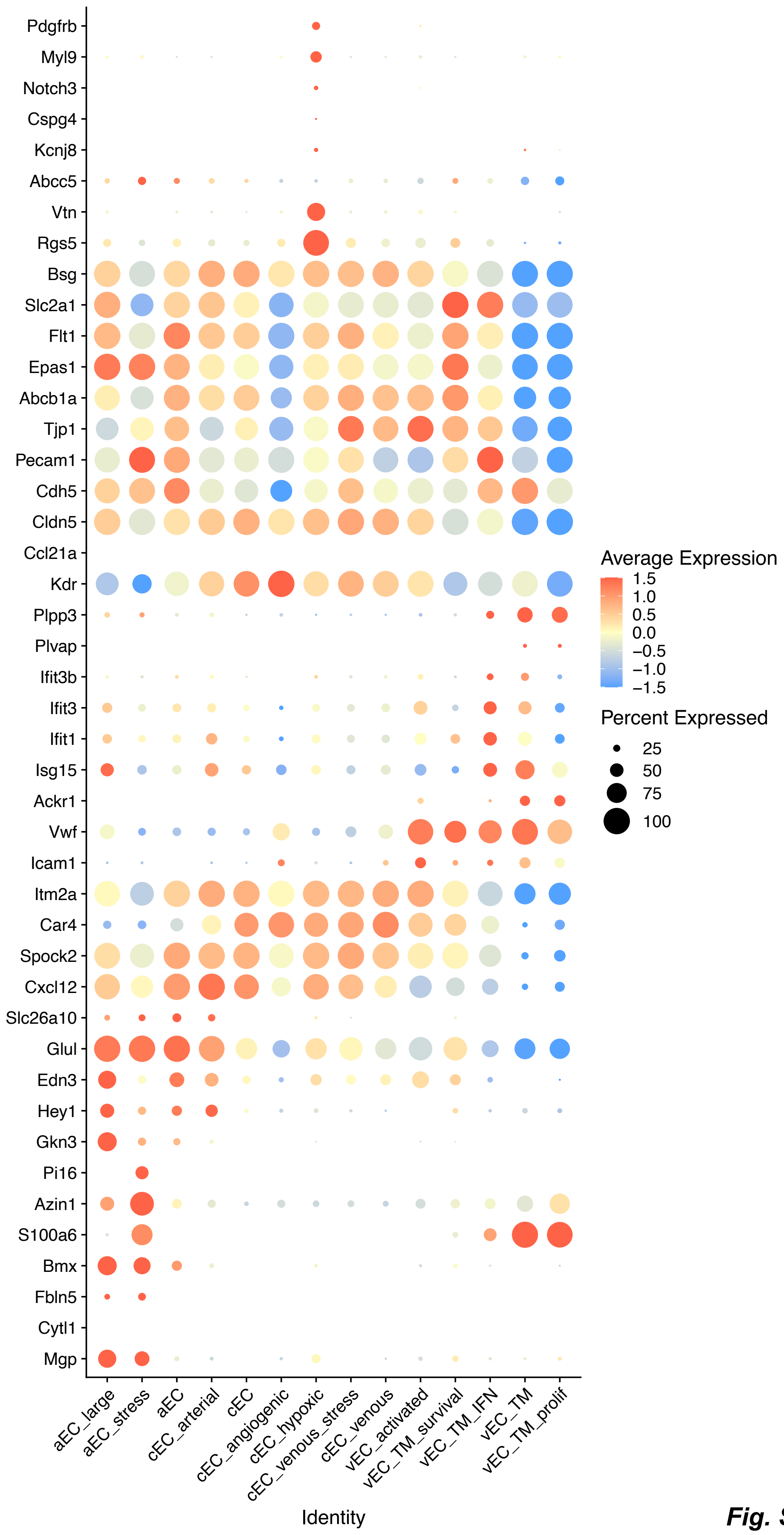
Pdgfrb
Myl9
Notch3
Cspg4
Kcnj8
Abcc5
Vtn
Rgs5
Bsg
Slc2a1
Flt1
Epas1
Abcb1a
Tjp1
Pecam1
Cdh5
Cldn5
Ccl21a
Kdr
Plpp3
Plvap
Ifit3b
Ifit3
Ifit1
Isg15
Ackr1
Vwf
Icam1
Itm2a
Car4
Spock2
Cxcl12
Slc26a10
Glul
Edn3
Hey1
Gkn3
Pi16
Azin1
S100a6
Bmx
Fbln5
Cytl1
Mgp
aEC_large
aEC_stress
aEC
cEC_arterial
cEC
cEC_angiogenic
cEC_hypoxic
cEC_venous_stress
cEC_venous
vEC_activated
vEC_TM_survival
vEC_TM_IFN
vEC_TM
vEC_TM_prolif
Identity
Average Expression
1.5
1.0
0.5
0.0
−0.5
−1.0
−1.5
Percent Expressed
25
50
75
100

***Fig. S14***

**Supplemental Figure 14.** ***Transcriptional signatures of primary brain endothelial cells as revealed by single cell RNA sequencing.*** Dot plot to compare the different individual genes expressed (y-axis) in the different endothelial cell-type clusters. The size of the dot is based on the percent of gene expression as is shown in the key; terms adopted from *(49)* represent known endothelial gene expression programs. Abbreviations: aEC - artery endothelial cells; cEC - capillary endothelial cells; cEC_arterial - capillary endothelial cells close to arterial branches; cEC_venous - capillary endothelial cells close to venous branches; vEC - venous endothelial cells; aEC_large - large artery endothelial cells; aEC_stress - arterial endothelial cells with features of stress response; cEC_angiogenic - angiogenic capillary endothelial cells; cEC_venous_stress - capillary endothelial cells close to the branches of the veins exhibiting features of stress response; vEC_activated - activated venous endothelial cells; vEC_TM_survival - venous endothelial cells in the tumor microenvironment with markers of cell survival; vEC_TM_IFN - venous endothelial cells in the tumor microenvironment enriched in markers of inflammation; vEC_TM - venous endothelial cells with features specific for tumor microenvironment; vEC_TM_prolif - venous endothelial cells with features specific for tumor microenvironment and enriched in proliferation markers.

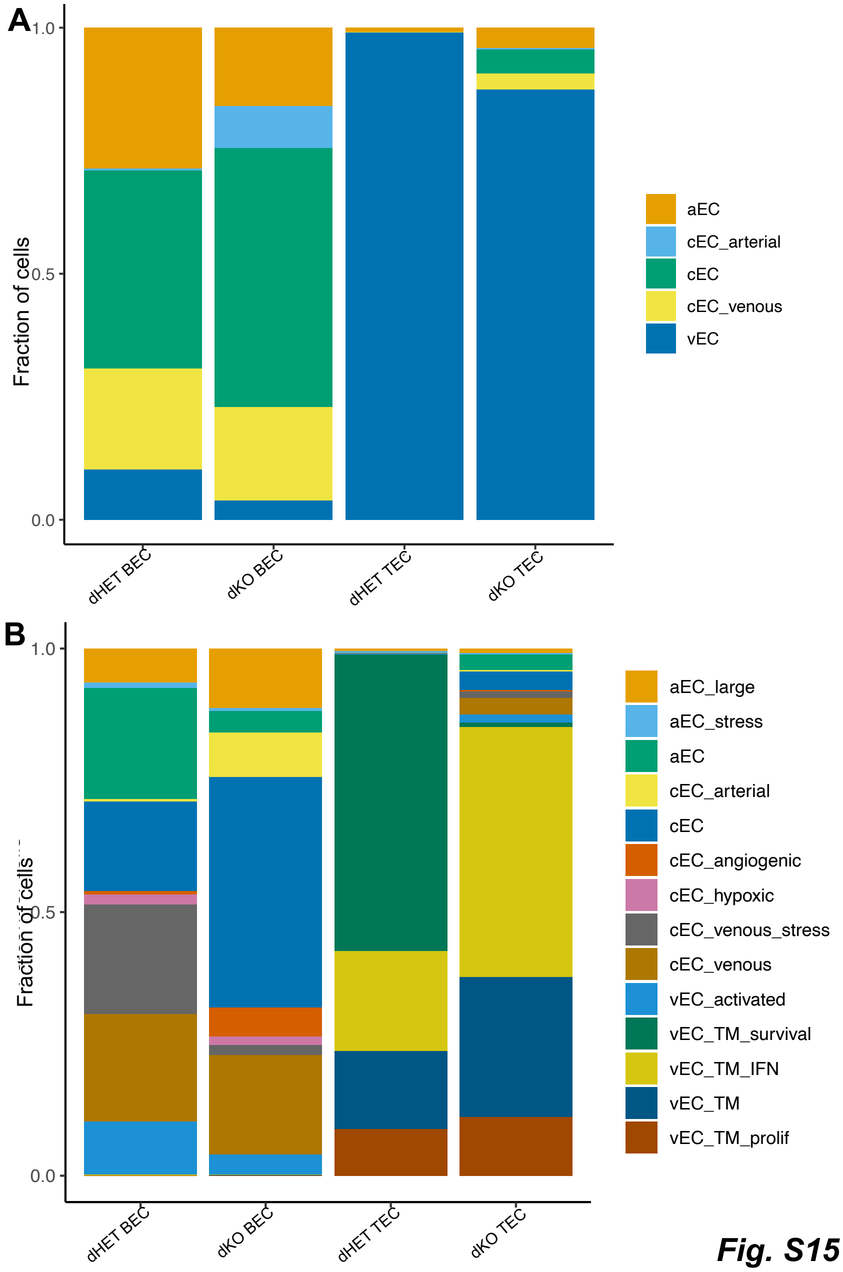


***Fig. S15***

**Supplemental Figure 15.** ***Single cell RNA sequencing reveals distinct transcriptional clusters for normal and tumor-associated endothelial cells in wild type and Rab27-deficient mice.*** **(A)** Fractions of normal brain (BEC) and brain tumor-associated (TEC) endothelial cell clusters in Rab27-proficient (dHET) and deficient (dKO) mice. **(B)** Cell clusters assigned to known endothelial phenotypes (49) across BECs *versus* TECs in dHET *versus* dKO mice. Abbreviations: BECs - primary brain endothelial cells; TECs - primary tumor-derived brain endothelial cells; dHET - Rab27 double heterozygote mice; dKO - Rab27a/b double knock out; aEC - arterial endothelial cells; cEC - capillary endothelial cells; cEC_arterial - capillary endothelial cells close to the arterial branches;; cEC_venous - capillary endothelial cells close to veinous branches; vEC - venous endothelial cells; aEC_large - large artery endothelial cells; aEC_stress - arterial endothelial cells with features of stress response; cEC_angiogenic - angiogenic capillary endothelial cells; cEC_venous_stress - capillary endothelial cells close to the branches of veins with features of stress response; vEC_activated - activated venous endothelial cells; vEC_TM_survival - venous endothelial cells with features specific to the tumor microenvironment and enriched in markers of cell survival; vEC_TM_IFN - venous endothelial cells with features specific for the tumor microenvironment and enriched in markers of inflammation; vEC_TM - venous endothelial cells with features specific for the tumor microenvironment; vEC_TM_prolif - venous endothelial cells with features specific to the tumor microenvironment and enriched in proliferation markers.

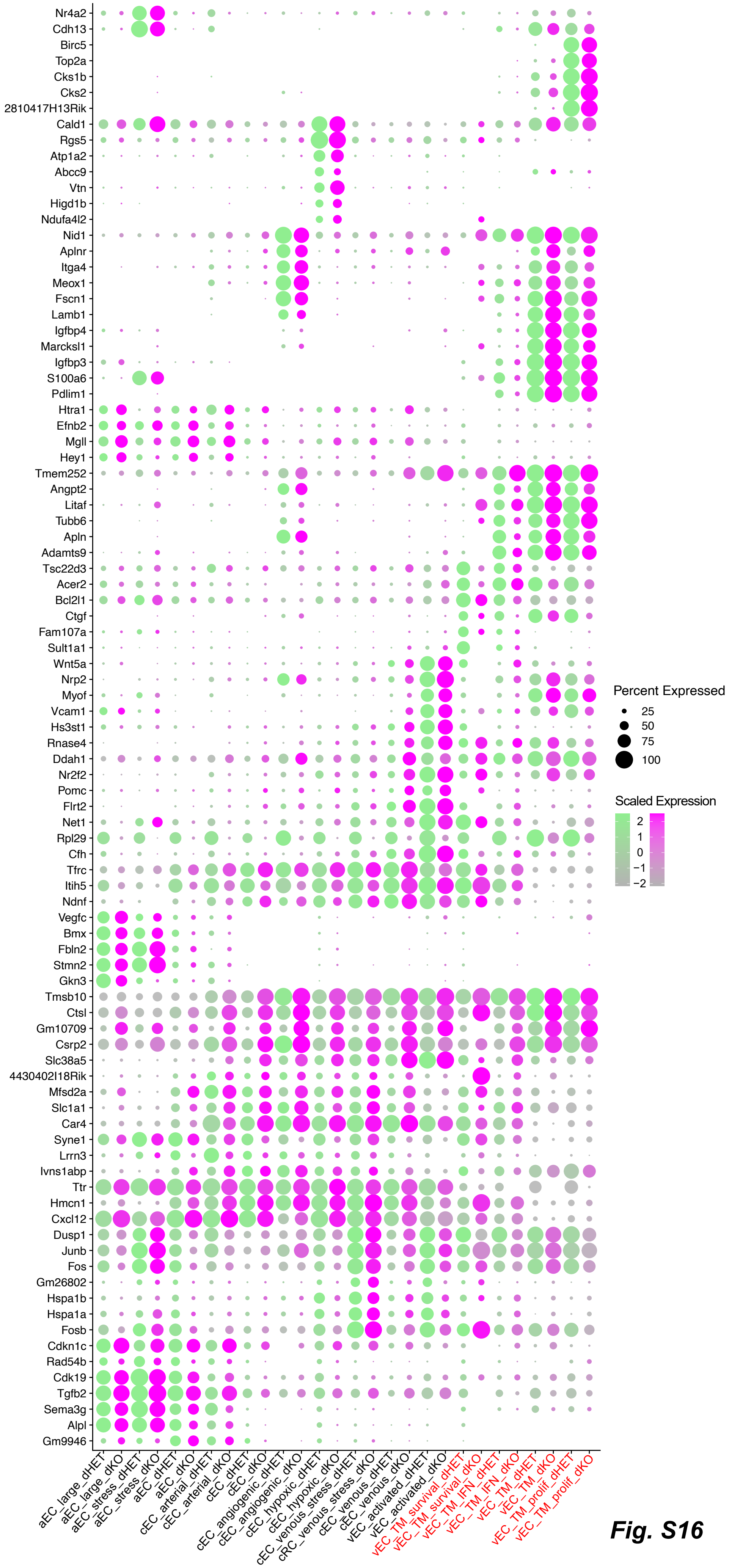

Nr4a2
Cdh13
Birc5
Top2a
Cks1b
Cks2
2810417H13Rik
Cald1
Rgs5
Atp1a2
Abcc9
Vtn
Higd1b
Ndufa4l2
Nid1
Aplnr
Itga4
Meox1
Fscn1
Lamb1
Igfbp4
Marcksl1
Igfbp3
S100a6
Pdlim1
Htra1
Efnb2
Mgll
Hey1
Tmem252
Angpt2
Litaf
Tubb6
Apln
Adamts9
Tsc22d3
Acer2
Bcl2l1
Ctgf
Fam107a
Sult1a1
Wnt5a
Nrp2
Myof
Vcam1
Hs3st1
Rnase4
Ddah1
Nr2f2
Pomc
Flrt2
Net1
Rpl29
Cfh
Tfrc
Itih5
Ndnf
Vegfc
Bmx
Fbln2
Stmn2
Gkn3
Tmsb10
Ctsl
Gm10709
Csrp2
Slc38a5
4430402I18Rik
Mfsd2a
Slc1a1
Car4
Syne1
Lrrn3
Ivns1abp
Ttr
Hmcn1
Cxcl12
Dusp1
Junb
Fos
Gm26802
Hspa1b
Hspa1a
Fosb
Cdkn1c
Rad54b
Cdk19
Tgfb2
Sema3g
Alpl
Gm9946
aEC_large_dHET
aEC_large_dKO
aEC_stress_dHET
aEC_stress_dKO
aEC_dHET
aEC_dKO
cEC_arterial_dHET
cEC_arterial_dKO
cEC_dHET
cEC_dKO
cEC_angiogenic_dHET
cEC_angiogenic_dKO
cEC_hypoxic_dHET
cEC_hypoxic_dKO
cEC_venous_stress_dHET
cRC_venous_stress_dKO
cEC_venous_dHET
cEC_venous_dKO
vEC_activated_dHET
vEC_activated_dKO
vEC_TM_survival_dHET
vEC_TM_survival_dKO
vEC_TM_IFN_dHET
vEC_TM_IFN_dKO
vEC_TM_dHET
vEC_TM_dKO
vEC_TM_prolif_dHET
vEC_TM_prolif_dKO
Percent Expressed
25
50
75
100
Scaled Expression
2
1
0
−1
−2


***Fig. S16***

**Supplemental Figure 16.** ***Single cell RNA sequencing reveals distinct gene expression profiles for normal and tumor-associated endothelial cells in wild type and Rab27-deficient mice.*** Gene expression profiles of normal brain (BEC) and brain tumor-associated (TEC) endothelial cells in Rab27-proficient (dHET) and deficient (dKO) mice (annotations adopted from *(49)*) across BECs *versus* TECs in dHET *versus* dKO mice. Abbreviations: BECs - primary brain endothelial cells; TECs - primary tumor-derived brain endothelial cells; dHET - Rab27 double heterozygote mice; dKO - Rab27a/b double knock out; aEC - arterial endothelial cells; cEC - capillary endothelial cells; cEC_arterial - capillary endothelial cells close to the arterial branches;; cEC_venous - capillary endothelial cells close to veinous branches; vEC - venous endothelial cells; aEC_large - large artery endothelial cells; aEC_stress - arterial endothelial cells with features of stress response; cEC_angiogenic - angiogenic capillary endothelial cells; cEC_venous_stress - capillary endothelial cells close to the branches of veins with features of stress response; vEC_activated - activated venous endothelial cells; vEC_TM_survival - venous endothelial cells with features specific to the tumor microenvironment and enriched in markers of cell survival;  vEC_TM_IFN - venous endothelial cells with features specific for the tumor microenvironment and enriched in markers of inflammation; vEC_TM - venous endothelial cells with features specific for the tumor microenvironment; vEC_TM_prolif - venous endothelial cells with features specific to the tumor microenvironment and enriched in proliferation markers.

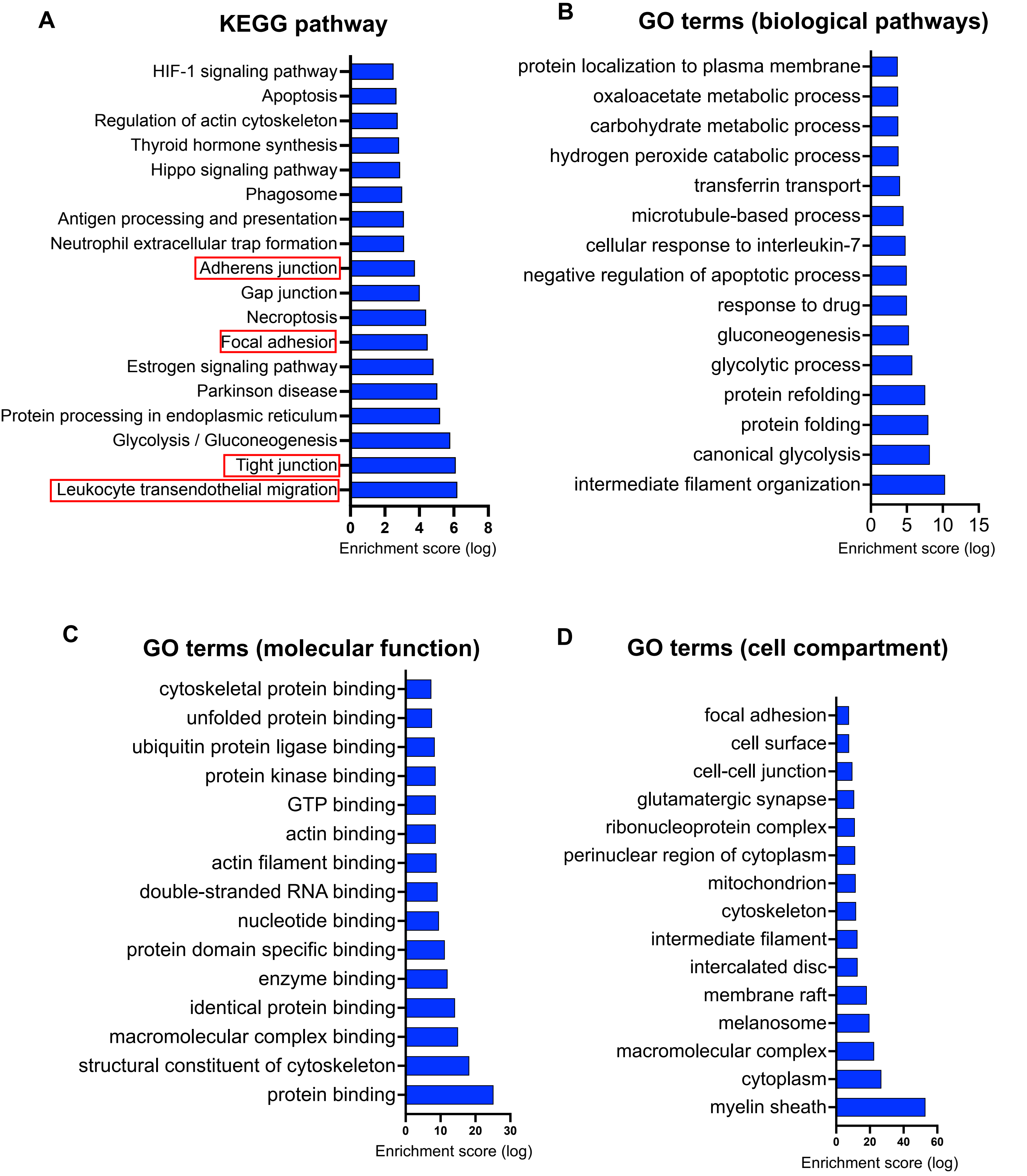


***Fig. S17***

**Supplemental Figure 17.** ***Extended analysis of normal brain endothelial cell proteome.*** **(A-D)** genes downregulated in BECs isolated from dKO relative to dHET identified KEGG pathways **(A)**, GO terms of biological pathways **(B)**, GO terms of molecular function **(C)** and GO terms of cell compartment **(D)**. The red boxes are indicative of the parallels to data (pathways enriched) presented in Fig. S11. Abbreviations: BECs - primary brain endothelial cells; dHET - Rab27a/b double heterozygote; dKO - Rab27a/b double knock out.

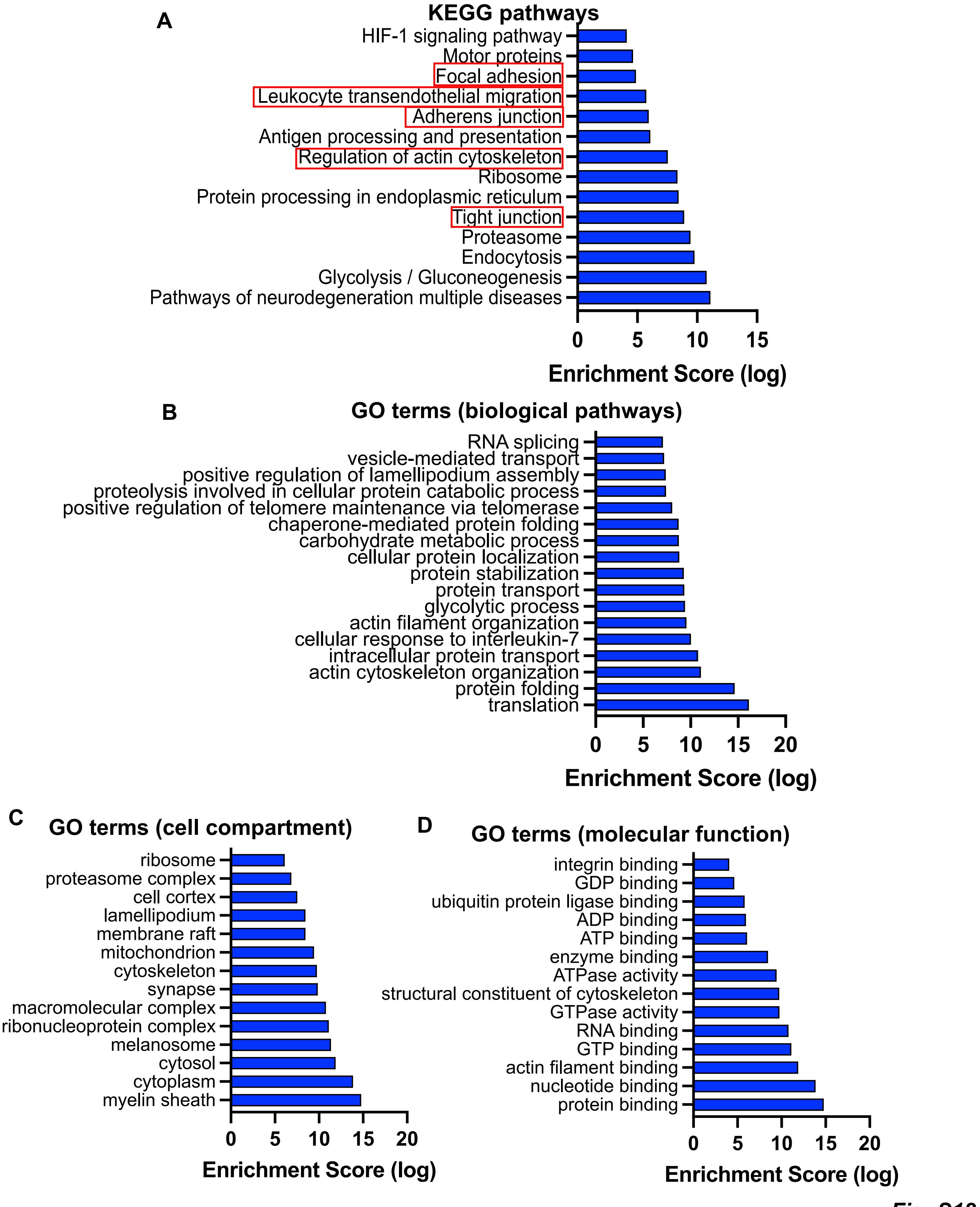


***Fig. S18***

**Supplemental Figure 18.** ***Extended proteome analysis of brain tumor-associated endothelial cells as a function of Rab27 status.*** **(A)** KEGG pathways downregulated in TECs isolated from dKO relative to dHET; **(B)** GO terms defining biological pathways differentiating TECs from dKO and dHET donors; (**C-D**) GO terms of cell compartment **(C)** or molecular function **(D)**. affected by Rab27 status. Red boxes highlight parallels with data (pathways enriched) in Supplemental Fig.10. Abbreviations: TECs - primary tumor derived brain endothelial cells; dHET - Rab27 double heterozygote; dKO - Rab27 double knock out.

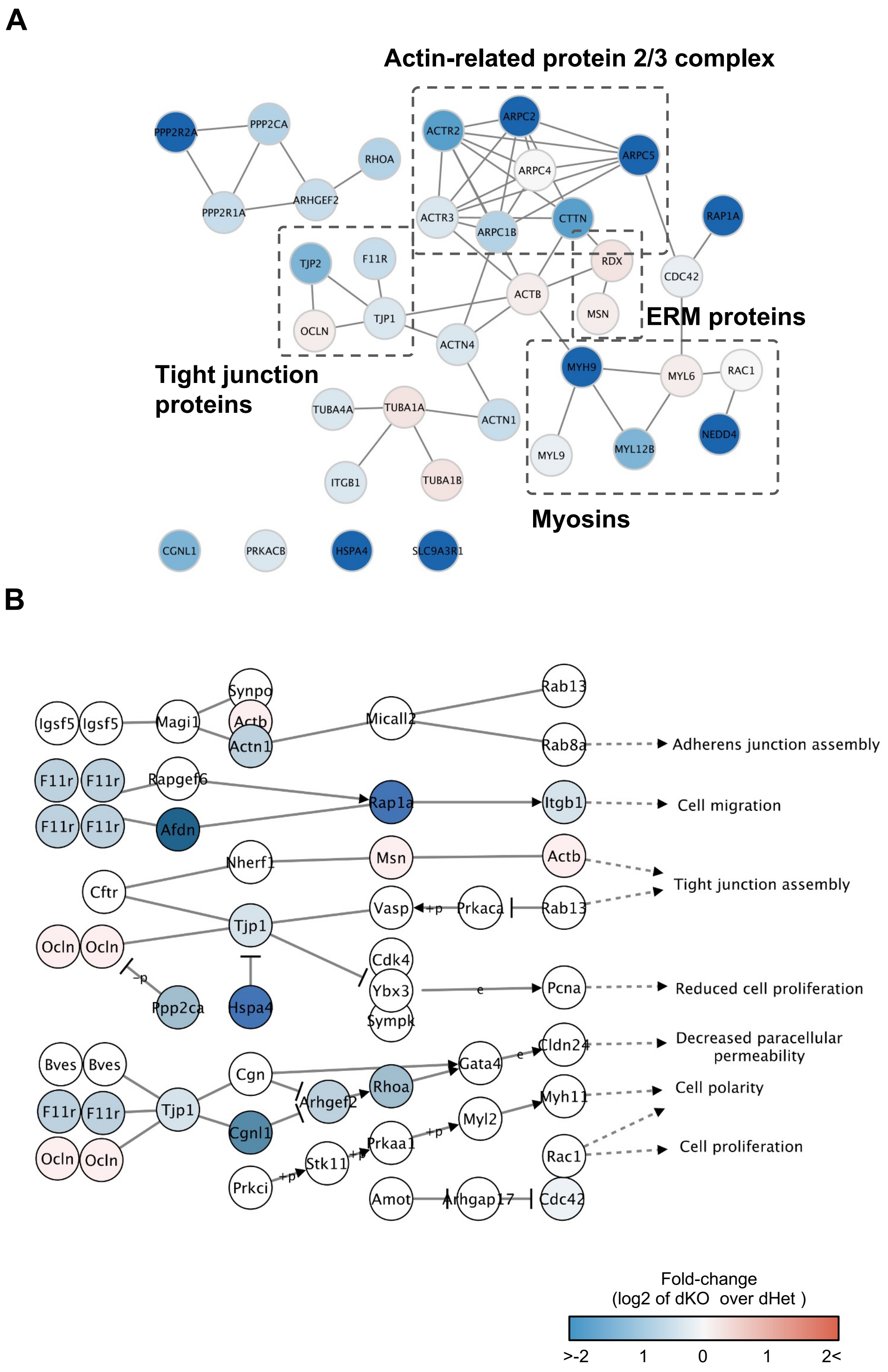


*Fig. S19*

**Supplemental Figure 19.** ***Interrelationship between constituents of KEGG pathways affected by Rab27 status and identified in the BEC proteome.*** **(A)** Network based on protein-protein interaction (BioGRID database) in dKO BECs relative to dHET. **(B)** KEGG mapping of identified proteins in the BEC proteome in dKO relative to dHET. Actins, Occudins, TJP (ZO-1 and ZO-2) are downregulated pointing to junctional proteins being affected by Rab27a/b deficiency. Abbreviations: BECs - primary brain endothelial cells; dHET - Rab27a/b double heterozygote; dKO - Rab27a/b double knock out; TJP, tight junction proteins.

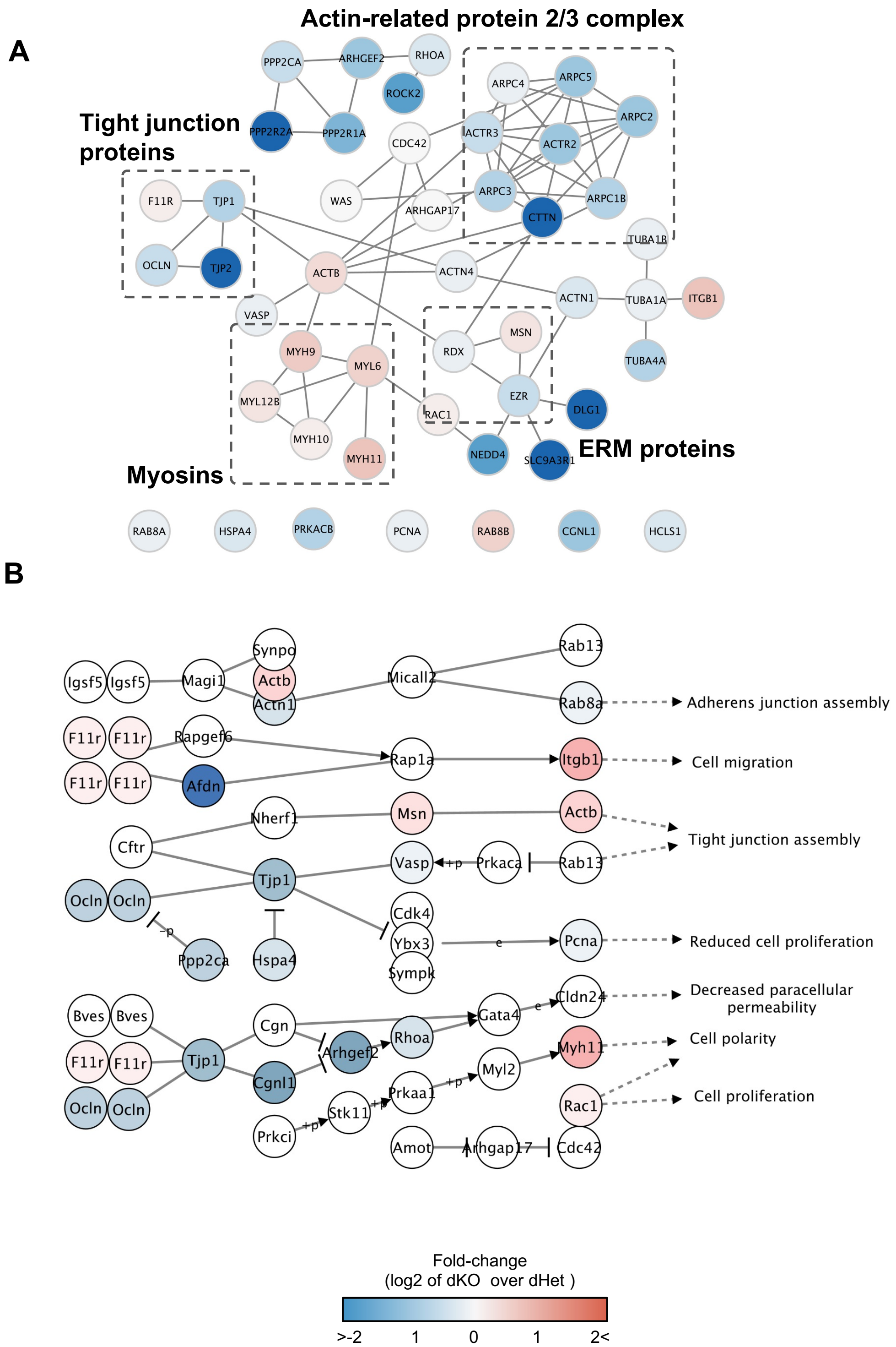


*Fig. S20*

**Supplemental Figure 20.** ***Interrelationships between constituents of KEGG pathways affected by Rab27 status and identified in the TEC proteome.*** **(A)** Network based on protein-protein interaction (BioGRID database) in dKO TECs relative to dHET TECs. **(B)** KEGG mapping of identified proteins in the TEC proteome of dKO cells relative to their dHET counterparts . Actins, Occudins, TJP (ZO-1 and ZO-2) are downregulated pointing to their levels beong affected by Rab27 deficiency. Abbreviations: TECs - primary tumor-derived brain endothelial cells; dHET - Rab27 double heterozygote; dKO - Rab27 double knock out; TJP, tight junction proteins.

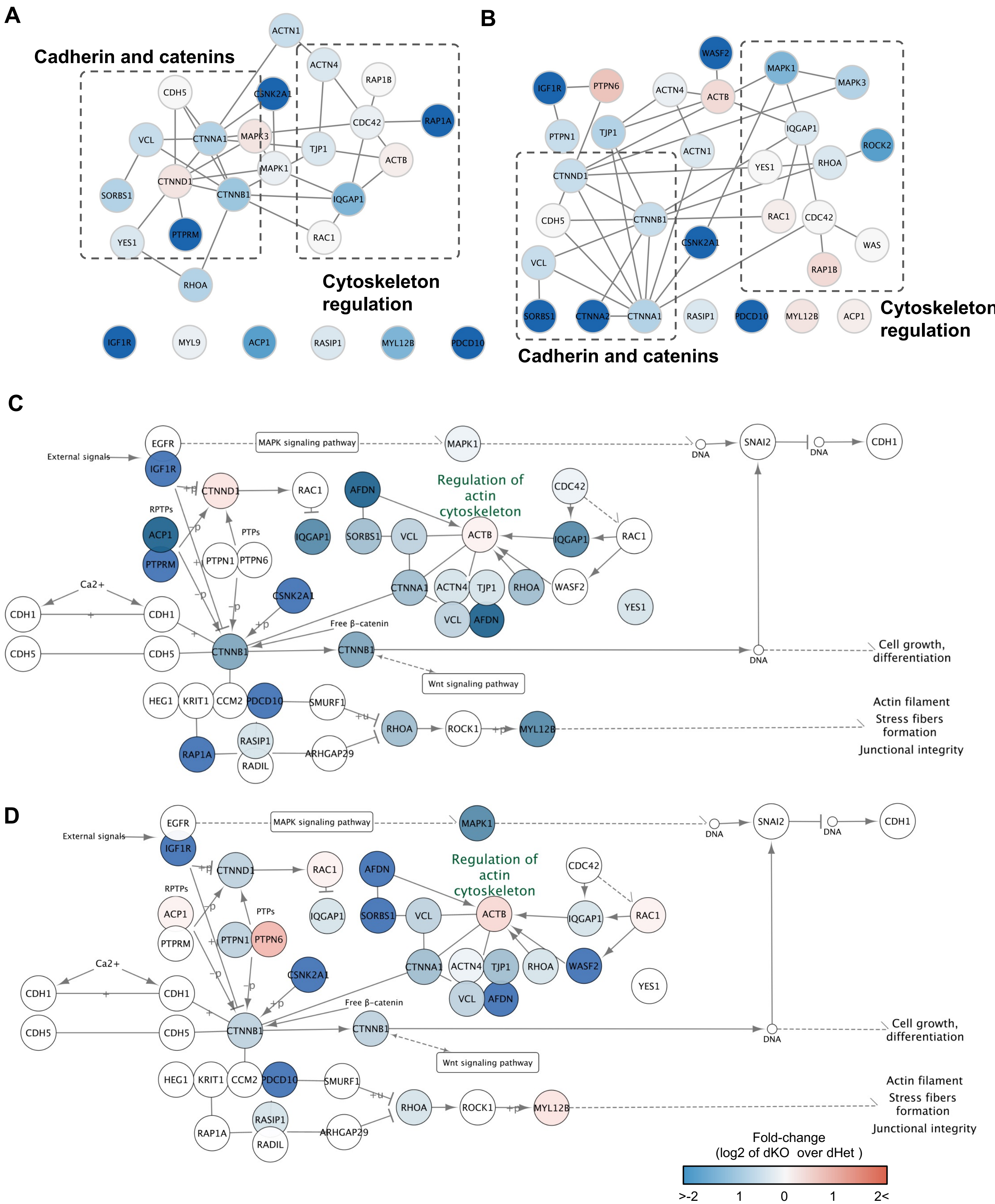


***Fig. S21***

**Supplemental Figure 21.** ***Representation of adherens junctional complexes in the KEGG pathway analysis of brain endothelial proteomes.*** **(A, B)** Molecular network based on protein-protein interaction (BioGRID database) in BECs **(A)** and TECs **(B);** Color-coded changes in expression depict fold change between dKO relative to dHET donors. **(C,D)** KEGG mapping of identified proteins in the endothelial cell proteomes of BECs **(C)** and TECs **(D);** Comparison of cells from dKO relative to dHET donors. Abbreviations: BECs - primary brain endothelial cells; TECs - primary tumor derived brain endothelial cells; dHET, - Rab27a/b double heterozygote; dKO - Rab27a/b double knock out.

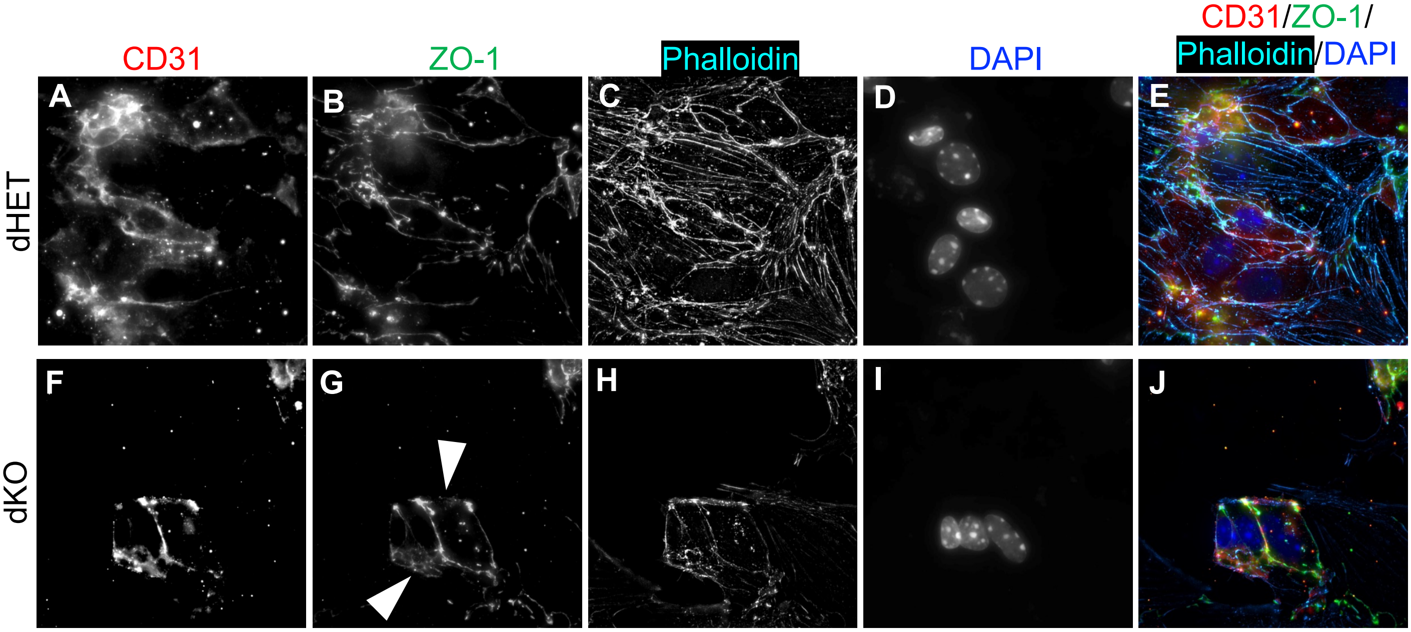


***Fig. S22***

**Supplemental Figure 22.** ***Actin disorganization and loss of tight junctions in endothelial cells deficient for Rab27.*** **(A-J)** Immunofluorescent staining for CD31 (red), ZO-1 (green), phalloidin (cyan) and DAPI/chromatin (blue) in dHET BECs **(A-E)**, and dKO BECs **(F-J)**. Abbreviations: BECs - primary brain endothelial cells; dHET - Rab27a/b double heterozygote; dKO - Rab27a/b double knock out. Images were obtained using the super-resolution microscope at 63X and processed by structured illumination calculation, followed by maximum intensity projection to combine the z-stacks.

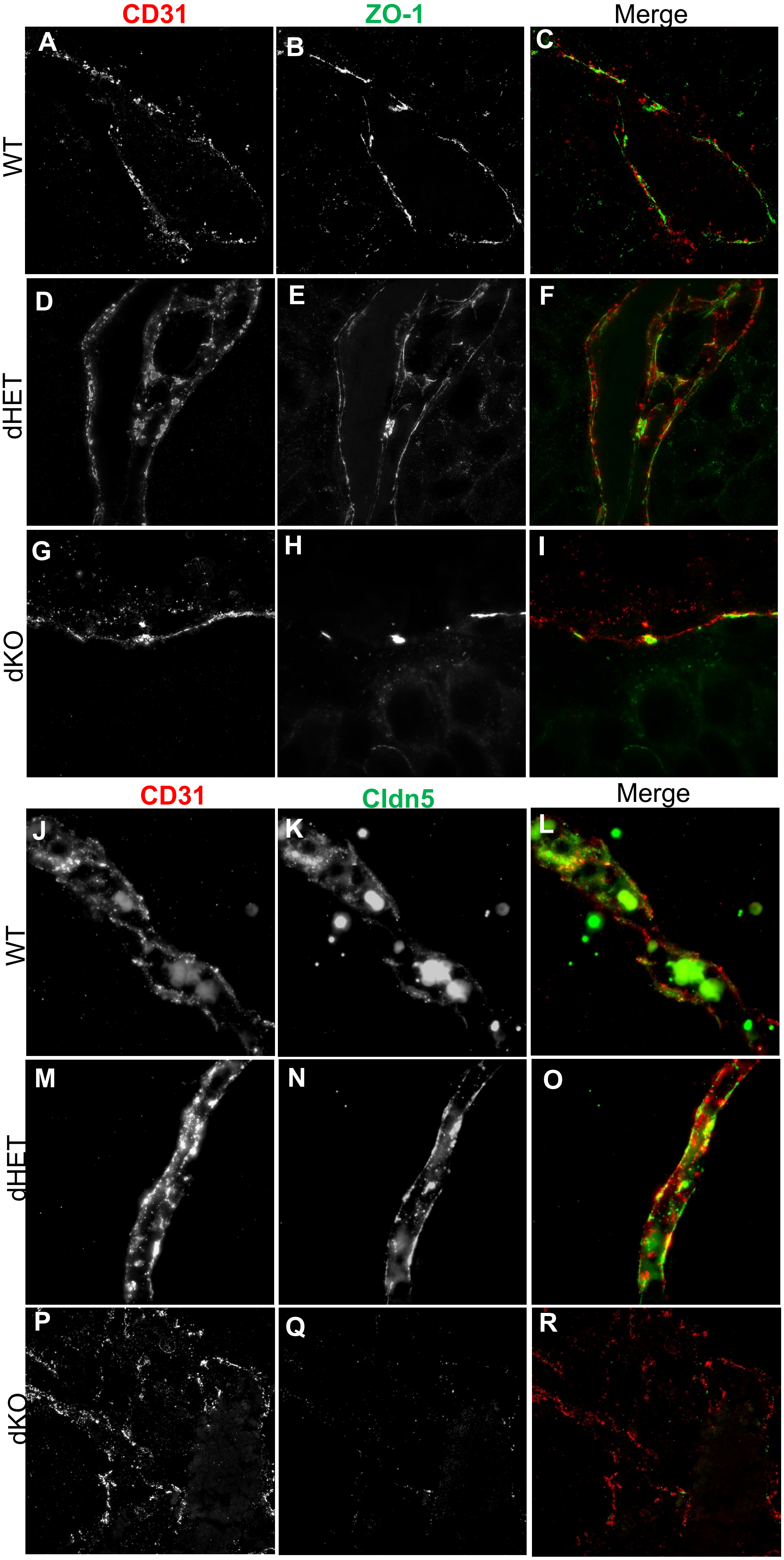
CD31
ZO-1
Merge
A
B
C
WT
D
E
F
dHET
G
H
I
dKO
CD31
Cldn5
Merge
J
K
L
WT
M
N
O
dHET
P
Q
R
dKO

*Fig.S23*

**Supplemental Figure 23.** ***Loss of tight junctions in tumor-associated vascular endothelial cells deficient for Rab27.*** **(A-J)** Tissue sections from intracranial GL261 tumors stained for CD31 (red) and ZO-1 (green) in different mouse hosts, including WT mice **(A-C)**, dHET mice **(D-F)** and dKO mice **(G-I)**. **(J-R)** Colocalization of CD31 (red) and cldn5 (green) expression in blood vessels of GL261 brain tumors from different hosts, including WT mice **(J-L)**, dHET mice **(M-O)** and dKO mice **(P-R)**. Abbreviations: WT - wild type mice (C57bl/6); dHET - Rab27a/b double heterozygote mice; dKO - Rab27a/b double knock out mice. Images were obtained using the super-resolution microscope at 63X and processed by structured illumination calculation, followed by maximum intensity projection to combine the z-stacks.

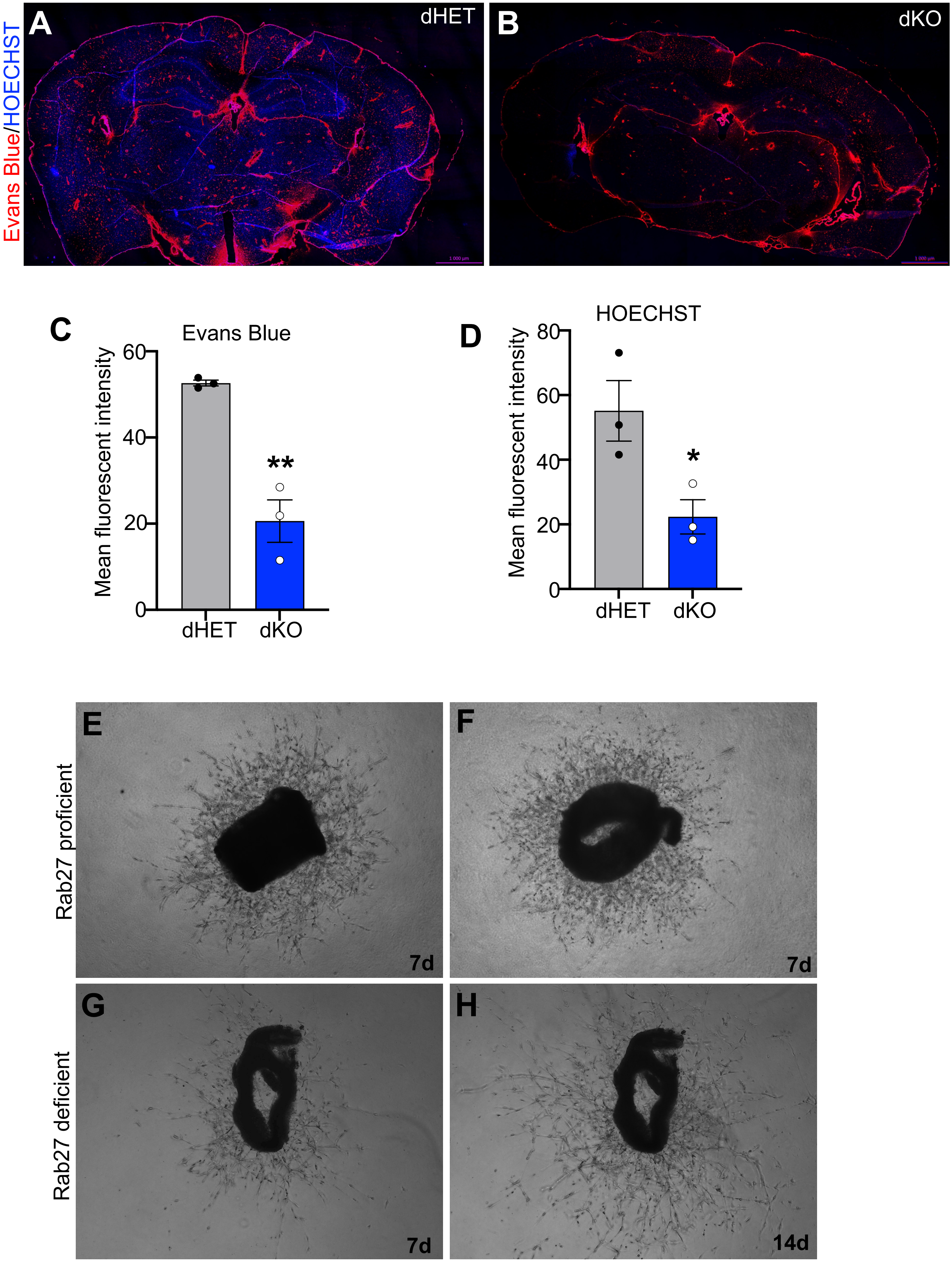
Evans Blue/HOECHST
A
dHET
B
dKO
C
Evans Blue
Mean fluorescent intensity
60
40
20
0
**
dHET
dKO
D
HOECHST
Mean fluorescent intensity
80
60
40
20
0
*
dHET
dKO
E
F
G
H
Rab27 proficient
Rab27 deficient
7d
7d
7d
14d

*Fig.S24*

**Supplemental Figure 24.** ***Rab27-deficiency is associated with reduced permeability of the brain vasculature and retarded angiogenic responses ex vivo.*** **(A,B)** Confocal images of the brains of dHET **(A)** and dKO **(B)** animals perfused with Evans blue (red) and HOECHST (blue). **(C,D)** Quantification of the mean fluorescence intensities of the brains of dHET **(C)** and dKO **(D). (E-H)** Aortic ring explants cultured for 7 days **(E-G)** and 14 days **(H)** from WT mice **(E)**, dHET mice (**F**) or dKO mice **(G, H).** The extent of endothelial outgrowths from aortic explants is initially diminished in the context of Rab27 deficiency relative to control, but endothelial growth continues through 14 days in culture. Abbreviations: WT - wild type (C57bl/6); dHET - Rab27a/b double heterozygote; dKO - Rab27a/b double knock out; Not significant (ns); $P < 0.05$ (*); $P < 0.01$ (**); $P < 0.001$ (***) and $P < 0.0001$ (****).

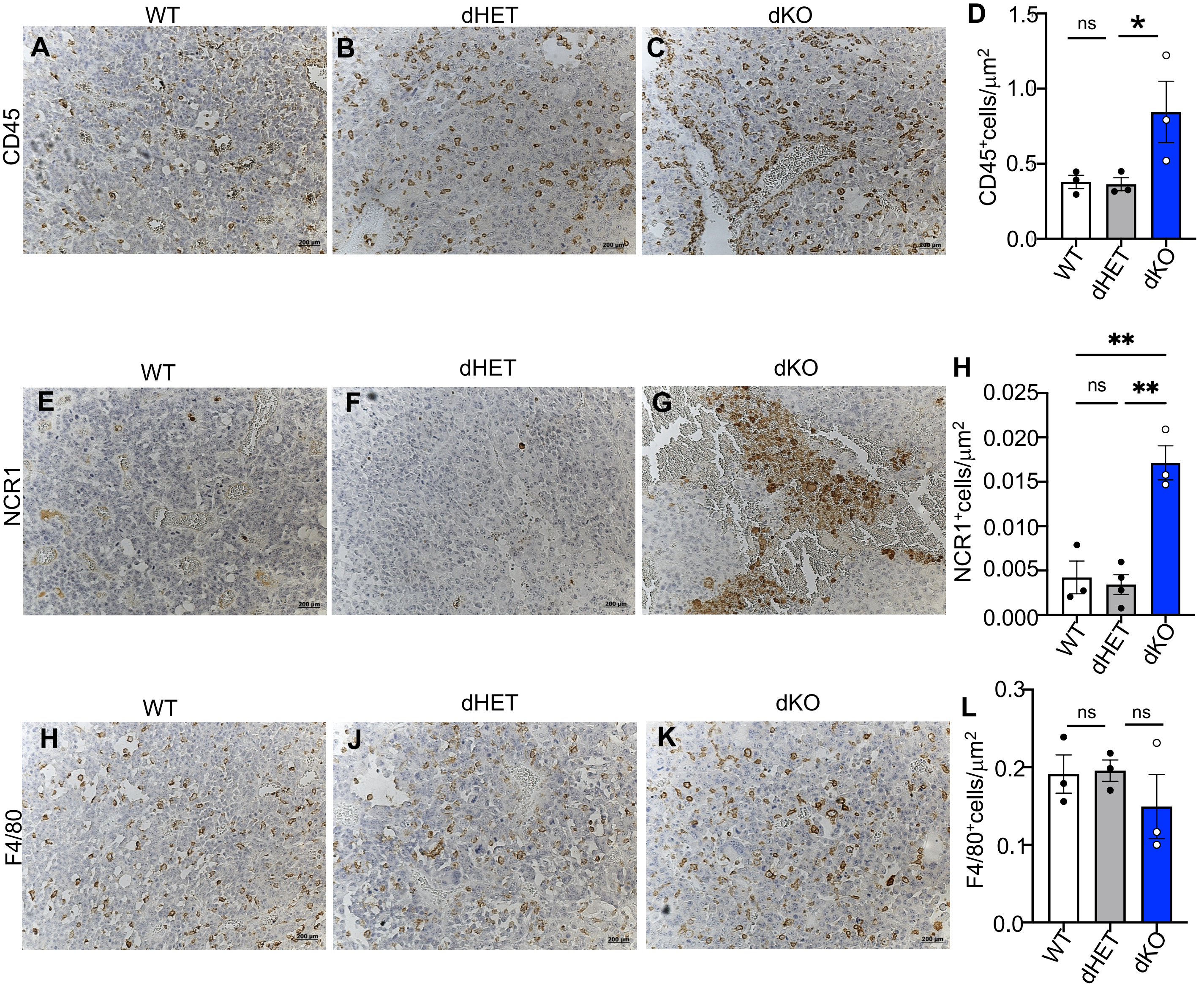


***Fig. S25***

**Supplemental Fig. 25:** ***Immune cell infiltration into the GL261 brain tumor microenvironment is increased by Rab27 deficiency.*** **(A-C)** Staining of GL261 brain tumor sections for pan-leucocytic marker, CD45 (DAB/immunohistochemistry) in different mouse hosts, including WT mice **(A)**, dHET mice **(B)** and dKO mice **(C)**. **(D)** Quantification of $CD45^+$ cells in the tumor microenvironment of indicated hosts: WT (white), dHET (grey) or dKO mice (blue). **(E-G)** Staining of GL261 brain tumors for NK marker, NCR1 (DAB/immunohistochemistry) in WT **(E)**, dHET **(F)** and dKO mice **(G)**. **(H)** Quantification of $NCR1^+$ cells in tumors of mice with indicated genotypes: WT (white), dHET (grey) and dKO (blue). **(I-K)** Staining macrophage marker, F4/80 in GL261 brain tumors inoculated into WT **(I)**, dHET **(J)** or dKO mice **(K)**. **(L)** Quantification of $F4/80^+$ cells in GL261 brain tumors of WT (white), dHET (grey) and dKO mice (blue). Abbreviations: WT - wild type (C57bl/6); dHET - Rab27a/b double heterozygote; dKO - Rab27a/b double knock out; Not significant (ns); $P < 0.05$ (*); $P < 0.01$ (**); $P < 0.001$ (***) and $P < 0.0001$ (****).

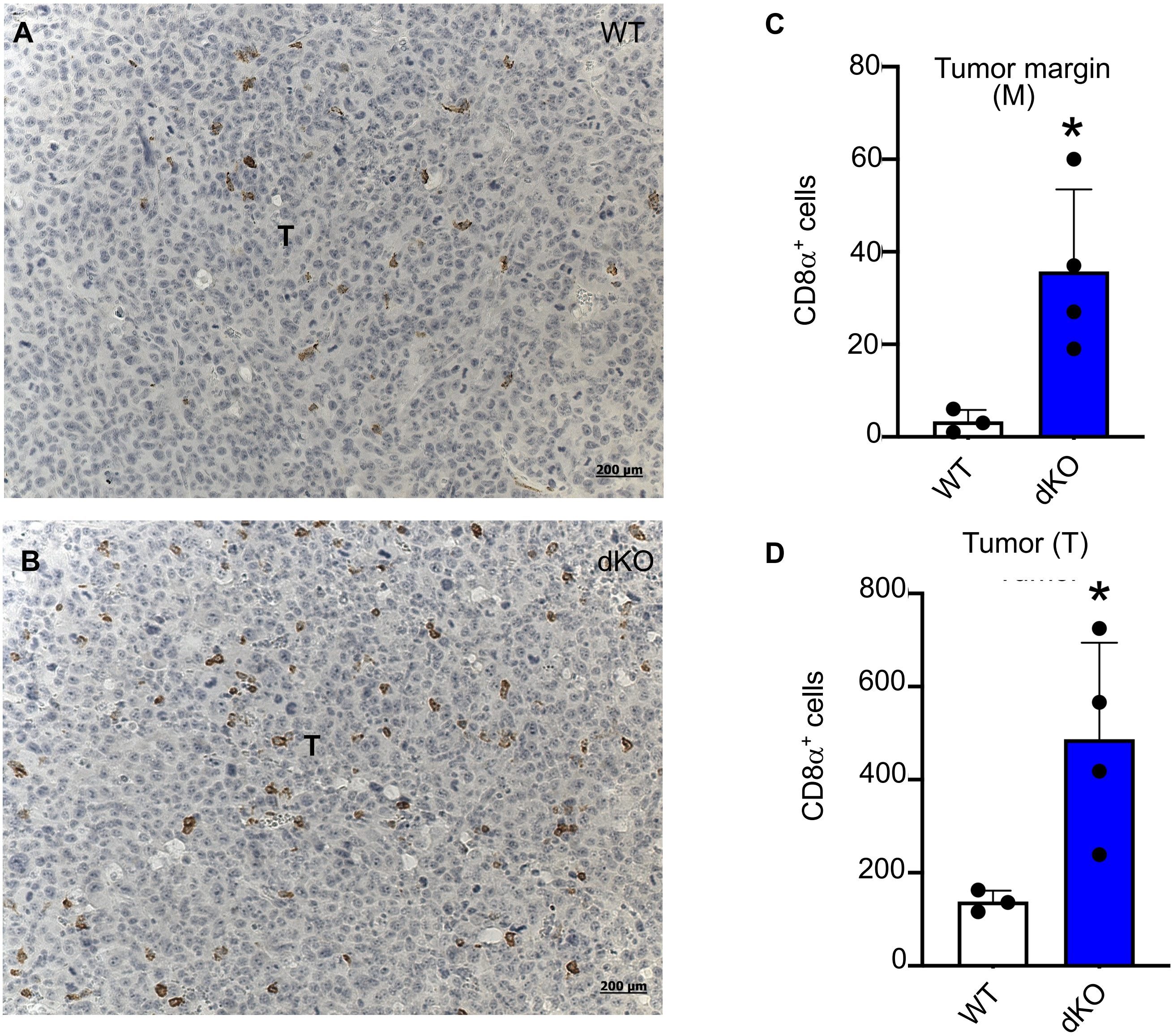


***Fig. S26***

**Supplemental Figure 26.** ***Effector CD8α⁺ cell infiltration into Rab27-deficient E0771 brain tumor microenvironment.*** **(A-B)** CD8α immunohistochemistry of intracranial E0771 tumors in WT **(A)**, or dKO mice **(B)**. **(C, D)** Quantification of $CD8\alpha^+$ cells in the E0771 brain tumor microenvironment of WT (white) and dKO (blue) mice in either tumor margin (M; **C**) or tumor core (T; **D**). Abbreviations: WT - wild type mice (C57bl/6); dKO - Rab27a/b double knock out; M - tumor margin; T – tumor core; Not significant (ns); $P < 0.05$ (*); $P < 0.01$ (**); $P < 0.001$ (***) and $P < 0.0001$ (****).

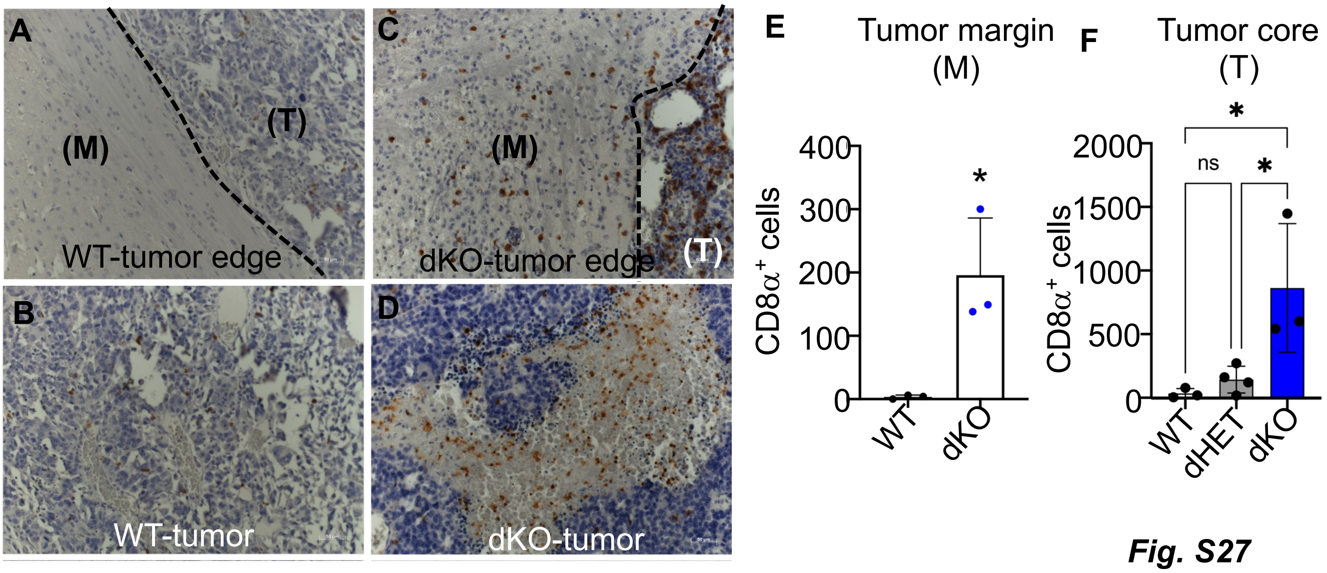


***Fig. S27***

**Supplemental Figure 27.** ***Effector CD8α$^+$ cell infiltration into Rab27-deficient GL261 tumor microenvironment.*** **(A-D)** Staining for CD8α (DAB/immunohistochemistry) in GL261 brain tumor tissues corresponding to tumor edge in WT mice **(A)**, tumor core in WT mice **(B)**, tumor edge in dKO mice **(C)**, tumor core in dKO mice **(D)**. **(E,F)** Quantification of the indicated CD8α$^+$ cell infiltrates in brain tumors of WT mice (white), dHET mice (grey) or dKO mice (blue), either measured at the tumor edge **(E)** or tumor core **(F)**. Abbreviations: WT - wild type mice (C57bl/6); dHET - Rab27a/b - double heterozygote; dKO - Rab27a/b - double knock out; Not significant (ns); $P < 0.05$ (*); $P < 0.01$ (**); $P < 0.001$ (***) and $P < 0.0001$ (****).

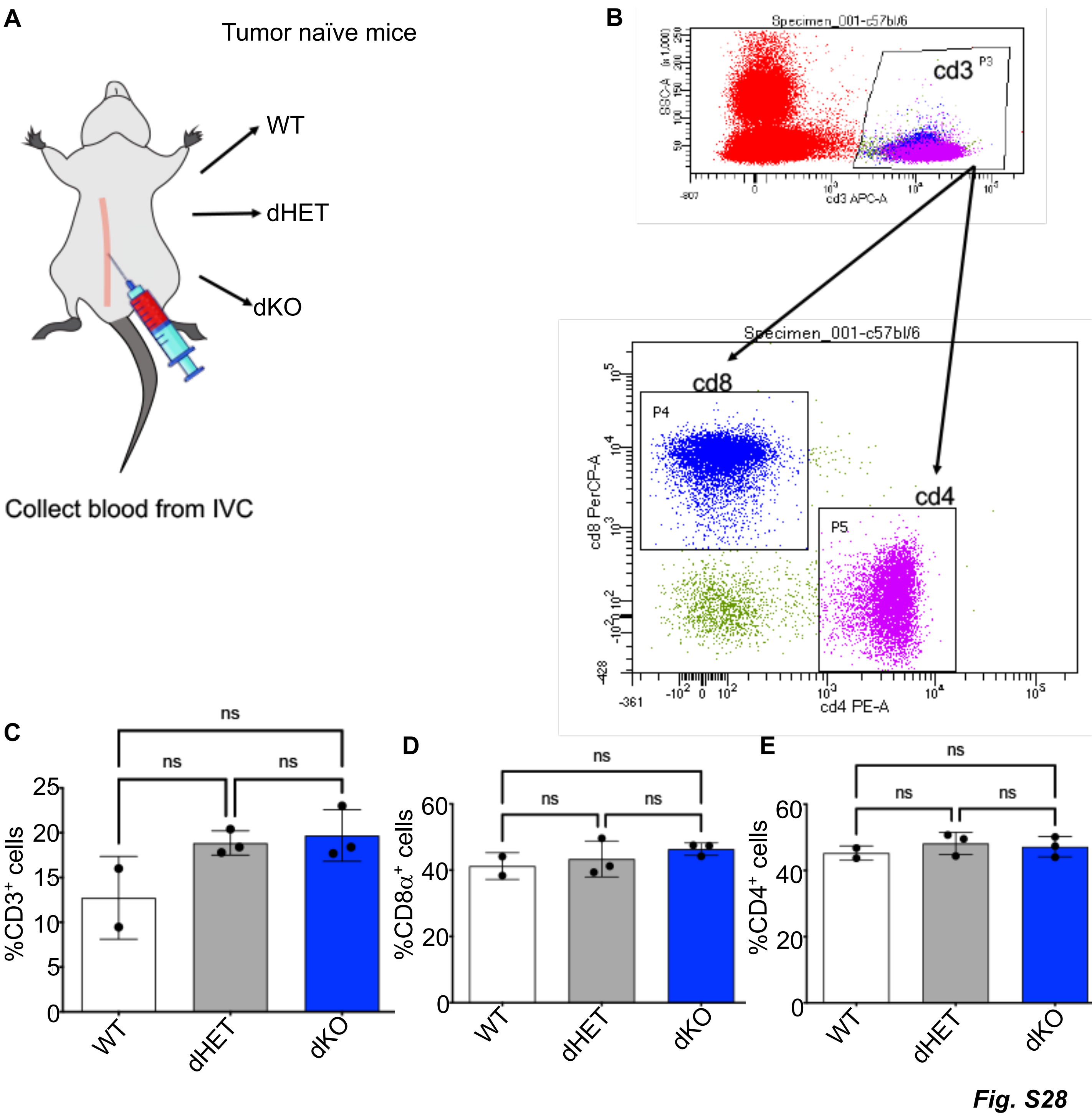


**Supplemental Fig. 28.** ***T-cells in systemic circulation of Rab27-proficient and -deficient mice.*** **(A)** Peripheral blood collection from inferior vena cava (IVC) of mice. **(B)**, Gating strategy to obtain $CD3^+$, $CD8\alpha^+$ and $CD4^+$ cell populations. **(C-E)** Quantification (% of total while blood cell count) of indicated lymphoid cell populations in peripheral blood of mice with different genotypes, including WT (white), dHET (grey) and dKO (blue) mice. Samples were analyzed for the content of $CD3^+$ **(C)**, $CD8\alpha^+$ **(D)**, and $CD4^+$ cells in the circulation **(E)**. Abbreviations: WT - wild type mice (C57bl/6); dHET - Rab27a/b double heterozygote; dKO - Rab27 double knock out mice; ns - not significant.

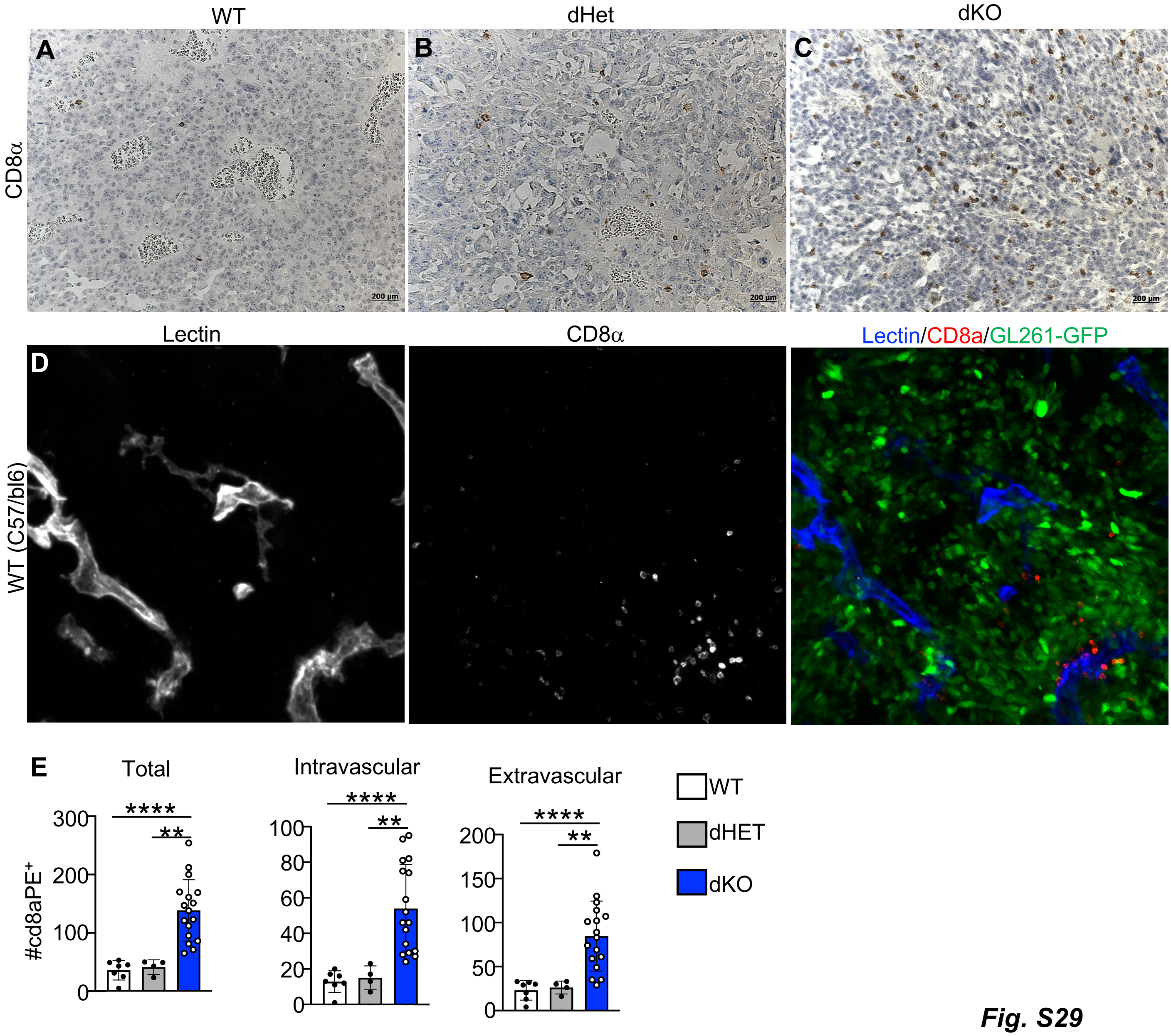


*Fig. S29*

**Supplemental Figure 29.** ***Differential T-cell infiltration into brain tumor tissues in mice proficient and deficient for Rab27.*** **(A-C)** Immunohistochemical detection of CD8α$^{+}$ T cells in GL261 brain tumors in WT (**A**), dHET (**B**) and dKO (**C**) recipient mice. (D) Blood vessel perfusion-staining superimposed with T cell staining of GL261 brain tumor tissues of WT recipient mice using lectin (blue), CD8α-PE (red) and GL261-GFP (green). Thick sections of tumor tissues (250µm in thickness) were generated using vibratome and the respective fluorescent images were collected using confocal microscopy. **(E)** Quantification of total CD8α$^{+}$ cell count per field (left panel), CD8α$^{+}$ cells within blood vessel lumen (middle panel) and extravascular CD8α$^{+}$ cells (right panel) in mice with different genotypes, including WT (white), dHET (grey) and dKO (blue) tumor recipients. Abbreviations: WT – wild type mice (C57bl/6); dHET – double Rab27a/b heterozygotes; dKO – double Rab27a/b knock-outs; Not significant (ns); $P < 0.05$ (*); $P < 0.01$ (**); $P < 0.001$ (***) and $P < 0.0001$ (****).

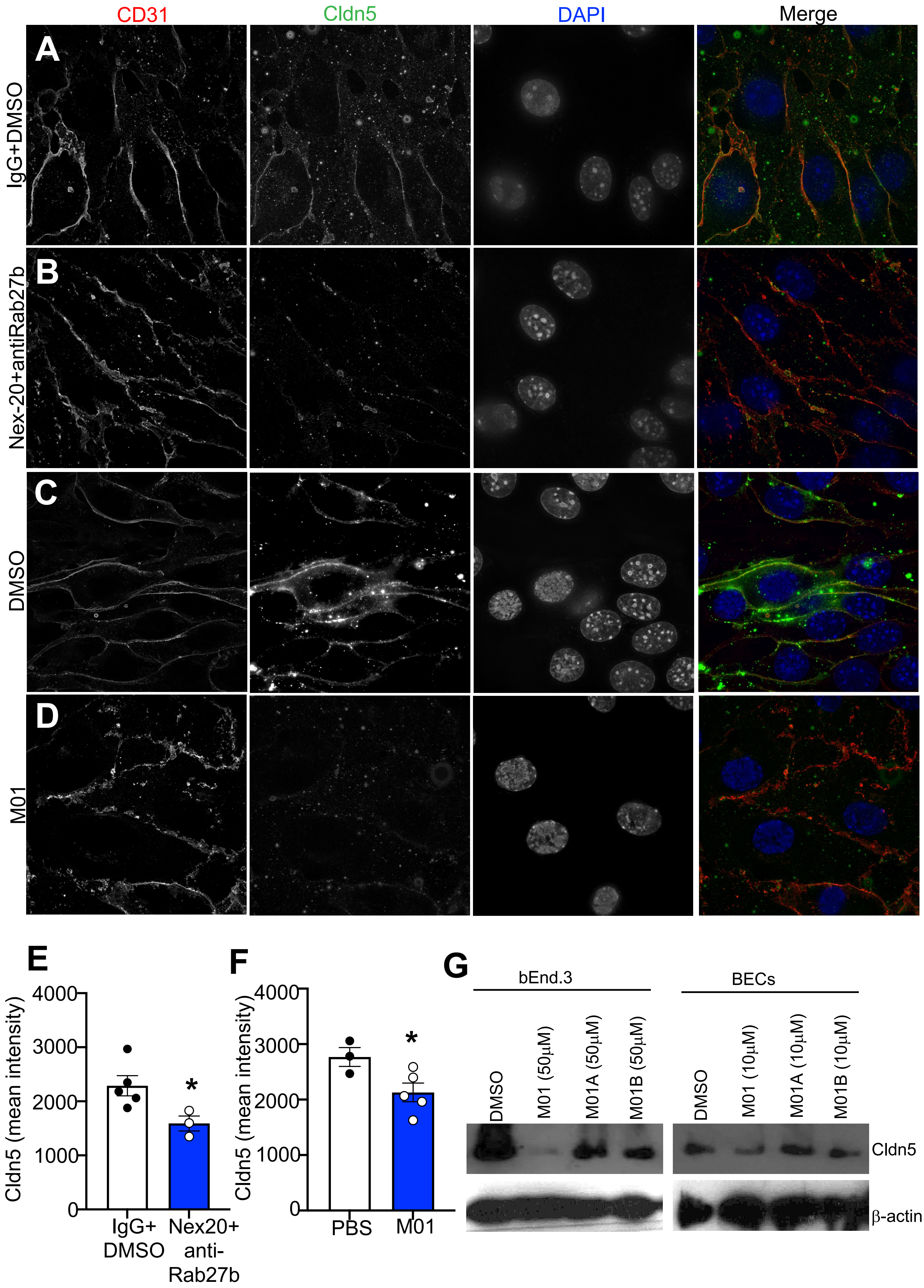
CD31
Cldn5
DAPI
Merge
A
IgG+DMSO
B
Nex-20+antiRab27b
C
DMSO
D
M01
E
Cldn5 (mean intensity)
4000
3000
2000
1000
0
*
IgG+ DMSO
Nex20+ anti-Rab27b
F
Cldn5 (mean intensity)
4000
3000
2000
1000
0
*
PBS
M01
G
bEnd.3
BECs
DMSO
M01 (50μM)
M01A (50μM)
M01B (50μM)
DMSO
M01 (10μM)
M01A (10μM)
M01B (10μM)
Cldn5
β-actin

*Fig. S30*

**Supplemental Figure 30.** ***M01 inhibits claudin 5 expression and assembly during monolayer formation by primary brain endothelial cells.*** **(A-D)** Immunofluorescent staining visualising the expression of CD31 (red), claudin 5 (green) and DAPI (blue) in primary BECs treated with IgG+DMSO **(A)**, Nexinhib20+anti-Rab27b antibody **(B)**, DMSO **(C)**, and 10μM of claudin 5 inhibitor, M01 **(D)**. **(E,F)** Quantifications of claudin 5 mean immunostaining intensity in BECs treated with IgG+DMSO and Nexinhib20 **(E)** and claudin 5 inhibitor (M01 versus control) used to mimic tight junction defects in Rab27-deficient BECs **(F)**. **(G)** Western blot documenting low expression of claudin 5 in both bEND.3 and BEC endothelial cells following exposure to M01. Abbreviations: Cldn5 – claudin 5; BECs – primary mouse brain endothelial cells; bEnd.3 – mouse brain endothelial cell line; Not significant (ns); $P < 0.05$ (*); $P < 0.01$ (**); $P < 0.001$ (***) and $P < 0.0001$ (****).

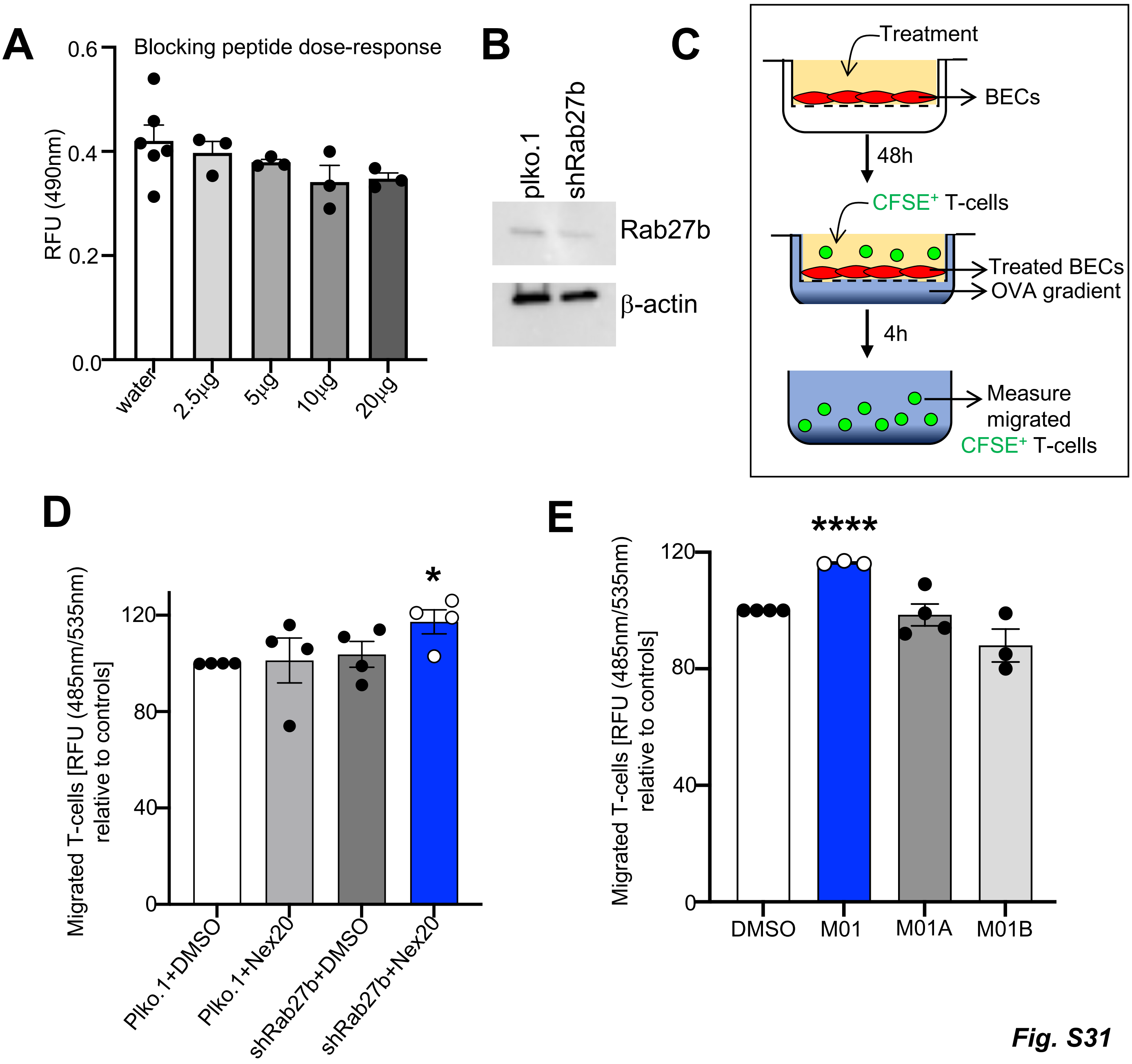


**Supplemental Figure 31.** ***Rab27 and claudin 5 inhibitors facilitate T-cell migration across endothelial cell monolayers*** **(A)** MTS cell survival assay to establish a non-toxic dose of the anti-Rab27b antibody blocking peptide. **(B)** Silencing of Rab27b using shRNA - 50% reduction in expression observed in BECs treated with shRNA versus empty vector (plko.1). **(C)** Schematic representing the experimental design to study T-cell trans-endothelial migration *in vitro*. **(D)** Impact on trans-endothelial migration of CFSE-labelled T cells following inhibition of Rab27a activity (Nexinhib20) and/or expression of Rab27b (shRNA) in endothelial cells; **(E)** Increase in trans-endotelial migration of CFSE-labelled T cells following inhibition of endothelial claudin 5 (M01) relative to controls, including vehicle (DMSO and inactive M01 isoforms (M01A and M01B); Not significant (ns); $P < 0.05$ (*); $P < 0.01$ (**); $P < 0.001$ (***) and $P < 0.0001$ (****).

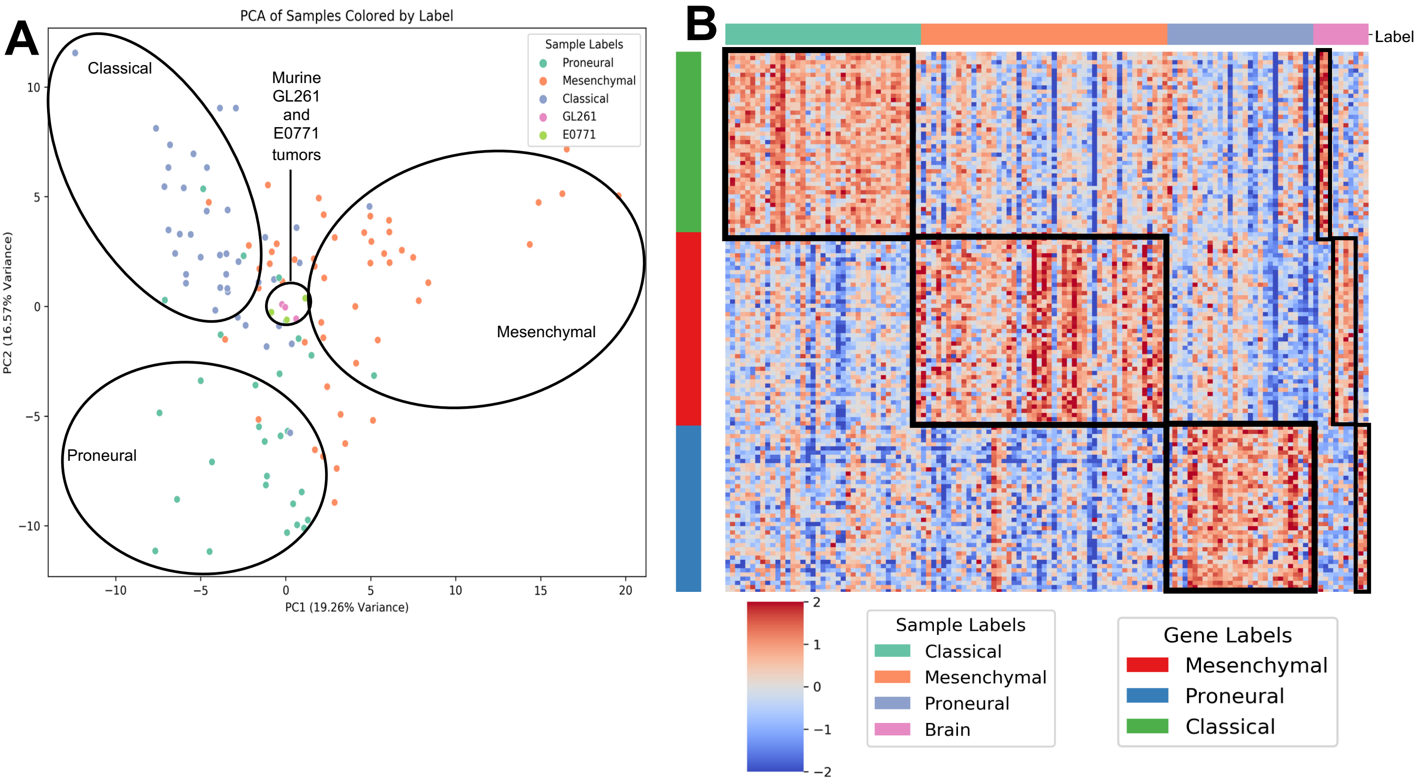


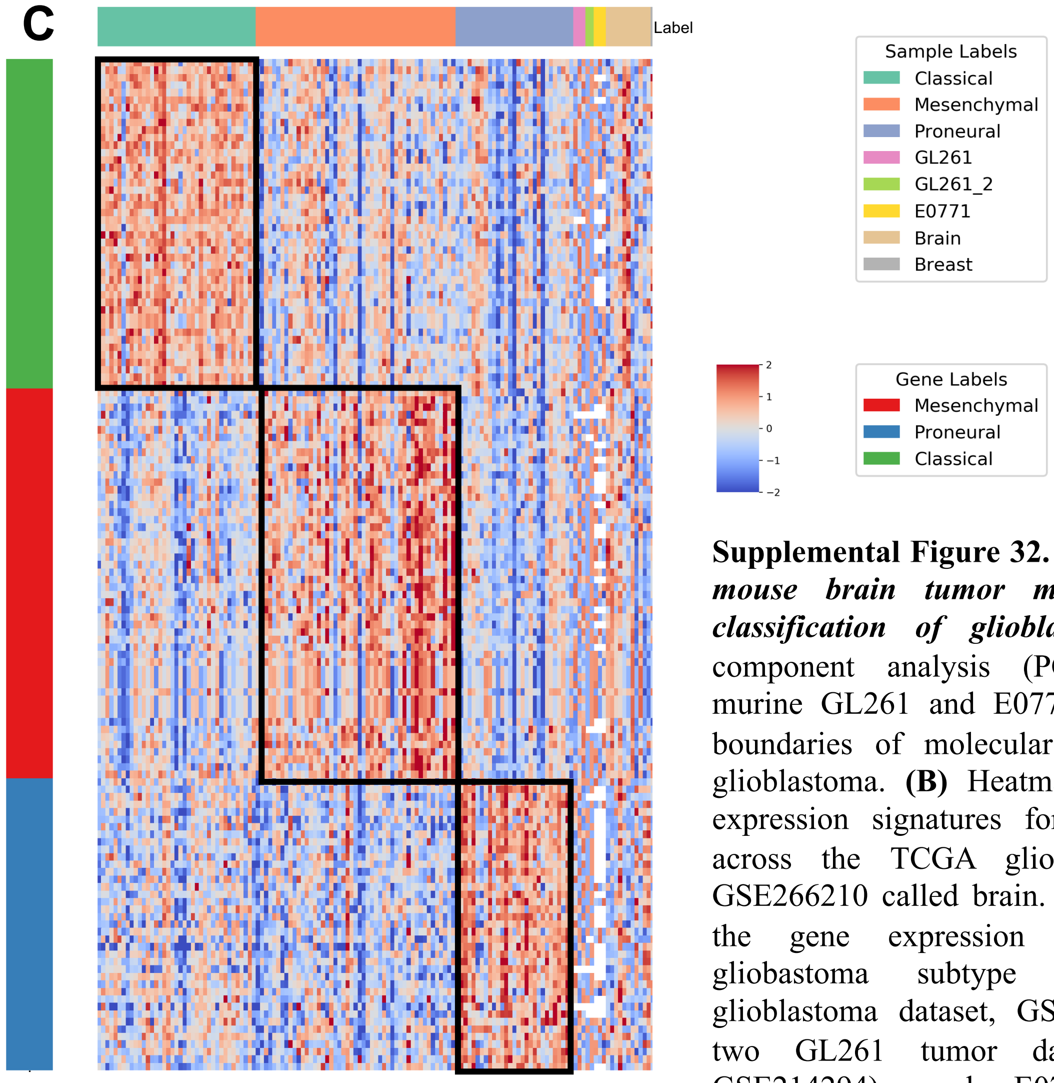


***Fig. S32***

**Supplemental Figure 32.** ***The correspondence of mouse brain tumor models and molecular classification of glioblastoma.*** **(A)** Principal component analysis (PCA) plot positioning murine GL261 and E0771 tumors between the boundaries of molecular subgroups of human glioblastoma. **(B)** Heatmap based on the gene expression signatures for each tumor subtype across the TCGA glioblastoma dataset and GSE266210 called brain. **(C)** Heatmap based on the gene expression signatures for each gliobastoma subtype across the TCGA glioblastoma dataset, GSE266210 called brain, two GL261 tumor databases (GSE178371, GSE214294), and E0771 tumor database (GSE244138 ).

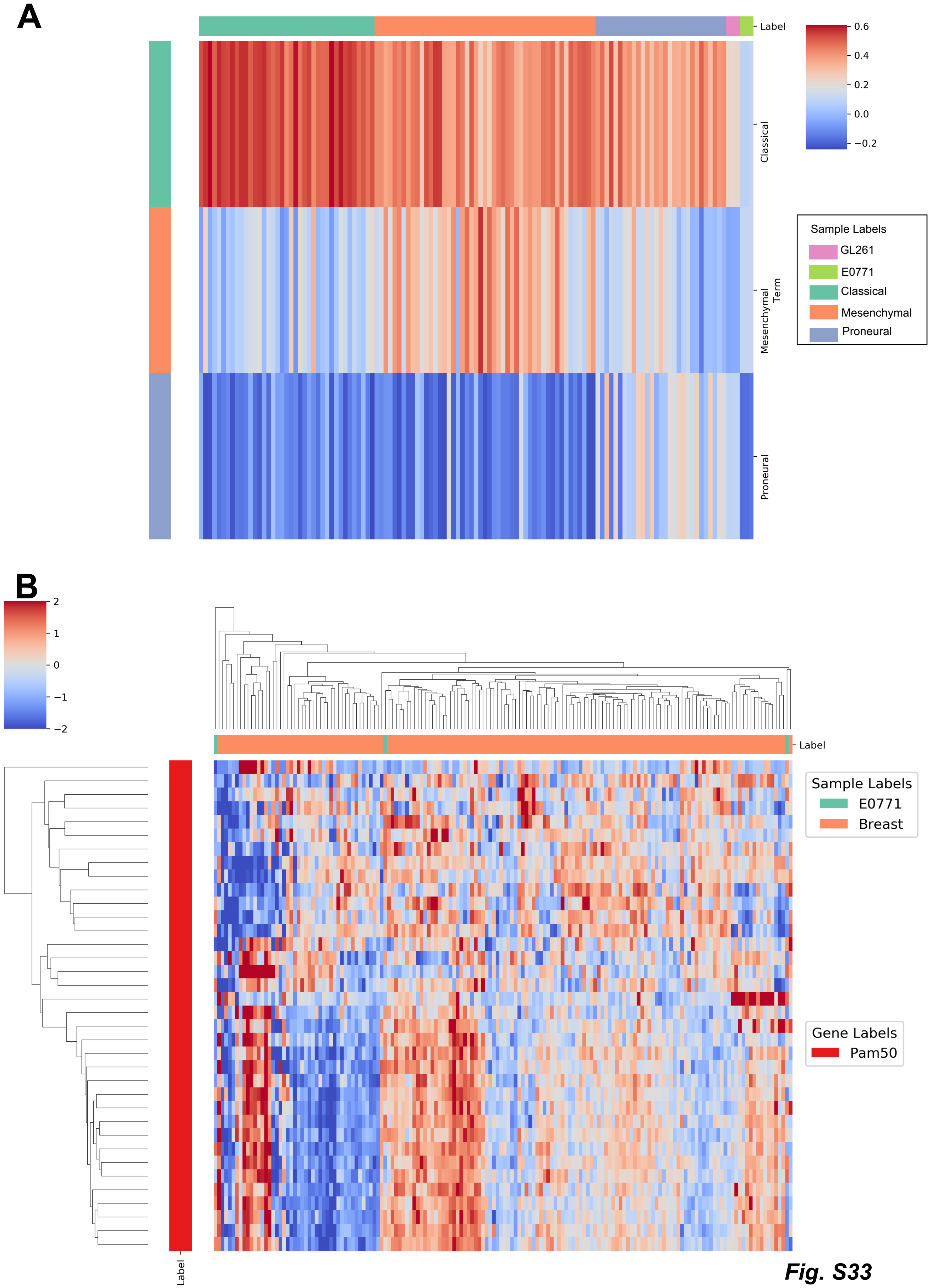


*Fig. S33*

**Supplemental Figure 33.** ***Comparison of mouse models and molecular classifiers of human brain and breast cancers.*** **(A)** ssGSEA analysis of gliobastoma subtypes across TCGA dataset superimposed with mouse GL261 tumor database (GSE178371), and E0771 tumor database (GSE244138). **(B)** Heatmap based on the gene expression signatures for PAM50 and E0771 mouse tumor model (GSE244138).

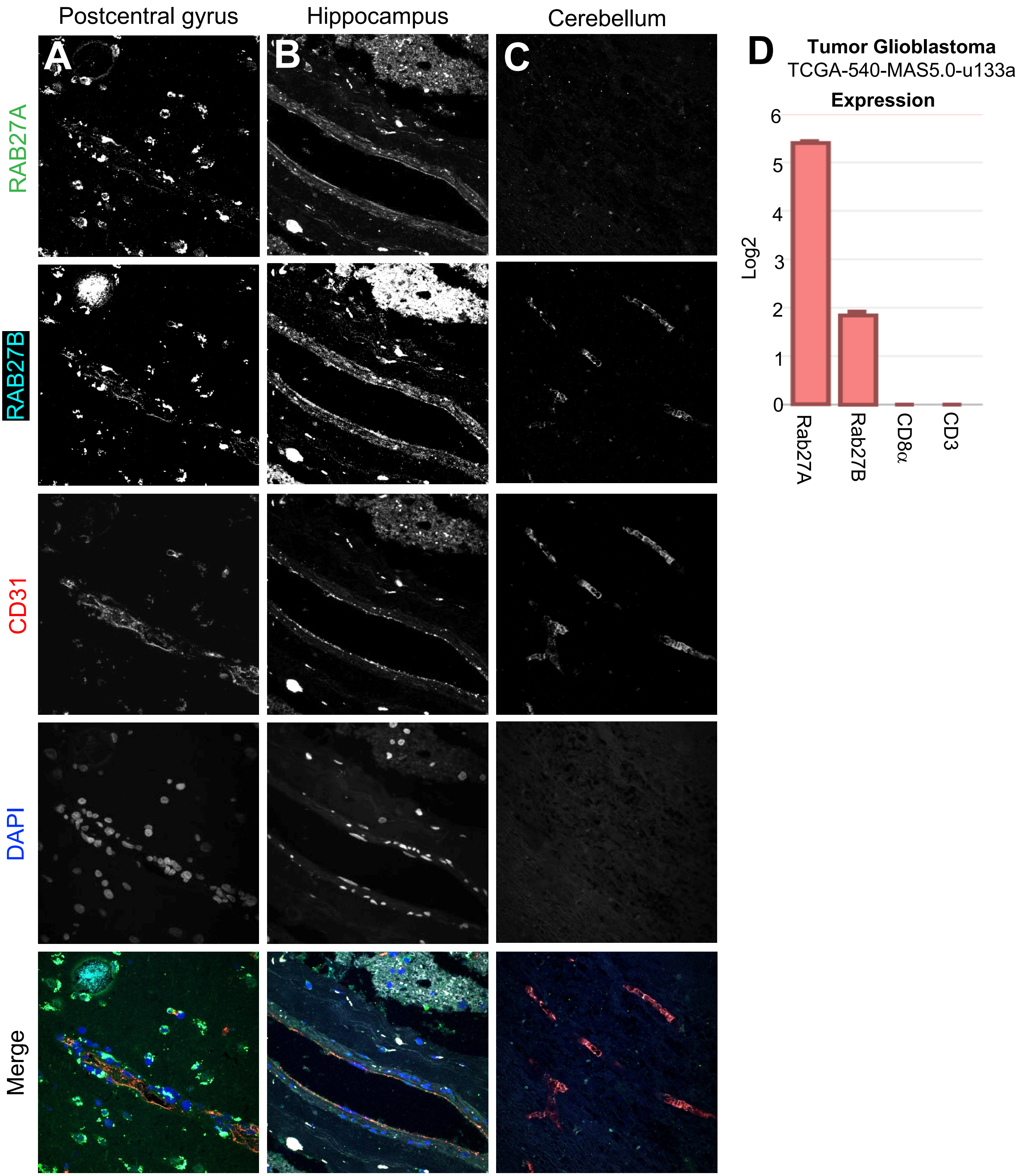


*Fig. S34*

**Supplemental Figure 34.** ***Rab27 expression in patient brain tumor tissue samples.*** **(A-C)** Human brain tissues express Rab27a and Rab27b in CD31$^{+}$ vascular cells. Tissues acquired from Biochain for postcentral gyrus **(A)**, hippocampus **(B)** and cerebellum **(C)** were stained for RAB27A, RAB27B, CD31 and nuclei (DAPI). **(D)** TCGA expression analysis of RAB27A, RAB27B, CD8a, and CD3 across 540 patient samples of glioblastoma.

211906

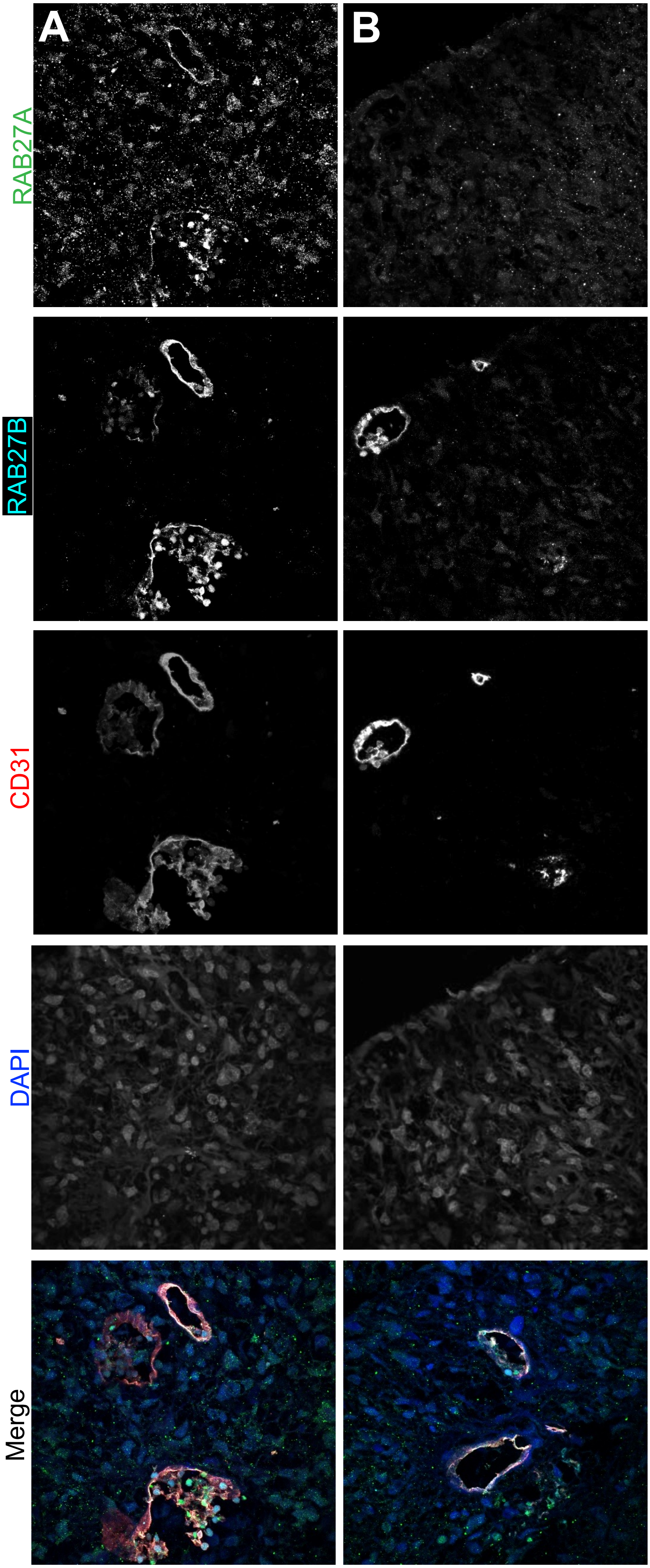


***Fig. S35***

**Supplemental Figure 35.** ***WHO grade IV astrocytoma tissue expresses RAB27A and RAB27B in CD31-positive endothelial cells.*** **(A,B)** Tumor tissues acquired from AmsBio for patient#211906.

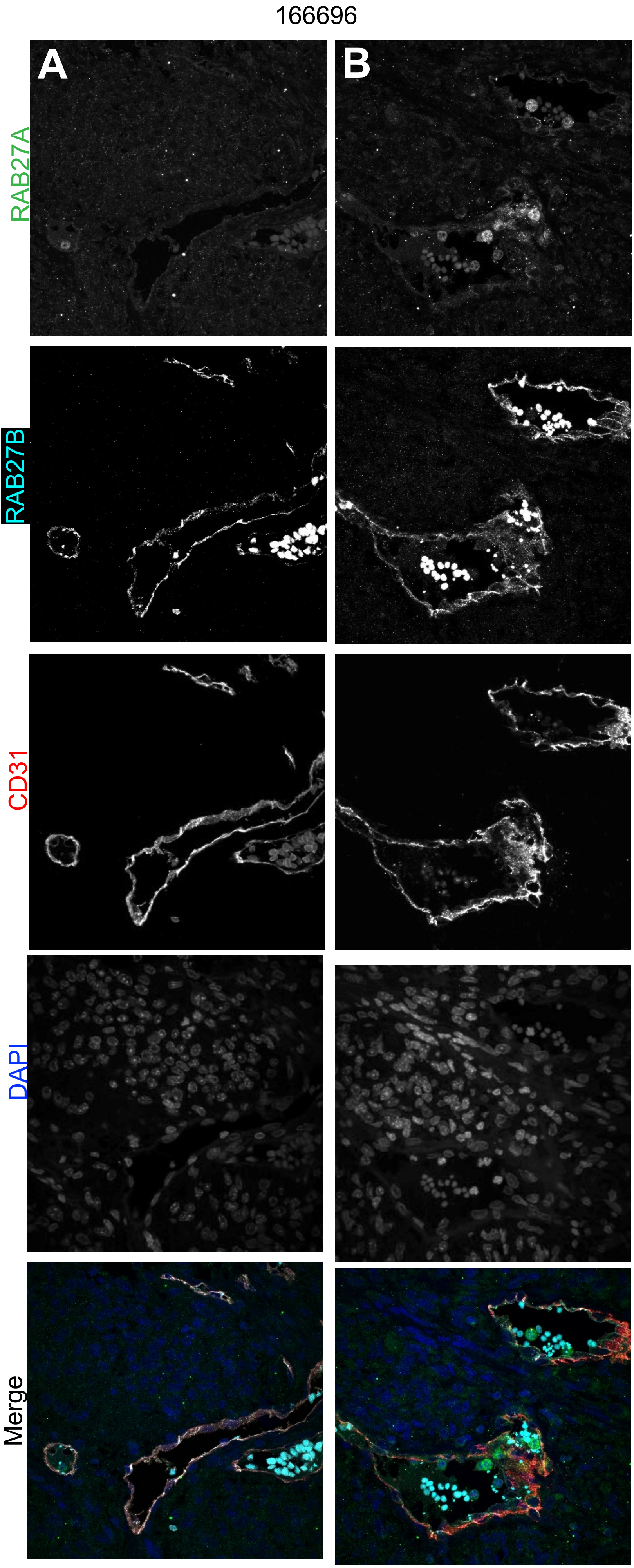


*Fig. S36*

**Supplemental Figure 36.** ***WHO grade IV astrocytoma tissues express RAB27A and RAB27B in the CD31-positive vasculature.*** **(A,B)** Tumor tissues acquired from AmsBio for patient#166696.

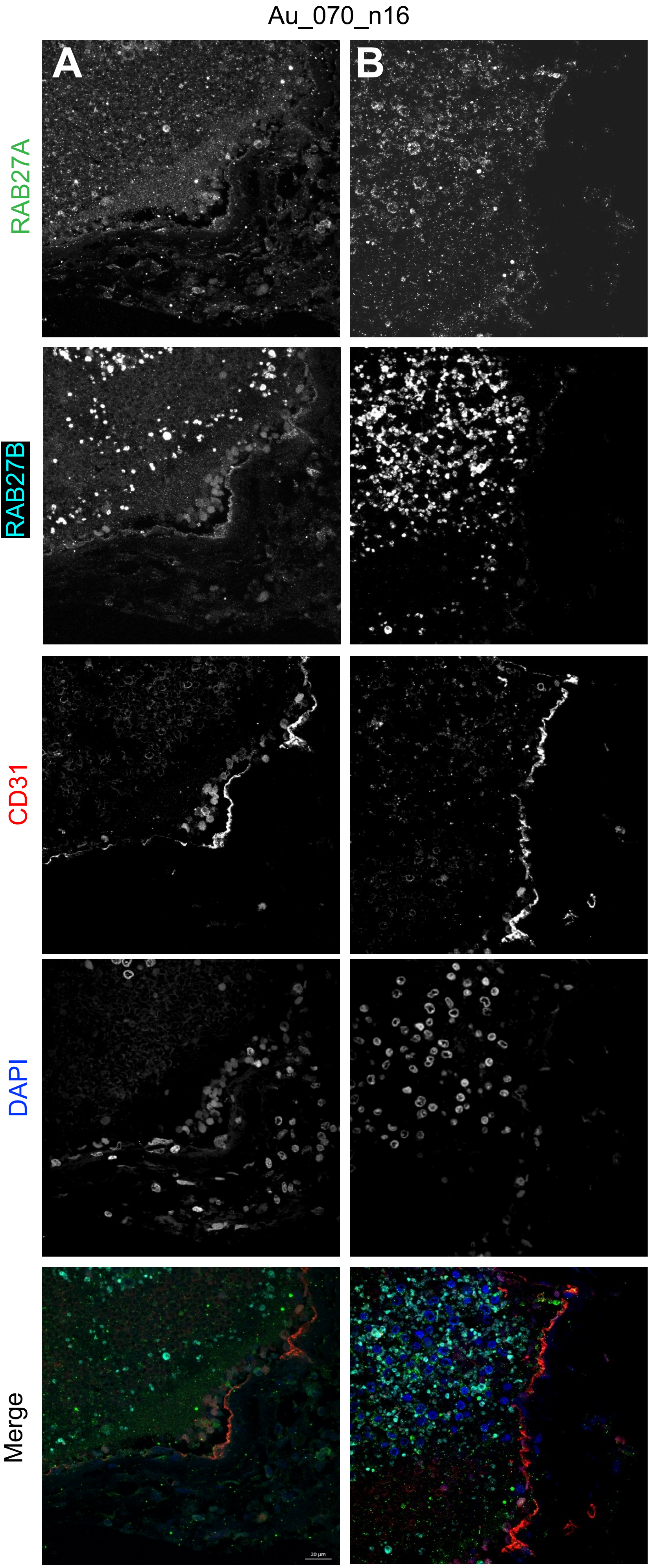


*Fig. S37*

**Supplemental Figure 37.** ***WHO grade IV astrocytoma tissues express RAB27A and RAB27B in the CD31-positive vasculature.*** **(A,B)** Tissues for patient#AU_070_N16.

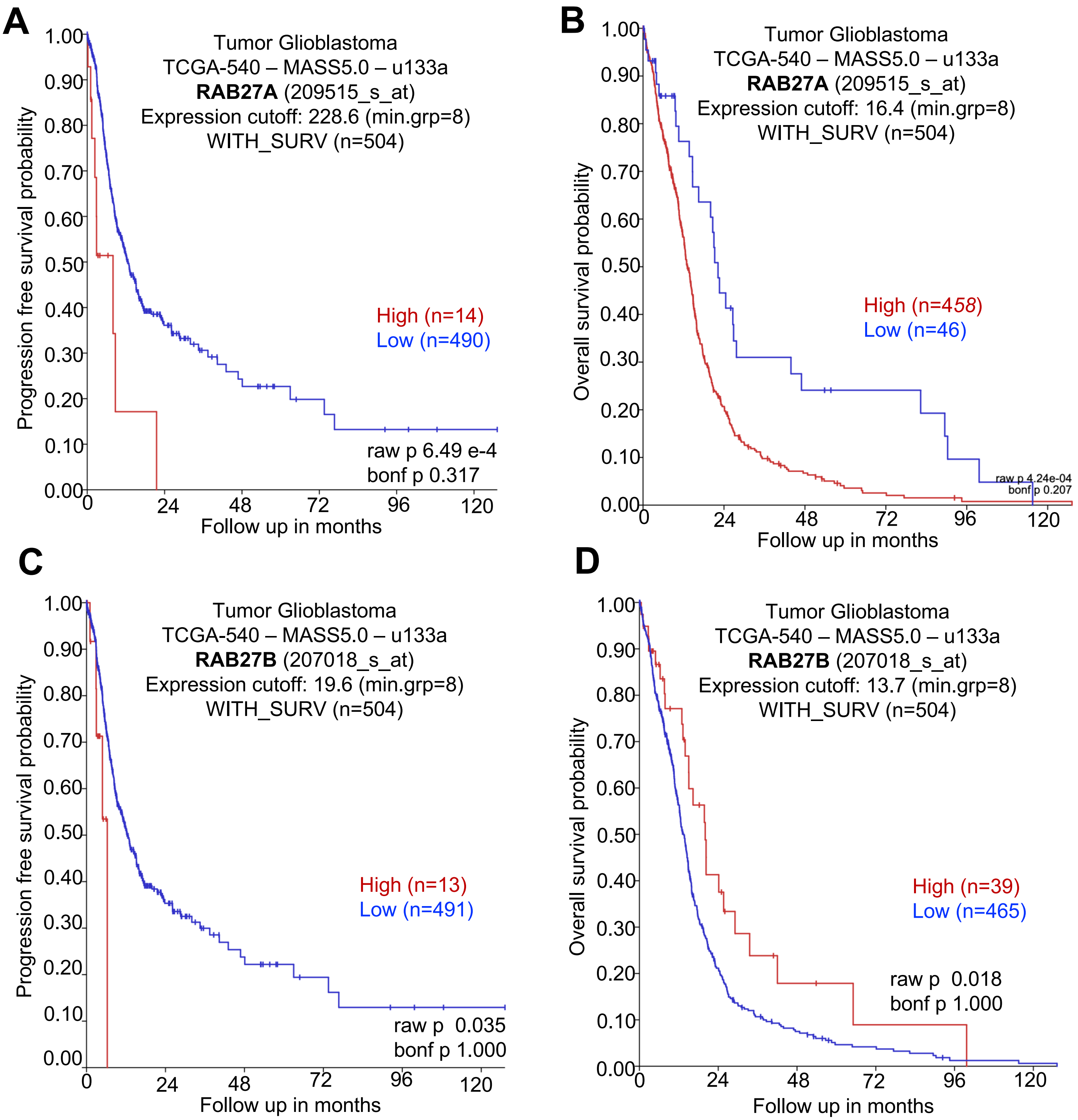


***Fig. S38***

**Supplemental Figure 38.** ***Low RAB27 expression in glioblastoma may influence some aspects of patients' survival.*** Kaplan-Meier survival curves based on RAB27A and RAB27B expression levels in patients diagnosed with glioblastoma (GBM). Global expression of RAB27 mRNA in bulk tumor tissue was included in the analysis and the expression cut-offs were arbitrarily chosen to explore the potential impact of RAB27 levels on patients' survival. **(A)** Progression free survival in GBM patients with low expression of RAB27A. **(B)** Overall survival of GBM patients with low expression of RAB27A. **(C)** Progression free survival in GBM patients with high expression of RAB27B. **(D)** Overall survival in GBM patients with high expression of RAB27B.

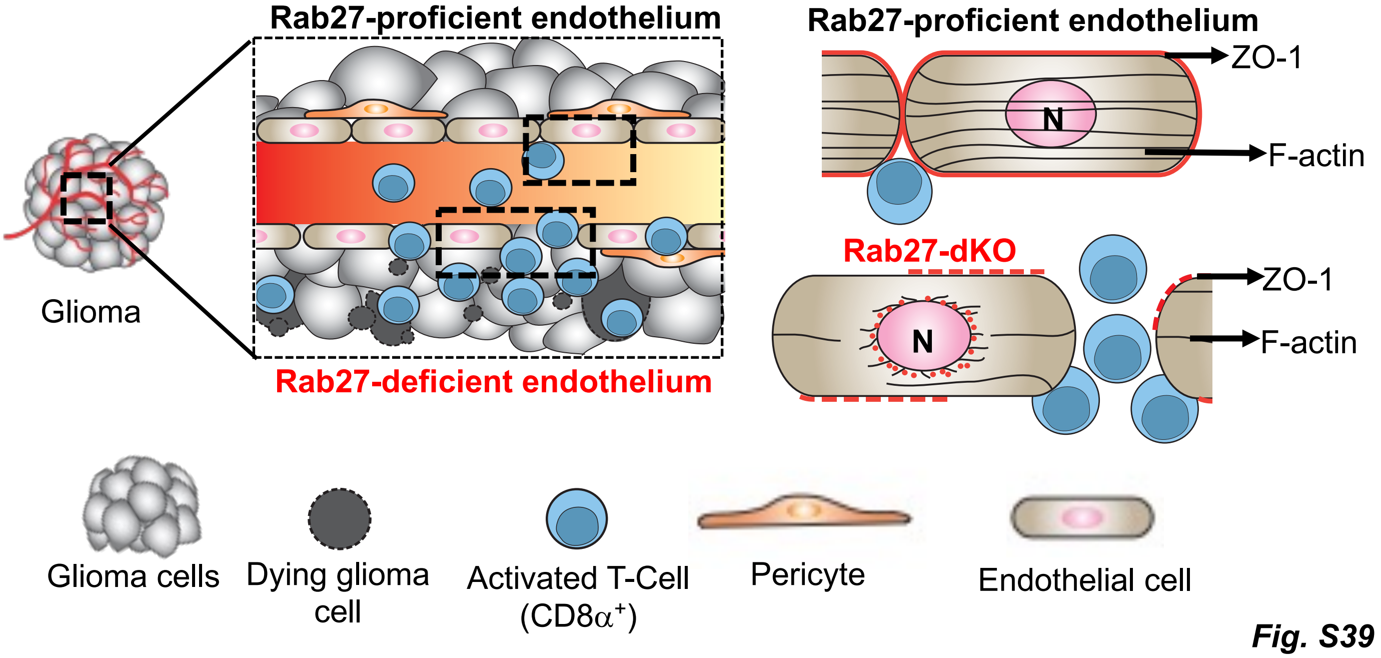


***Fig. S39***

**Supplemental Figure 39.** ***A model of Rab27-dependent regulation of vascular wall integrity and lymphocyte transmigration during brain tumorigenesis***. Rab27 is required for proper assembly of endothelial barrier in the brain. Rab27 influences actin filaments (F-actin) and tight junction (TJ) formation in endothelial cells, including expression and subcellular distribution of TJ proteins, such as ZO-1. In the absence of Rab27 activity tumor-associated endothelial cell phenotype changes and becomes permissive of the entry of CD8$\alpha^+$ T-cells into the tumor microenvironment. This change boosts the anti-tumor activity of adoptive T cell (CD8$\alpha^+$) transfer; see text for details. Abbreviations: Rab27-dKO - Rab27a/b double knock out cells deficient for Rab27 activity.
